# UM171 glues asymmetric CRL3–HDAC1/2 assembly to degrade CoREST corepressors

**DOI:** 10.1038/s41586-024-08532-4

**Published:** 2025-02-12

**Authors:** Megan J. R. Yeo, Olivia Zhang, Xiaowen Xie, Eunju Nam, N. Connor Payne, Pallavi M. Gosavi, Hui Si Kwok, Irtiza Iram, Ceejay Lee, Jiaming Li, Nicholas J. Chen, Khanh Nguyen, Hanjie Jiang, Zhipeng A. Wang, Kwangwoon Lee, Haibin Mao, Stefan A. Harry, Idris A. Barakat, Mariko Takahashi, Amanda L. Waterbury, Marco Barone, Andrea Mattevi, Steven A. Carr, Namrata D. Udeshi, Liron Bar-Peled, Philip A. Cole, Ralph Mazitschek, Brian B. Liau, Ning Zheng

**Affiliations:** 1 Department of Chemistry and Chemical Biology, Harvard University, Cambridge, MA, USA; 2 Broad Institute of MIT and Harvard, Cambridge, MA, USA; 3 Department of Pharmacology, University of Washington, Seattle, WA, USA; 4 Howard Hughes Medical Institute, University of Washington, Seattle, WA, USA; 5 Division of Genetics, Department of Medicine, Brigham and Women’s Hospital, Department of Biological Chemistry and Molecular Pharmacology, Harvard Medical School, Boston, MA, USA; 6 Center for Systems Biology, Massachusetts General Hospital, Boston, MA, USA; 7 Harvard T.H. Chan School of Public Health, Boston, MA, USA; 8 Krantz Family Center for Cancer Research, Massachusetts General Hospital, Boston, MA, USA; 9 Department of Biology and Biotechnology Lazzaro Spallanzani, University of Pavia, Pavia, Italy; 10 Department of Medicine, Harvard Medical School, Boston, MA, USA; 11 Present address: Desai Sethi Urology Institute & Sylvester Comprehensive Cancer Center, University of Miami Miller School of Medicine, Miami, FL, USA; 12 These authors contributed equally: Megan J. R. Yeo, Olivia Zhang, Xiaowen Xie Eunju Nam

## Abstract

UM171 is a potent agonist of ex vivo human haematopoietic stem cell self-renewal^1^. By co-opting KBTBD4, a substrate receptor of the CUL3–RING E3 ubiquitin ligase (CRL3) complex, UM171 promotes the degradation of the LSD1–CoREST corepressor complex, thereby limiting haematopoietic stem cell attrition^2,3^. However, the direct target and mechanism of action of UM171 remain unclear. Here we show that UM171 acts as a molecular glue to induce high-affinity interactions between KBTBD4 and HDAC1/2 to promote corepressor degradation. Through proteomics and chemical inhibitor studies, we identify the principal target of UM171 as HDAC1/2. Cryo-electron microscopy analysis of dimeric KBTBD4 bound to UM171 and the LSD1–HDAC1–CoREST complex identifies an asymmetric assembly in which a single UM171 molecule enables a pair of KELCH-repeat propeller domains to recruit the HDAC1 catalytic domain. One KBTBD4 propeller partially masks the rim of the HDAC1 active site, which is exploited by UM171 to extend the E3–neosubstrate interface. The other propeller cooperatively strengthens HDAC1 binding through a distinct interface. The overall CoREST–HDAC1/2–KBTBD4 interaction is further buttressed by the endogenous cofactor inositol hexakisphosphate, which acts as a second molecular glue. The functional relevance of the quaternary complex interaction surfaces is demonstrated by base editor scanning of *KBTBD4* and *HDAC1*. By delineating the direct target of UM171 and its mechanism of action, we reveal how the cooperativity offered by a dimeric CRL3 E3 can be leveraged by a small molecule degrader.

Degraders are small molecules capable of promoting the ubiquitination and degradation of proteins^4,5^. These compounds are classified into two categories: the monovalent molecular glues (glues) and the bifunctional proteolysis targeting chimeras (PROTACs). Besides their more drug-like properties, glue degraders are distinct from PROTACs by being capable of inducing high-affinity interactions between an E3 ubiquitin ligase and a neosubstrate without showing detectable affinity to at least one of these protein partners^6^. Although rapid progress has been made in the rational design of PROTACs, the development of glue degraders has been protracted due to poor understanding of their functional prerequisites and E3 scaffold preferences. The plant hormones auxin and jasmonate are the first documented glue degraders, which co-opt the F-box proteins—substrate receptors of CUL1–RING ligase (CRL1) complexes^7–9^. In human cells, the best characterized glue degraders include thalidomide and its derivatives, aryl-sulfonamides and CDK12 inhibitors^4^. Notably, these synthetic compounds all co-opt the CUL4–RING ligases (CRL4s), raising the question of whether other ubiquitin ligases can be reprogrammed by glues. CRL3s, in particular, represent the largest family of CRLs with nearly 200 substrate receptors. Its family members form constitutive homodimers that are exploited by endogenous substrates for cooperative binding^10–14^. Whether CRL3s can be leveraged by glue degraders, especially to exploit their intrinsic cooperativity, remains uncertain.

Small molecules that promote the expansion of haematopoietic stem cells have clinical applications for cell-based therapies^15,16^. UM171 was optimized from UM729, a compound identified as the top hit in a phenotypic screen for haematopoietic stem cell expansion^1^ (Fig. 1a). Despite its wide use and progression into human clinical trials, the mechanism of action of UM171 is unclear. More recently, UM171 was shown to induce degradation of lysine-specific histone demethylase 1a (LSD1) and CoREST (which is encoded by *RCOR1*)^2,3^. CoREST functions as a scaffold to recruit LSD1 and either the histone deacetylase HDAC1 or the paralogue HDAC2 at its two ends, forming the core LSD1–HDAC1/2–CoREST (LHC) corepressor complex^17^. After addition of UM171, CoREST and LSD1 are rapidly degraded by KBTBD4^2^, a BTB-KELCH E3 substrate adaptor belonging to the CRL3 family. Despite these advances, the direct target and mechanism of action of UM171 has remained unclear.

## UM171 degrades select HDAC1/2 complexes

**Extended Data Fig. 1 |:**
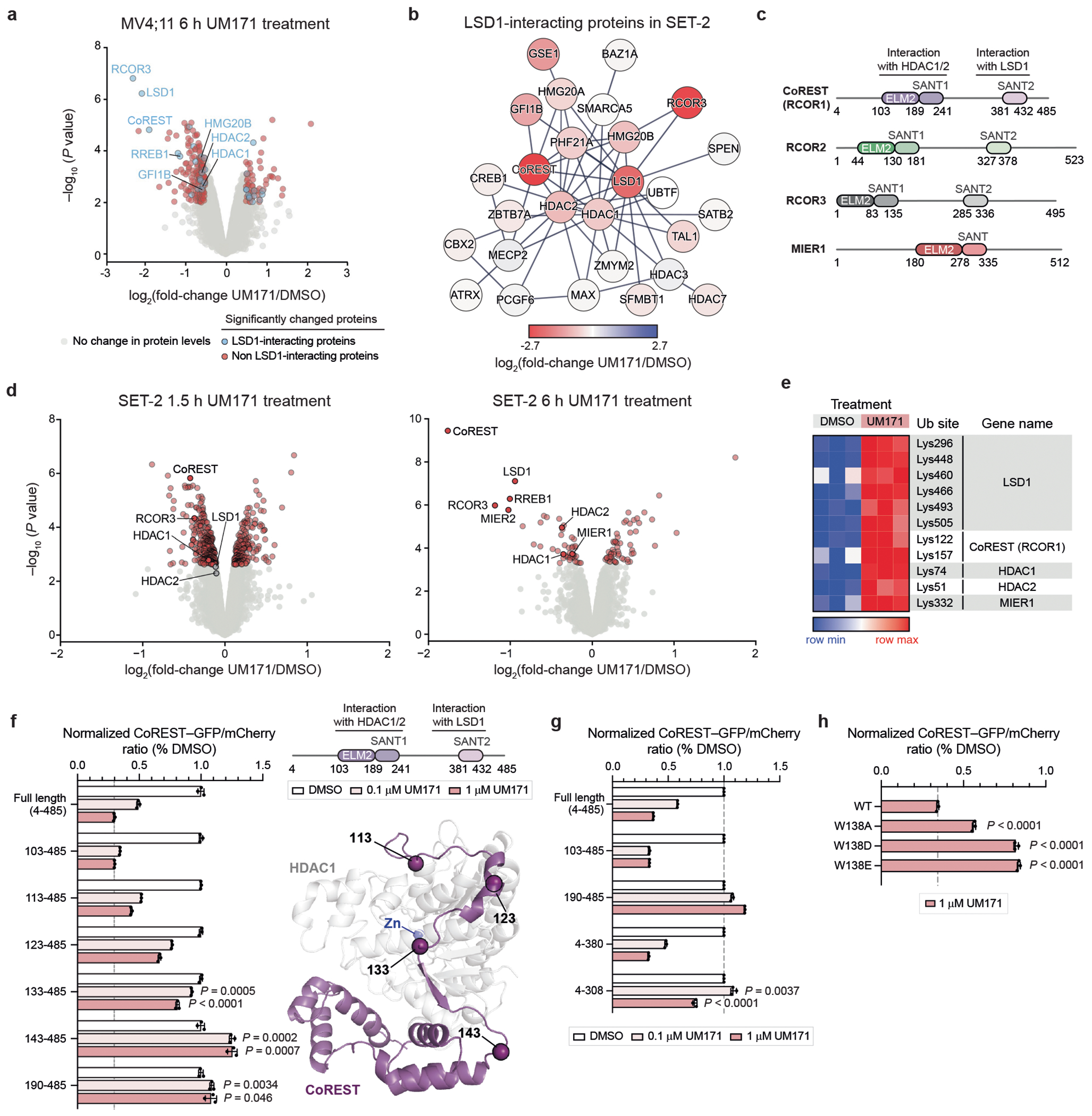
Supporting data for Fig. 1. **a**, Whole-proteome quantification in MV4;11 cells treated with DMSO (*n* = 3) or 1 μM UM171 (*n* = 3) for 6 h. Coloured dots show proteins with |log_2_(fold-change)| > 0.5 in UM171 versus DMSO treatment and *P* value < 0.01. Blue and red dots depict proteins enriched or absent in LSD1 co-IP/MS, respectively. **b**, STRING network of proteins enriched in LSD1 co-IP/MS in SET-2 cells. Colour scale depicts log_2_(fold-change) in UM171 treatment. **c**, Protein domain maps of CoREST (RCOR1), RCOR2, RCOR3, and MIER1. **d**, Whole-proteome quantification in SET-2 cells treated with DMSO (*n* = 3) or 1 μM UM171 (*n* = 3) for 1.5 h (left) and 6 h (right). Red dots show proteins with adjusted *P* value < 0.05 (Benjamini–Hochberg correction for multiple comparisons). **e**, Differential enrichment of selected ubiquitination Lys sites highlighted in Fig. 1d. Colour indicates row min and max values. Due to sequence homology between HDAC paralogues, several peptides corresponding to either HDAC1 or HDAC2 could not be definitely assigned (see Supplementary Data 4–6). **f**-**h**, Flow cytometry quantification of MOLM-13 cells expressing the indicated CoREST–GFP reporter treated with DMSO or UM171 for 24 h. Signal is normalized to DMSO treatment for each CoREST construct. In **f** (right), Pymol alpha-fold structure of HDAC1-CoREST with key positions and Zn highlighted. Data are mean ± s.d. of *n* = 3 biological replicates and representative of two independent experiments, and *P* values were calculated through two-tailed unpaired *t* tests for the comparisons indicated. For data in **a** and **d**, *P* values were calculated using two-sided empirical Bayes-moderated t tests. FACS-gating schemes are in Supplementary Fig. 1a.

**Fig. 1 |:**
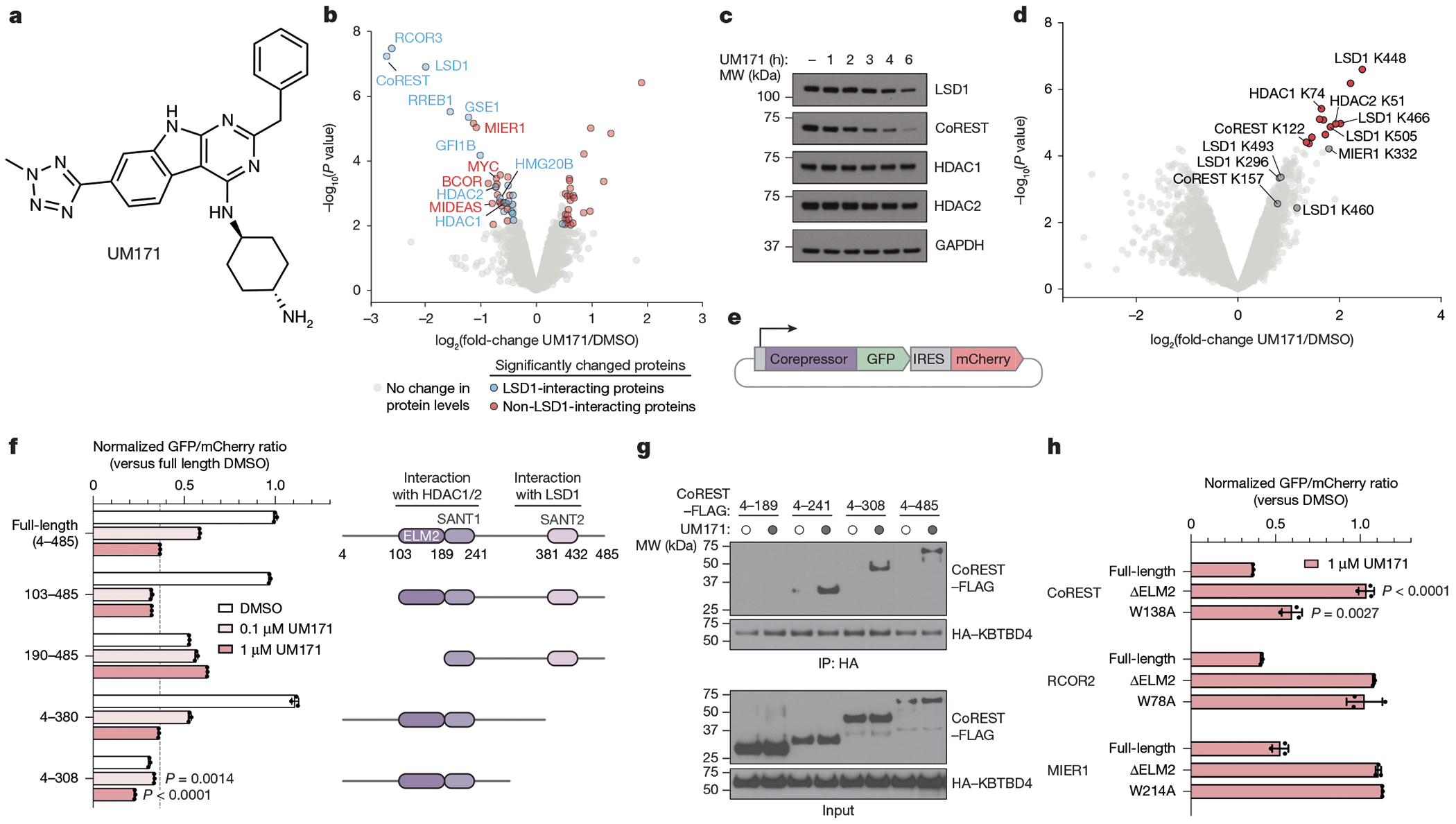
UM171-induced degradation of CoREST depends on HDAC1/2 interaction. **a**, The chemical structure of UM171. **b**, Whole-proteome quantification in SET-2 cells treated with DMSO (*n* = 3) or 1 μM UM171 (*n* = 3) for 6 h. The coloured dots show proteins with |log_2_[fold change]| > 0.5 and *P* < 0.01 in UM171 treatment. The blue and red dots depict proteins that are enriched or absent in LSD1 co-IP–MS, respectively. **c**, Immunoblot analysis of SET-2 cells treated with UM171 (1 μM) or DMSO for the indicated duration. **d**, Global ubiquitination site quantification (K-ε-GG peptides) in SET-2 cells treated with DMSO (*n* = 3) or 1 μM UM171 (*n* = 3) for 90 min. The red dots show sites with adjusted *P* < 0.05 (after Benjamini–Hochberg correction for multiple comparisons). Owing to sequence homology between the HDAC1/2 paralogues, several peptides corresponding to either HDAC1 or HDAC2 could not be definitely assigned (Supplementary Data 4–6). **e**, Schematic of corepressor constructs fused in-frame with GFP followed by an internal ribosome entry site (IRES) and mCherry stability reporter. **f**,**h**, Flow cytometry quantification of MOLM-13 cells treated with DMSO or UM171 for 24 h and expressing the indicated CoREST–GFP reporter (**f**) and the indicated corepressor–GFP reporter (**h**). **g**, Immunoblots of HA IP from 293T cells transfected with HA–KBTBD4 and the indicated CoREST–FLAG construct and treated with UM171 (1 μM) or DMSO for 1 h and MLN4924 (1 μM) for 3 h. The results in **c** and **f**–**h** are representative of two independent experiments. For **f** and **h**, data are mean ± s.d. of *n* = 3 biological replicates. *P* values were calculated using two-tailed unpaired *t*-tests for the indicated comparisons (**f** and **h**) and two-sided empirical Bayes-moderated *t*-tests (**b** and **d**). FACS gating schemes and uncropped blots are shown in Supplementary Figs. 1a and 2, respectively. HA, hemagglutinin; IP, immunoprecipitation; MW, molecular weight.

To identify the direct binding target of UM171, we first determined the repertoire of proteins depleted by UM171 treatment. We conducted a global proteomics analysis in two UM171-sensitive cell lines, SET-2 and MV4;11, after vehicle (DMSO) or UM171 treatment (6 h, 1 μM). LSD1 and two CoREST paralogues, CoREST (RCOR1) and RCOR3, were the most significantly depleted proteins after UM171 treatment (Fig. 1b, Extended Data Fig. 1a and Supplementary Data 1 and 2). RCOR2, another CoREST paralogue, is not expressed in these cell lines. Several other highly downregulated proteins are components of the broader LHC complex (for example, RREB1, GSE1, HMG20B), suggesting extensive collateral degradation^18^. To assess which downregulated proteins are direct versus collateral targets of UM171–KBTBD4, we cross-compared to LSD1-interacting proteins identified by co-immunoprecipitation (co-IP)–mass spectrometry (MS)^19^ (Supplementary Data 3). As expected, many depleted proteins associate with LHC (Fig. 1b (blue dots) and Extended Data Fig. 1a,b). However, MIER1 was highly depleted but did not co-immunoprecipitate with LSD1 (Fig. 1b (red dots)), indicating that it might be a distinct neosubstrate of UM171–KBTBD4 independent of LSD1 association–as supported by a recent study^20^. Notably, all three CoREST paralogues and MIER1 contain an ELM2-SANT tandem domain^21^ (Extended Data Fig. 1c). Collectively, UM171 promotes downregulation of several corepressors containing an ELM2-SANT domain as well as associated complex members.

A time-course analysis of UM171 treatment revealed rapid depletion of CoREST (Fig. 1c). Consistent with previous work, CoREST depletion was followed by potent, albeit delayed, depletion of LSD1^2^. Depletion of HDAC1 and HDAC2, the other interchangeable core members of LHC, was modest at the early timepoints tested, matching our global proteomics (Fig. 1b, Extended Data Fig. 1d and Supplementary Data 4). However, ubiquitin proteomics using K-ε-GG peptide enrichment showed that CoREST, LSD1, HDAC1 and HDAC2 are ubiquitinated at an early timepoint of UM171 treatment, suggesting that additional mechanisms mitigate HDAC1/2 degradation (Fig. 1d, Extended Data Fig. 1e and Supplementary Data 5 and 6). Together, these data support that CoREST is a direct neosubstrate of UM171–KBTBD4.

We next defined the region(s) of CoREST that are necessary for UM171-induced degradation by using a well-established fluorescent reporter system, in which full-length (considered amino acids 4–485) or truncated CoREST variants are fused in-frame with GFP followed by an internal ribosome entry site (IRES) and mCherry^22^ (Fig. 1e). While deletion of the N-terminal 4–103 amino acids or the SANT2 domain (amino acids 380–485) had minimal impact, deletion of amino acids 4–189 completely blocked CoREST–GFP degradation by UM171 (Fig. 1f and Extended Data Fig. 1f), providing evidence that the ELM2 domain is necessary. A larger C-terminal deletion construct, CoREST(4–308)–GFP, exhibited reduced baseline stability^23,24^. Nonetheless, UM171 treatment still significantly decreased CoREST(4–308)–GFP levels (Extended Data Fig. 1g). Co-IP experiments demonstrated that CoREST(4–241)–FLAG is sufficient to interact with HA–KBTBD4, whereas CoREST(4–189)–FLAG cannot (Fig. 1g). Finally, using the fluorescent reporter system, we observed that the ELM2 domains were also required for UM171-induced degradation of MIER1 and RCOR2 (Fig. 1h). Together, these results show that the ELM2 domains are necessary for UM171-induced degradation of corepressors.

In all of the tested corepressors, the ELM2-SANT domain mediates complexation with either HDAC1 or its paralogue HDAC2^21^ (Extended Data Fig. 1c). We reasoned that not only the ELM2-SANT domain but also HDAC1 and/or HDAC2 might be necessary for UM171 action. Mutation of MIER1 Trp214 to alanine (W214A) has been previously shown to disrupt the MIER1–HDAC1 interface^25^. MIER1(W214A) and the corresponding Trp to Ala mutants, CoREST(W138A) and RCOR2(W78A), exhibited significantly decreased UM171-induced degradation (Fig. 1h). The rescue afforded by W138A in CoREST was only partial; however, the more disruptive Trp to Asp/Glu mutations fully blocked degradation (Extended Data Fig. 1h). Together, our findings support that interactions between HDAC1 and/or HDAC2 with corepressors are critical for UM171-mediated corepressor degradation.

## HDAC1/2 mediates LHC–KBTBD4 complexation

**Extended Data Fig. 2 |:**
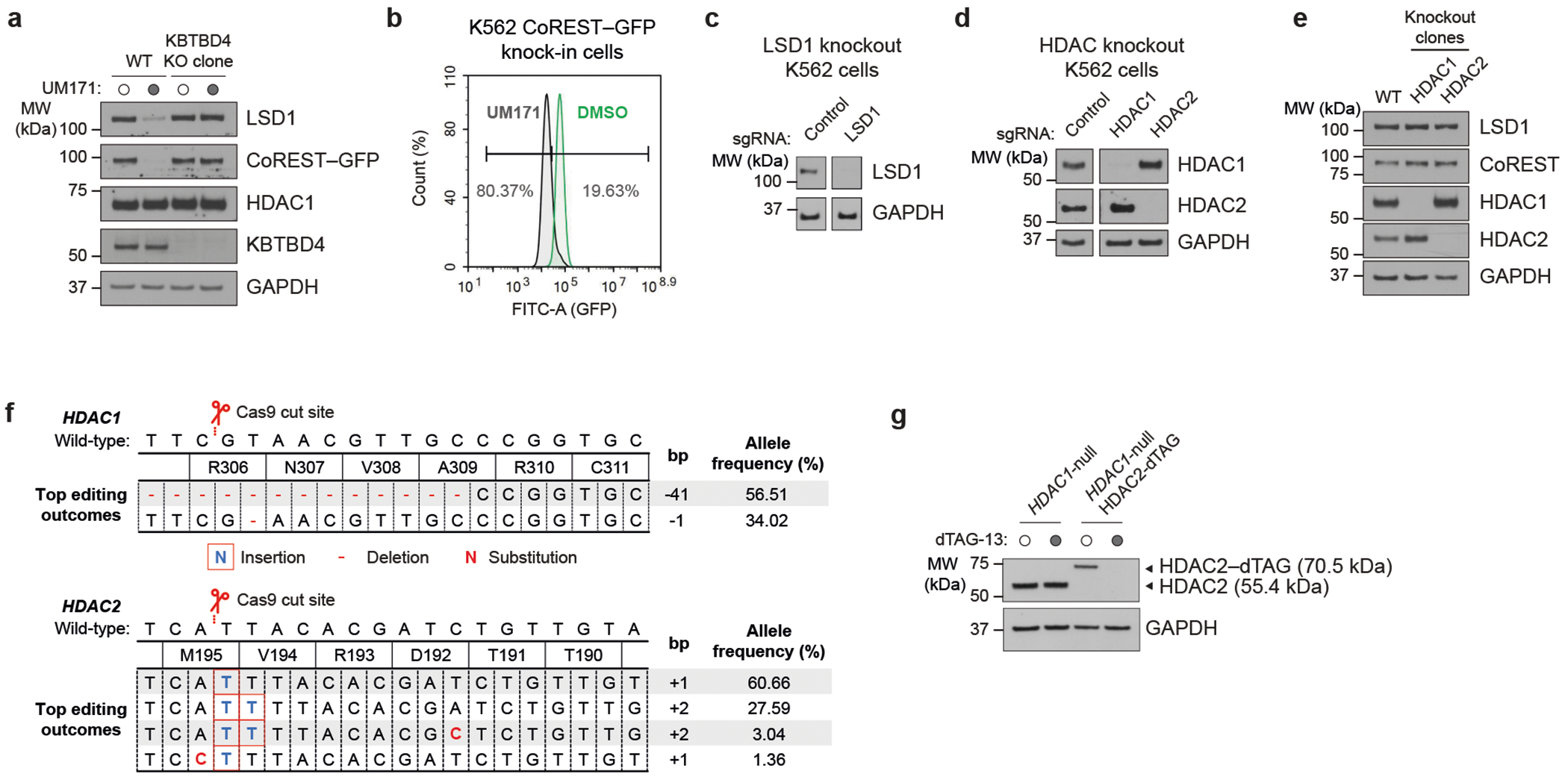
Supporting cell line model data for Fig. 2. **a**, Immunoblots in wild-type K562 CoREST–GFP knock-in cells and a clonal cell line with KBTBD4 knockout treated with DMSO or UM171 (1 μM) for 6 h. **b**, Flow cytometry for GFP signal in K562 CoREST–GFP cells treated with DMSO (green) or 1 μM UM171 (grey) for 6 h. Gate was determined where 1% of the DMSO condition are considered FITC^−^. **c**,**d**, Immunoblots in K562 CoREST–GFP cells after transduction of the indicated sgRNAs. **e**, Immunoblots showing CoREST–GFP, HDAC1, and HDAC2 in K562 *HDAC1*-null and *HDAC2*-null CoREST–GFP clonal cell lines. **f**, Allele frequency analysis for K562 CoREST–GFP *HDAC1*-null (top) and *HDAC2*-null (bottom) clonal cell lines. Genotyping was performed once. **g**, Immunoblots showing HDAC2–dTAG and GAPDH in K562 CoREST–GFP *HDAC1*-null HDAC2–dTAG cells treated with DMSO or dTAG-13 (500 nM) for 2 h. Data in **a-e**, and **g** are representative of two independent experiments. FACS-gating schemes and uncropped blots are in Supplementary Figs. 1b, 5, respectively. KO, knock out; MW, molecular weight.

**Extended Data Fig. 3 |:**
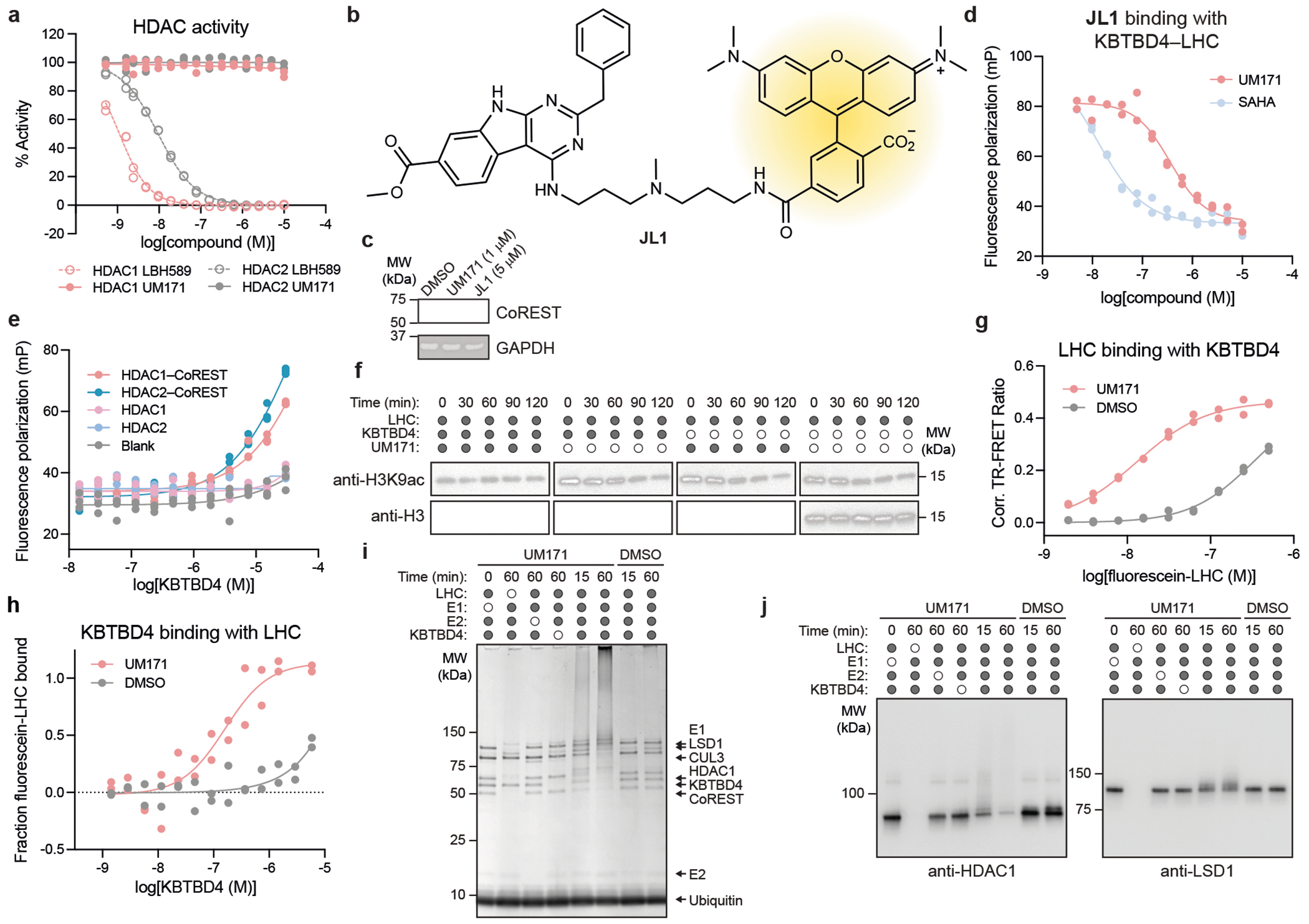
Supporting biochemistry data for Fig. 2. **a**, Relative enzyme activity for indicated HDAC in the presence of varying concentrations of the indicated compounds. **b**, Chemical structure of **JL1**. **c**, Immunoblots for K562 cells treated with UM171 or **JL1** at indicated concentrations for 24 h. **d**, Fluorescence polarization of **JL1** with KBTBD4 and LHC in the presence of varying concentrations of SAHA or UM171 (*n* = 2 biological replicates). **e**, Fluorescence polarization of **JL1** with KBTBD4 and InsP_6_ in the presence or absence of indicated proteins (*n* = 3 biological replicates). **f**, Immunoblots for in vitro deacetylation assays of LHC with H3K9ac modified mononucleosomes staining with antibodies for H3K9ac and total H3 under the indicated conditions. All timepoints for a given experimental condition were run on a single gel. All samples across conditions were derived from the same experiment and were run in parallel. **g**, TR-FRET signal between anti-His CoraFluor-1-labelled antibody with His-KBTBD4 and varying concentrations of fluorescein-LHC in the presence of DMSO or 10 μM UM171 (*n* = 2 biological replicates). *K_D_* values of the LHC-KBTBD4 interactions in the absence and presence of UM171 are 351 nM and 13 nM, respectively. **h**, Microscale thermophoresis of fluorescein-LHC with varying concentrations of KBTBD4 in the presence and absence of 50 μM UM171 (*n* = 2 biological replicates). **i**, Coomassie staining for in vitro ubiquitination assays of CRL3^KBTBD4^ with fluorescein-LHC in the presence of DMSO or UM171 (10 μM). **j**, Immunoblots for HDAC1 (left) and LSD1 (right) for in vitro ubiquitination assays of CRL3^KBTBD4^ with fluorescein-LHC in the presence of DMSO or UM171 (10 μM). Data in **a**, **c**, **e**, and **g** are representative of two independent experiments. Data in **f** are representative of *n* = 2 biological replicates. Results in **i** and **j** are representative of *n* = 3 biological replicates. Uncropped blots can be found in Supplementary Fig. 6. Corr., corrected; MW, molecular weight.

**Fig. 2 |:**
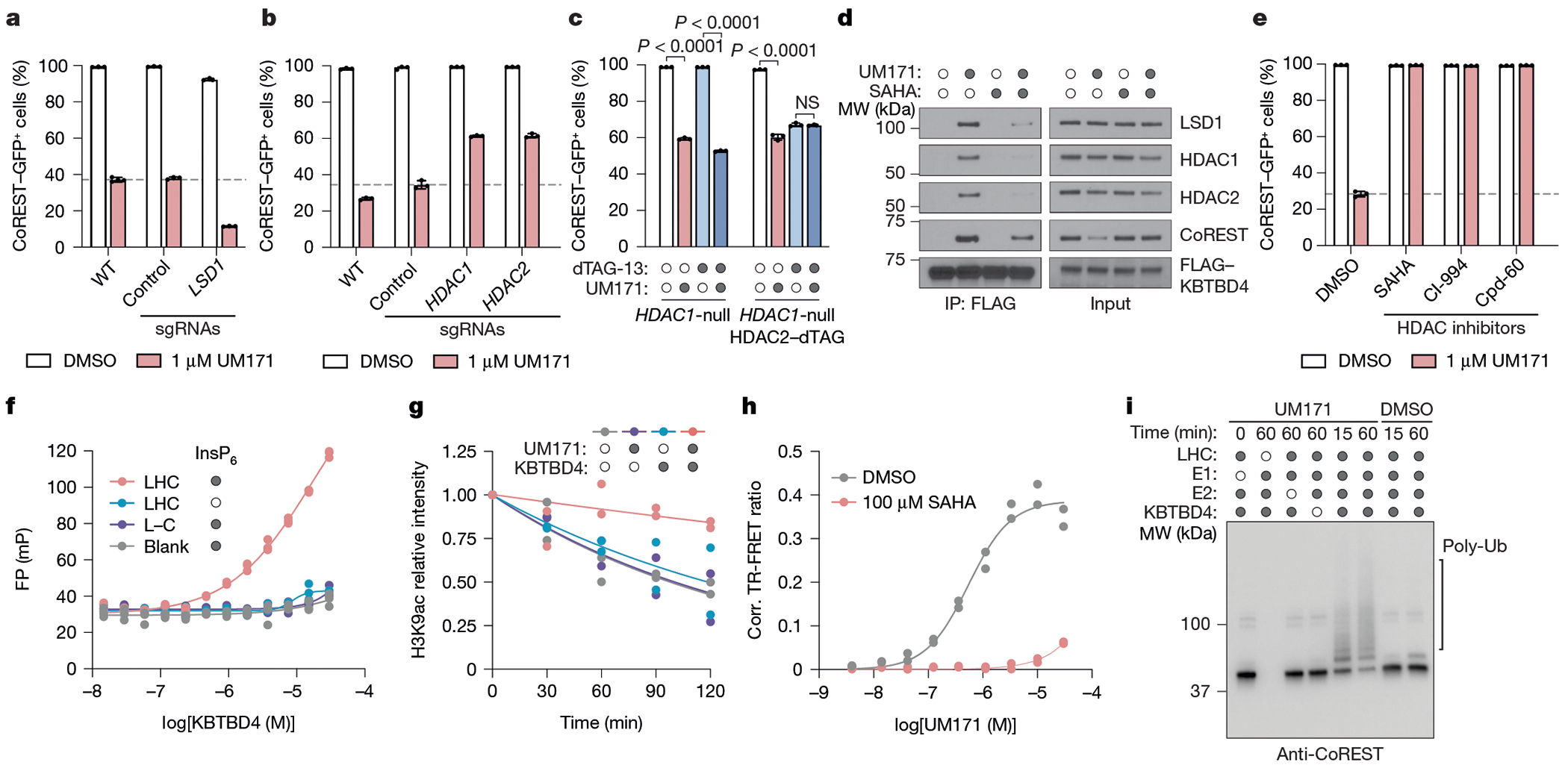
HDAC1 mediates LHC–UM171–KBTBD4 ternary complex formation. **a**–**c**,**e**, Flow cytometry quantification of K562 CoREST–GFP cells that were transduced with the indicated sgRNAs (*LSD1* (**a**) and *HDAC1* and *HDAC2* (**b**)) after treatment with DMSO or UM171 (1 μM) for 24 h (**a**,**b**), K562 *HDAC1*-null HDAC2–dTAG CoREST–GFP cells after treatment with dTAG-13 (500 nM) or DMSO for 2 h followed by either UM171 (1 μM) or DMSO for 24 h (**c**), and K562 CoREST–GFP cells treated with the indicated HDAC inhibitors (10 μM) for 12 h followed by UM171 (1 μM) for 24 h (**e**). *P* values were calculated using two-tailed unpaired *t*-tests for the indicated comparisons. **d**, FLAG IP immunoblot analysis of K562 cells expressing FLAG–KBTBD4 and treated with UM171 (5 μM), SAHA (10 μM), or DMSO, and MLN4924 (1 μM). **f**, FP of **JL1** with KBTBD4 in the presence or absence of LHC or LSD1–CoREST (L–C) and InsP_6_ (50 μM). *n* = 3 biological replicates. **g**, Quantification of LHC deacetylase activity on H3K9ac-modified mononucleosomes in the presence or absence of UM171 (10 μM) and/or KBTBD4. *n* = 2 biological replicates. See also Extended Data Fig. 3f. **h**, The TR-FRET signal between fluorescein–LHC and anti-His CoraFluor-1-labelled antibody with His–KBTBD4 in the presence of varying concentrations of UM171. *n* = 2 biological replicates. **i**, CoREST immunoblot analysis of in vitro ubiquitination assays of CRL3^KBTBD4^ with fluorescein–LHC in the presence of DMSO or UM171 (10 μM). *n* = 3 biological replicates. For **a**–**c** and **e**, data are mean ± s.d. of *n* = 3 biological replicates and are representative of two independent experiments. For **d**, **f** and **h**, data are representative of two independent experiments. FACS gating schemes and uncropped blots are shown in Supplementary Figs. 1b and 3, respectively. Corr., corrected; FP, fluorescence polarization; MW, molecular weight; NS, not significant.

To further investigate the mechanistic involvement of LSD1, HDAC1 and HDAC2 in UM171-mediated CoREST degradation, we engineered a K562 knock-in cell line with GFP fused to the C terminus of endogenous CoREST. Treatment of these cells with UM171 led to rapid KBTBD4-dependent CoREST–GFP depletion (Extended Data Fig. 2a,b). CRISPR knockout of *LSD1* did not rescue CoREST–GFP depletion by UM171, showing that LSD1 is not required for UM171 action (Fig. 2a and Extended Data Fig. 2c). By contrast, CRISPR knockout of *HDAC1* or *HDAC2* partially rescued CoREST–GFP depletion by UM171 (Fig. 2b and Extended Data Fig. 2d). We posited that the partial rescue is due to functional redundancy between the two HDAC paralogues in complexing with CoREST^26^. As constitutive *HDAC1/HDAC2* double knockout is lethal^26^, we engineered an *HDAC1*-null cell line with the dTAG degron tag^27^ knocked in-frame with HDAC2 to induce conditional *HDAC1/HDAC2* double knockout after addition of an appropriate ligand (for example, dTAG-13) (Extended Data Fig. 2e–g). Treatment with dTAG-13 alone led to rapid HDAC2 depletion and partial CoREST destabilization (Fig. 2c and Extended Data Fig. 2g). Notably, pretreatment with dTAG-13 further blocked CoREST degradation by UM171, demonstrating that either HDAC1/2 paralogue can mediate UM171 action. In support, co-IP of FLAG–KBTBD4 could retrieve both HDAC1 and HDAC2 in the presence of UM171, showing that both paralogues can associate with KBTBD4 (Fig. 2d). Lastly, pretreatment with HDAC active-site inhibitors^28^–including suberoylanilide hydroxamic acid (SAHA), CI-994 and Cpd-60^29^–also blocked CoREST–GFP degradation as well as co-IP of FLAG–KBTBD4 with CoREST, HDAC1 and HDAC2 induced by UM171 (Fig. 2d,e). However, UM171 had no impact on recombinant HDAC1/2 enzymatic activity (Extended Data Fig. 3a). Together, these data demonstrate that HDAC1/2 and their accessible active sites are required for UM171-induced degradation of CoREST while LSD1 is dispensable.

We next sought to determine whether UM171 is sufficient to induce ternary complex formation between KBTBD4 and members of the LHC complex^17,30^. Fluorescence polarization (FP) assays using a derivative of UM171 conjugated to tetramethylrhodamine, **JL1**, showed binding of **JL1** only in the presence of both KBTBD4 and LHC together, and furthermore only in the presence of inositol hexakisphosphate (InsP_6_) (Fig. 2f and Extended Data Fig. 3b–d). InsP_6_ has been previously shown to stabilize the interaction between HDAC1/2 and their cognate corepressors^21,31,32^. Accordingly, all of the subsequent experiments were conducted with 50 μM InsP_6_ unless otherwise noted. We also purified complexes containing full-length HDAC1 or HDAC2 associated with CoREST, which exhibited binding to **JL1** in a KBTBD4-dependent manner (Extended Data Fig. 3e), further supporting that HDAC1/2 are functionally redundant for UM171 action. By contrast, **JL1**–KBTBD4 binding was not observed with LSD1–CoREST, HDAC1 or HDAC2 alone (Fig. 2f and Extended Data Fig. 3e). Moreover, addition of SAHA dose-dependently blocked FP with a half-maximum inhibitory concentration comparable to its affinity for HDAC1/2^32^ (Extended Data Fig. 3d). Lastly, UM171 inhibited LHC deacetylase activity on recombinant nucleosomes only in the presence of KBTBD4 (Fig. 2g and Extended Data Fig. 3f), suggesting that complexation with the E3 obstructs the HDAC1 active site.

To directly assess the association between KBTBD4 and LHC, we used time-resolved Förster resonance energy transfer (TR-FRET) with labelled protein complexes. An ectopic cysteine residue was introduced at the N terminus of CoREST (amino acids 86-485) and selectively labelled with fluorescein (Methods) while His–KBTBD4 was labelled in situ with an anti-His CoraFluor-1 antibody^32,33^. UM171 induced TR-FRET signal in a dose-dependent manner, indicating association between fluorescein–LHC and His–KBTBD4 with an apparent half-maximum effective concentration of 542 nM under the experimental conditions (Fig. 2h). Co-treatment with SAHA blocked UM171-induced LHC–KBTBD4 association. Dose–response titration of fluorescein–LHC against His–KBTBD4 in the presence of UM171 and InsP_6_ at saturating concentrations yielded a *K*_D_ of 13 nM for the UM171-mediated LHC–KBTBD4 interaction (Extended Data Fig. 3g)—an approximately 25-fold increase from their UM171-independent basal affinity. This robust enhancement by UM171 is consistent with that observed by microscale thermophoresis assays (Extended Data Fig. 3h). Lastly, we established that reconstituted CRL3^KBTBD4^ is sufficient to mediate ubiquitination of LHC in vitro. In this system, ubiquitination of CoREST and HDAC1, but not LSD1, was significantly potentiated by addition of UM171 (Fig. 2i and Extended Data Fig. 3i,j). Collectively, these results demonstrate that UM171 stabilizes a ternary complex with KBTBD4 and HDAC1/2–CoREST, exhibiting highly cooperative binding and weak affinity to either KBTBD4 or LHC alone. Importantly, we establish the critical roles of HDAC1/2 and InsP_6_ in mediating complex formation, defining the minimal components necessary to reconstitute the complex for structural analysis.

## Cryo-EM structure of KBTBD4–UM171–LHC

**Extended Data Fig. 4 |:**
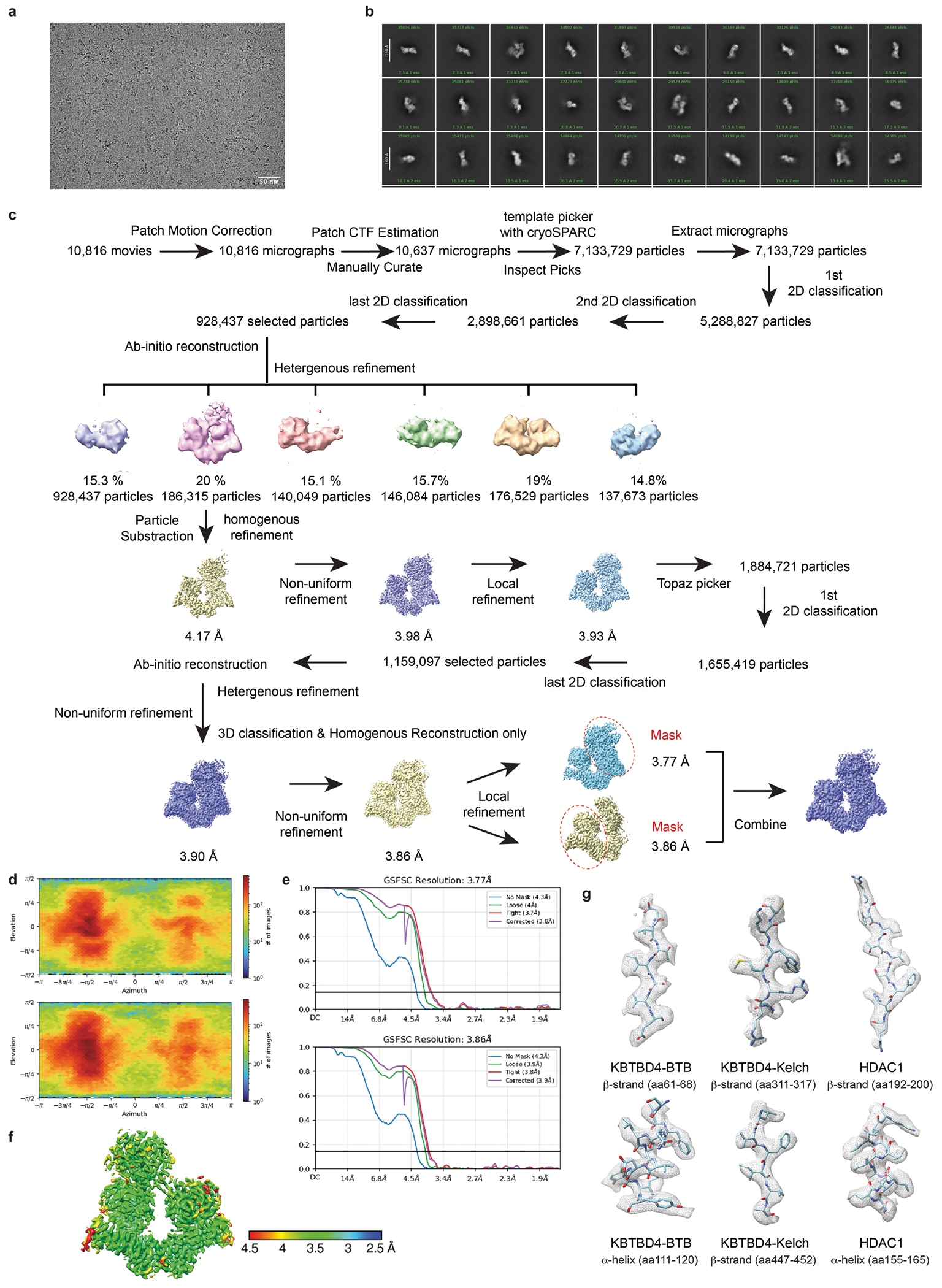
Cryo-EM data processing for the KBTBD4-UM171-LHC complex. **a**, A representative cryo-EM micrograph out of 10,637 micrographs; scale bar 50 nm. **b**, Typical 2D averages of the cryo-EM dataset. **c**, The flowchart of single particle analysis of the KBTBD4-UM171-LHC complex. **d**, The angular distribution of particles used in the final reconstruction. **e**, Fourier shell correlation (FSC) curves for KBTBD4-UM171-LHC. At the Gold-standard threshold of 0.143, the resolution is 3.77–3.86 Å. **f**, Local resolution map of the KBTBD4-UM171-LHC complex from 2.5 to 4.5 Å. **g**, Density maps of representative regions of the KBTBD4-UM171-LHC complex fit with the structural model shown in sticks.

**Extended Data Fig. 5 |:**
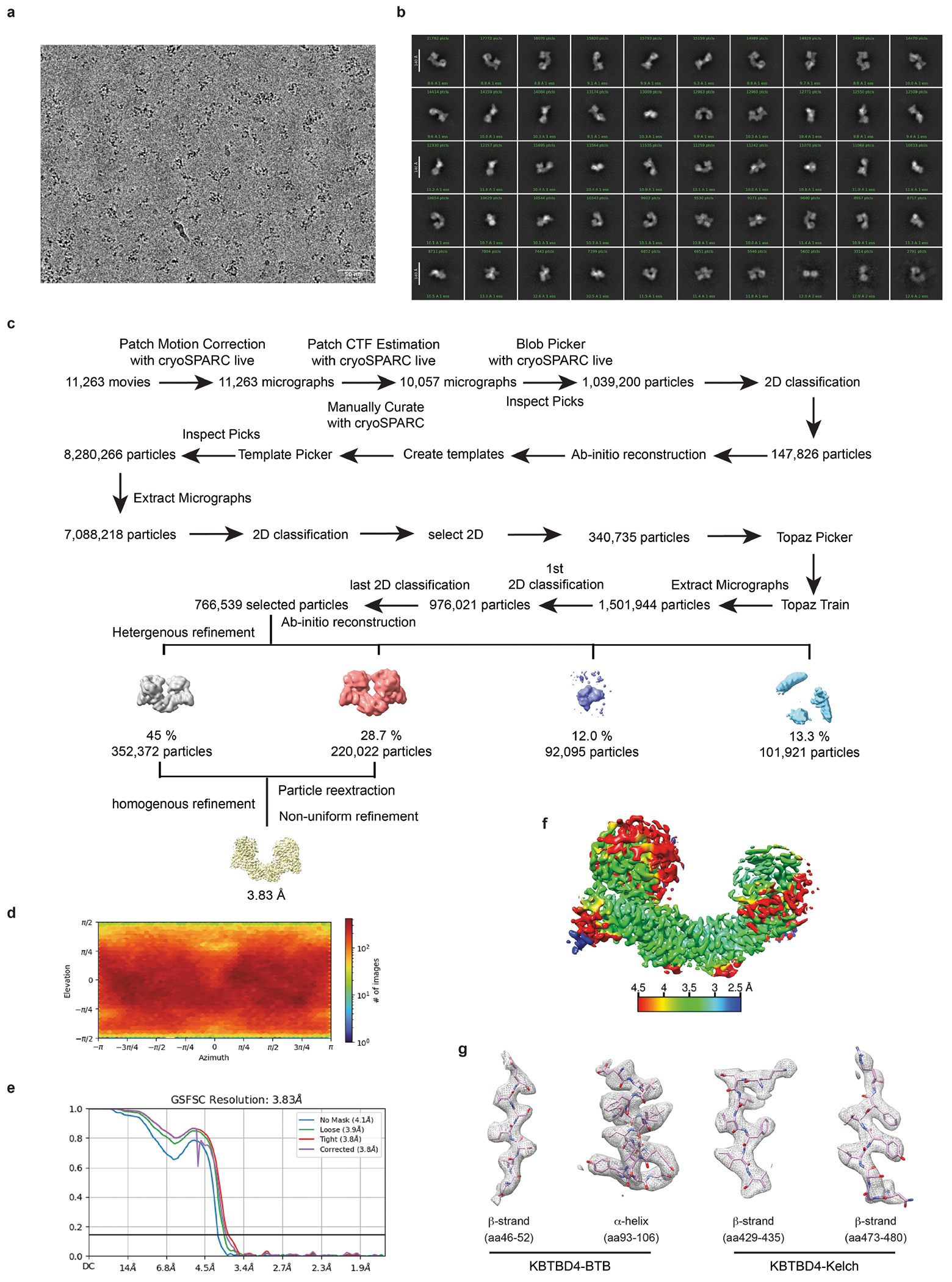
Cryo-EM data processing for the apo form of KBTBD4. **a**, A representative cryo-EM micrograph out of 10,057 micrographs; scale bar 50 nm. **b**, Typical 2D averages of the cryo-EM dataset. **c**, The flowchart of single particle analysis of the apo KBTBD4 complex. **d**, The angular distribution of particles used in the final reconstruction. **e**, Fourier shell correlation (FSC) curves for KBTBD4. At the Gold-standard threshold of 0.143, the resolution is 3.83 Å. **f**, Local resolution map of the KBTBD4 apo dimer from 2.5 to 4.5 Å. **g**, Density maps of representative regions of the homo-dimeric KBTBD4 complex fit with the structural model shown in sticks.

To resolve the mechanism of action of UM171, we next assembled the KBTBD4–UM171–LHC complex in the presence of InsP_6_ and determined its structure using cryo-electron microscopy (cryo-EM) at an average resolution of 3.77 Å (Extended Data Fig. 4 and Extended Data Table 1). KBTBD4 and HDAC1 are well resolved in the three-dimensional (3D) reconstruction map, whereas only partial densities are visible for the ELM2-SANT1 domain of CoREST in the rest of LHC. For comparison purposes, we also determined the cryo-EM structure of KBTBD4 in its apo form at a resolution of 3.83 Å (Extended Data Fig. 5 and Extended Data Table 1).

**Fig. 3 |:**
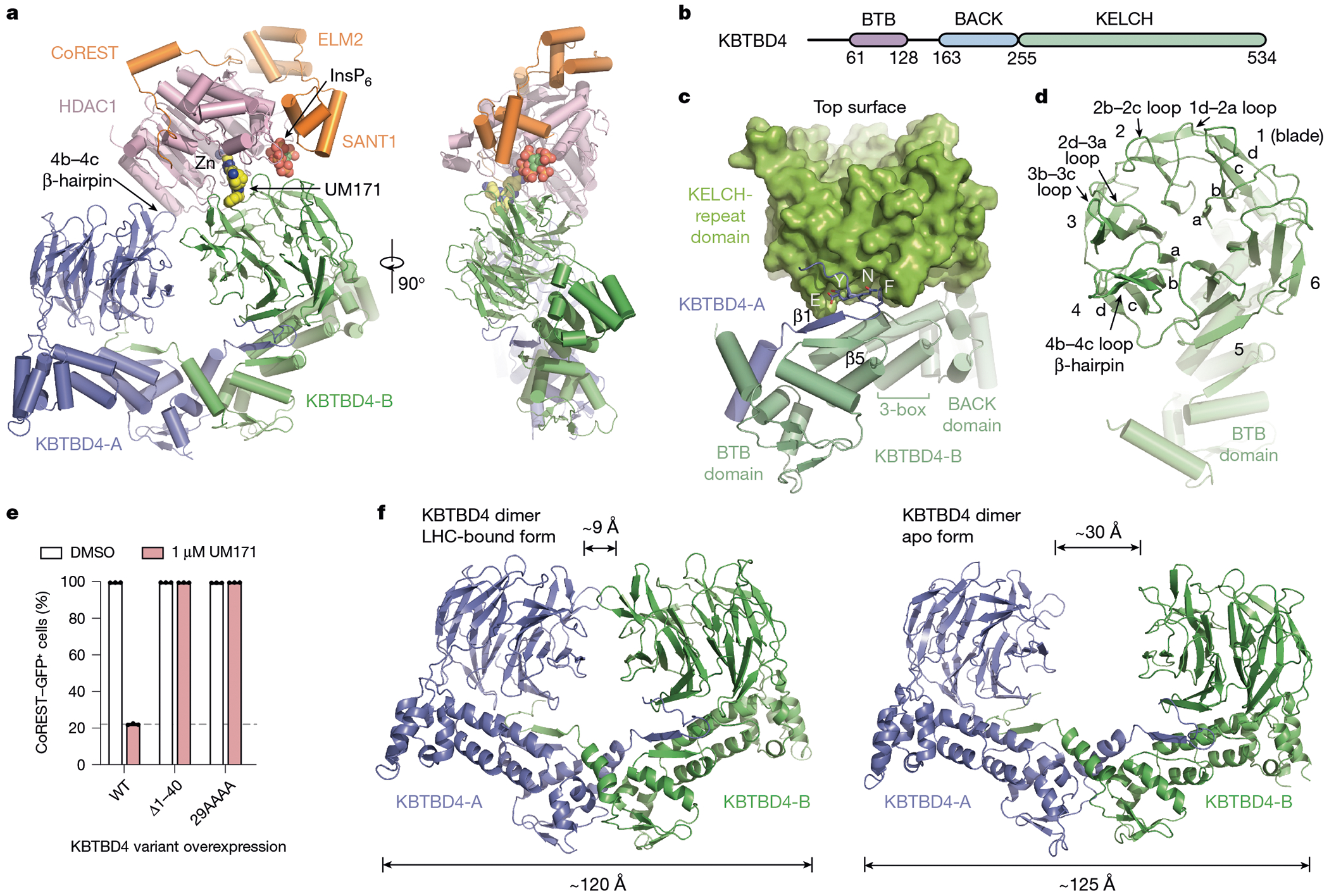
The overall structure of the KBTBD4–UM171–HDAC1–CoREST complex. **a**, Two orthogonal views of HDAC1 (pink) and CoREST-bound (orange) KBTBD4 (green/slate) with UM171 (space-filling model in yellow and blue) and InsP_6_ (space-filling model in green and red). **b**, Schematic of the protein domains of KBTBD4. **c**, The BTB and BACK domains as a cartoon representation and the KELCH-repeat domain as a surface representation of a KBTBD4 protomer (green) in the KBTBD4 dimer. The N-terminal region of the other protomer with the domain-swapped β1-strand flanked by the ENYF motif and an α-helix is shown in slate. Helices that are predicted to bind to CUL3 are indicated as 3-box. **d**, The KELCH-repeat β-propeller domain of KBTBD4 with its secondary structure elements annotated. **e**, Flow cytometry quantification of K562 *KBTBD4*-null CoREST–GFP cells overexpressing the indicated KBTBD4 variants after treatment with DMSO or UM171 for 24 h. Data are mean ± s.d. of *n* = 3 technical replicates and are representative of two independent experiments. **f**, The overall architectures of the LHC–UM171-bound and apo forms of the KBTBD4 homodimer. The closest distance between the two KELCH-repeat domains and the widest dimension of the E3 dimer in the two forms are indicated at the top and bottom of the dimers, respectively. FACS gating strategies are shown in Supplementary Fig. 1c.

Overall, the KBTBD4–UM171–LHC complex adopts an asymmetric architecture, in which two protomers of a KBTBD4 homodimer, hereafter referred to as KBTBD4-A and KBTBD4-B, simultaneously engage one molecule of HDAC1–CoREST in a bidentate manner (Fig. 3a). Although the SANT1 domain of CoREST is within close vicinity of KBTBD4, the closest Cα atoms between the two proteins remain 9 Å apart, and therefore complexation is exclusively driven by interactions between KBTBD4 and HDAC1. The two KBTBD4 protomers interact with HDAC1 through two distinct interfaces: (1) KBTBD4-A engages with the outer edge of the HDAC1 catalytic domain; whereas (2) KBTBD4-B cups HDAC1 at its active-site pocket.

A single molecule of UM171 is situated at the HDAC1–KBTBD4-B interface (Fig. 3a). By interacting with both HDAC1 and KBTBD4-B, UM171 fills an exposed gap between the two proteins with exquisite shape complementarity to act as a glue. Directly adjacent to the UM171 binding site, InsP_6_ is nestled at the three-protein junction between HDAC1, CoREST and KBTBD4-B to stabilize the complex as a second glue. Together, the E3 ligase dimer, the neosubstrate complex and the two small molecules bury a total surface area of around 2,300 Å^2^, with more than half of the interfaces contributed by protein–protein interactions.

## Structural plasticity of KBTBD4

**Extended Data Fig. 6 |:**
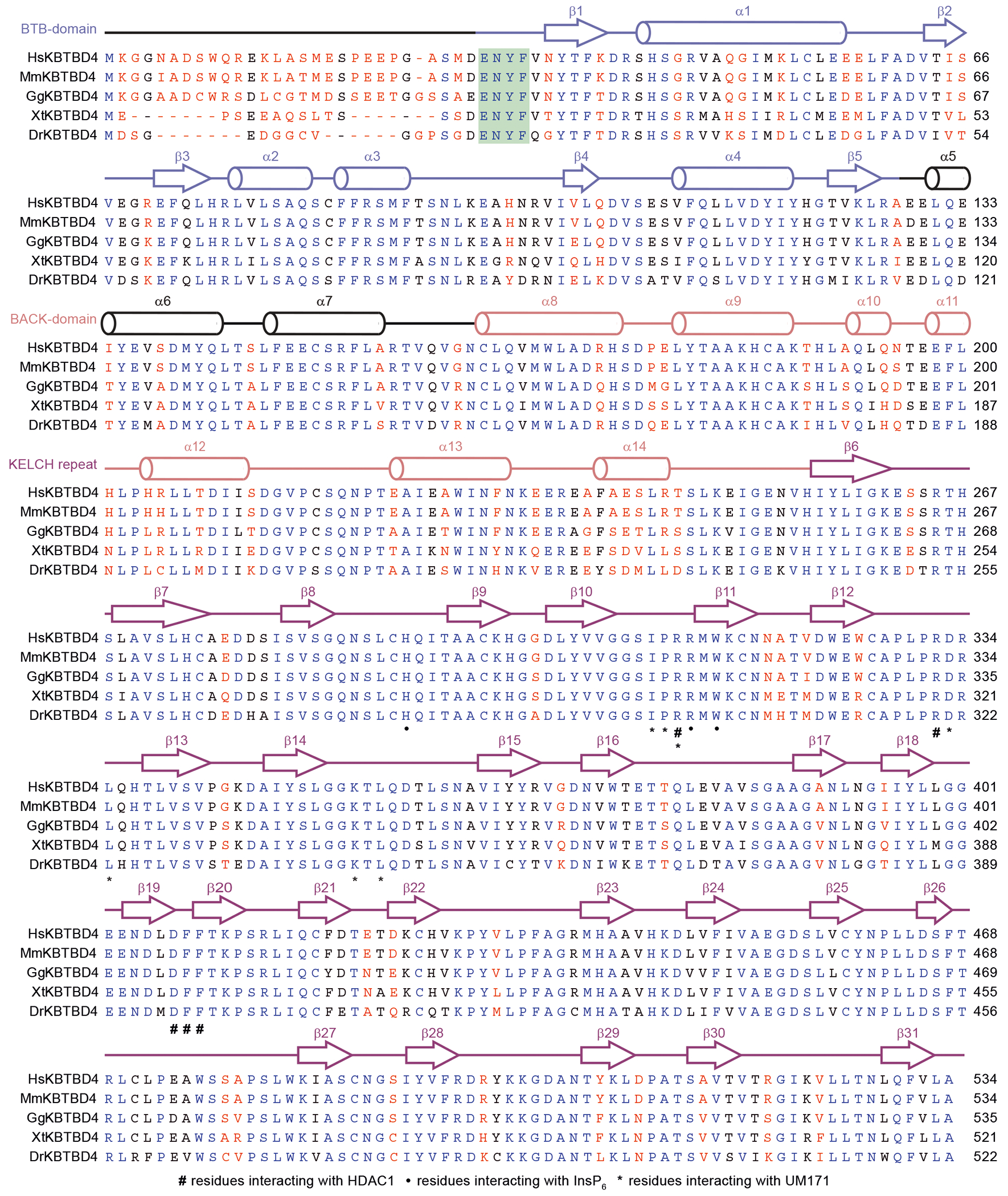
Sequence alignment and structural annotation of KBTBD4. Sequence alignment of five vertebrate KBTBD4 orthologues (Hs: *Homo sapiens*, Mm: *Mus musculus*, Gg: *Gallus gallus*, Xt: *Xenopus tropicalis*, Dr: *Danio rerio*) with secondary structure annotations. The sequences of the BTB, BACK and KELCH-repeat domains are underlined in different colours (slate, salmon, and purple). The residues interacting with HDAC1 are labelled with “#”; the residues interacting with InsP_6_ are labelled with “•”; and the residues interacting with UM171 are labelled with “*”. Strictly conserved residues are coloured in blue.

**Extended Data Fig. 7 |:**
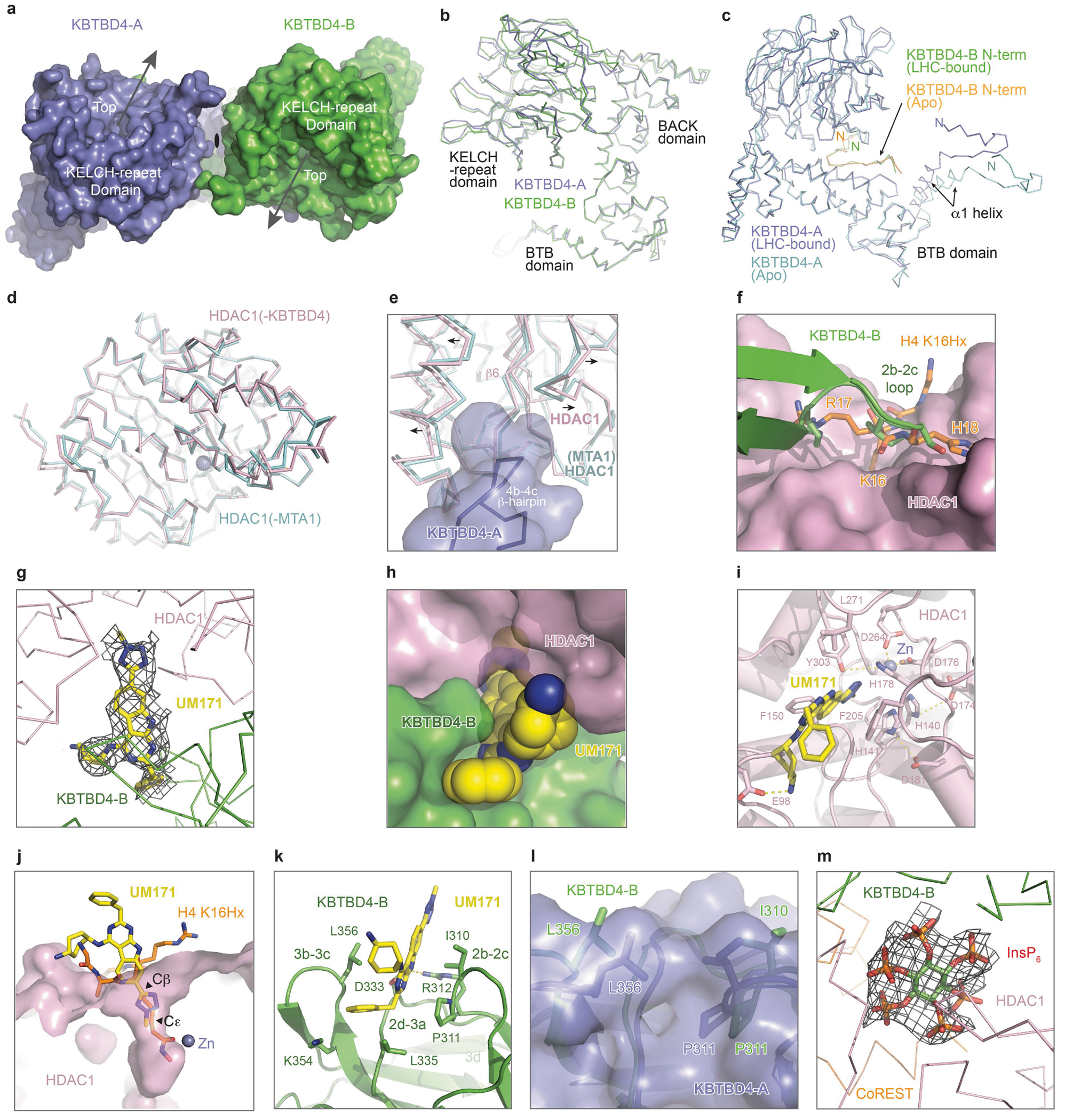
Structural analysis of the KBTBD4-LHC-UM171 complex. **a**, Top view of the KBTBD4 dimer with chain A coloured in slate and chain B coloured in green in pseudo-two-fold symmetry. **b**, Superposition of KBTBD4 chain A (slate) with chain B (green). **c**, Superposition of the protomers in the LHC-bound and the apo forms of the KBTBD4 dimer. **d**, Superposition of HDAC1 bound to KBTBD4 (pink) versus bound to MTA1 (cyan, PDB: 4BKX). **e**, Close-up view of the HDAC1 (pink) region remodelled by the 4b-4c β-hairpin (slate) of the KBTBD4-A (slate surface) versus HDAC1 (cyan) bound to MTA1 (PDB: 4BKX). **f**, Superposition comparison of the 2b-2c loop of KBTBD4-B (green) occupying the active-site of HDAC1 (pink) with the histone H4 K16Hx peptide (orange sticks) (PDB: 5ICN). **g**, Close-up view of the UM171 (yellow sticks) along with its density (dark grey mesh) at the interface between HDAC1 (pink) and KBTBD4-B (green). **h**, Close-up view of the surface complementarity among KBTBD4-B (green), HDAC1 (pink), and UM171 (yellow and blue spheres). **i**, Close-up view of UM171 (yellow and blue sticks) binding to the active-site pocket of HDAC1 (pink). Important residues demarcating the active-site of HDAC1 are shown in sticks with zinc (Zn) shown in slate sphere. Potential hydrogen bonds are shown in dashed lines. **j**, Superposition comparison of the HDAC1-binding modes between UM171 and the histone H4 K16Hx peptide (PDB:5ICN). **k**, Close-up view of UM171(yellow and blue sticks) binding to the surface of KBTBD4-B (green). The side chains of key UM171-contacting residues are shown in sticks. **l**, Superposition comparison of the UM171 binding region in KBTBD4-B (green) and the corresponding region in KBTBD4-A (slate surface). Residues involved in UM171 binding in KBTBD4-B and their corresponding residues in KBTBD4-A are highlighted in sticks. **m**, Close-up view of InsP_6_ (red, orange, and green sticks) with its density map (dark grey coloured mesh) at the tri-molecular junction among KBTBD4-B (green), HDAC1 (pink) and CoREST (orange).

KBTBD4 contains an N-terminal BTB domain, a central BACK domain and a C-terminal KELCH-repeat propeller domain (Fig. 3b and Extended Data Fig. 6). As expected, KBTBD4 forms a homodimer through its BTB domain, which is characterized by a domain-swapped two-stranded β-sheet (β1 and β5)^34^ (Fig. 3c). The predicted CUL3-binding 3-box helices of the KBTBD4 BTB domain are extended by five short helices in the BACK domain, which is connected to the KELCH-repeat domain through a linker sequence^12,35^. Together, the BTB-BACK modules of the two KBTBD4 protomers give rise to a V-shaped platform, which holds the two KELCH-repeat domains on the same side of the E3 homodimer without physically contacting one another. Overall, the KBTBD4 dimer has a pseudo-two-fold symmetry with its two protomers superimposable with a root mean squared deviation of 0.85 Å over 445 Cα atoms (Extended Data Fig. 7a, b).

The C-terminal KELCH-repeat domain adopts a canonical six-bladed propeller fold with each blade characterized by four β-strands conventionally named ‘a’ to ‘d’ (ref. 36) (Fig. 3d). Distinct from the other five blades, the fourth blade features an extended b–c loop, which protrudes from the top surface of the propeller. Although the central pocket presented by the top surface of a propeller fold is frequently used by KELCH-repeat-domain-containing E3s to engage their substrates^37–39^, the two propellers in the KBTBD4 dimer orient their top surfaces in opposite directions (Extended Data Fig. 7a) and instead use mostly their lateral surfaces and the b–c loops to recognize HDAC1.

Notably, the N-terminal β1 strand of the KBTBD4 BTB domain is led by a conserved ENYF motif (Extended Data Fig. 6), which packs against the C-terminal KELCH-repeat propeller of the second protomer to structurally couple the two halves of the CRL3 substrate receptor (Fig. 3c). Overexpression of KBTBD4 mutants lacking the N terminus (KBTBD4(Δ1–40)) or containing each residue of the ENYF motif mutated to alanine (KBTBD4(29AAAA)) in CoREST–GFP *KBTBD4*-null cells completely abrogated CoREST degradation by UM171 (Fig. 3e and Extended Data Fig. 2a), suggesting that proper positioning of the two KELCH-repeat domains against the BTB N-terminal α1 helices is critical for E3 function.

In comparison to the LHC-bound structure, the V-shaped platform of the free KBTBD4 dimer adopts a more open conformation (Fig. 3f). Superposition analysis shows that each protomer in the free KBTBD4 dimer is largely identical to that of the LHC-bound form (Extended Data Fig. 7c). However, the BTB N-terminal α1 helix in each protomer is tilted away from the BTB core, thereby flattening the V-shaped scaffold and further separating the two KELCH-repeat domains from one another (from around 9 Å to 30 Å). Thus, UM171-induced LHC binding involves significant conformational changes within the E3, which are accommodated by structural plasticity at the KBTBD4 dimer interface.

## HDAC1–KBTBD4-A interface

**Fig. 4 |:**
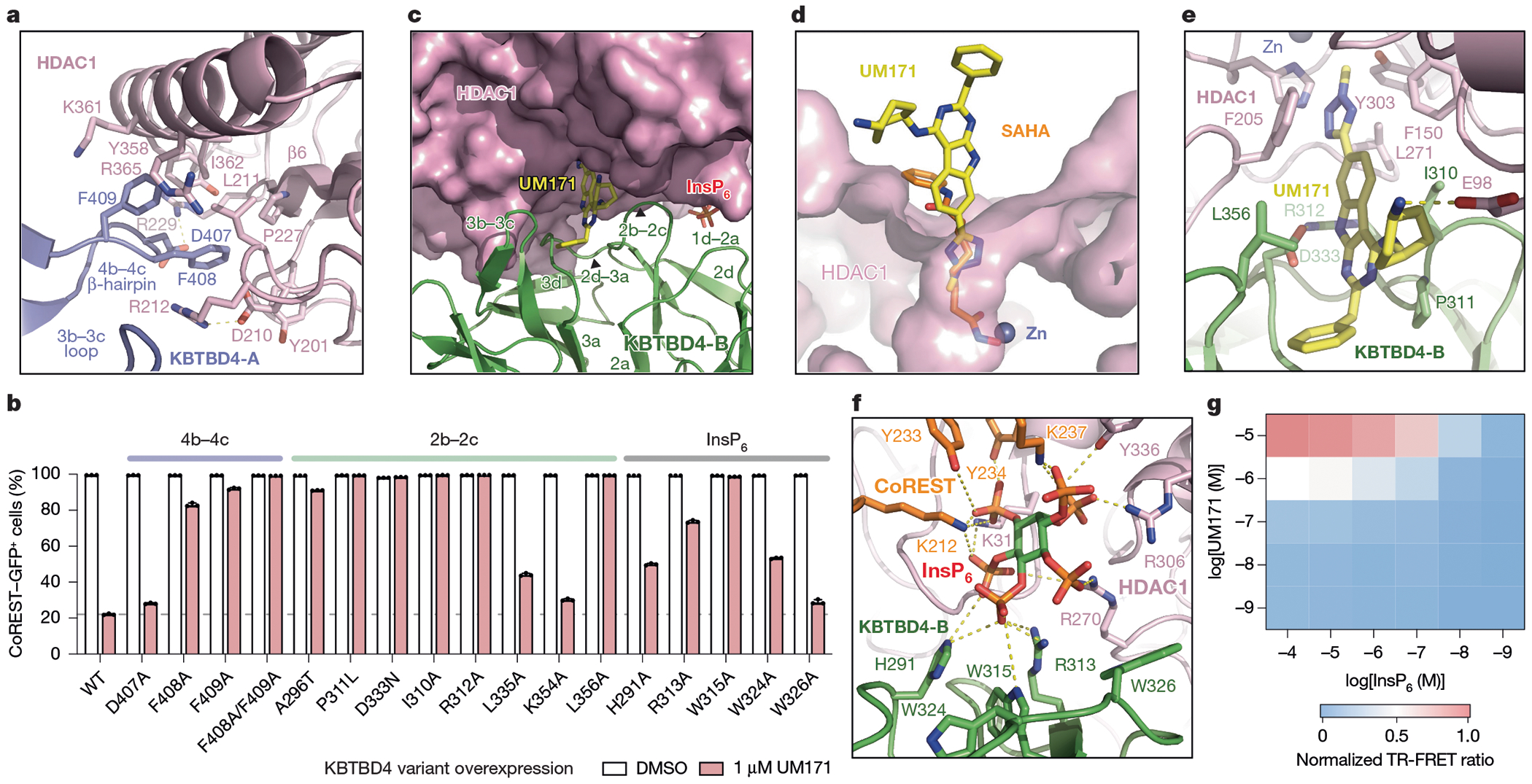
UM171 and InsP_6_ establish a bimolecular glue interface. **a**, The interface between the 4b–4c β-hairpin of KBTBD4-A (slate) and HDAC1 (pink). The side chains of the interacting amino acids are shown as sticks. **b**, Flow cytometry quantification of K562 *KBTBD4*-null CoREST–GFP cells overexpressing the indicated KBTBD4 variants after treatment with DMSO or UM171 for 24 h. Data are mean ± s.d. of *n* = 3 technical replicates. **c**, View of the interface formed between KBTBD4-B (green) and HDAC1 (pink), with UM171 shown as yellow and blue sticks and InsP_6_ shown as green, orange and red sticks. Secondary structures involved in protein–protein interactions are annotated. **d**, Comparison of UM171 (yellow and blue sticks) with SAHA (orange, blue and red sticks) binding to the active-site pocket of HDAC1 (pink surface), with zinc (Zn) shown as a slate sphere based on the HDAC2–SAHA structure (Protein Data Bank (PDB): 4LXZ) superimposed with HDAC1. **e**, Magnified view of UM171 (yellow and blue sticks) binding to the surface pocket formed between KBTBD4-B (green) and HDAC1 (pink). Side chains of select UM171-contacting residues are shown as sticks. **f**, Magnified view of the interactions made by InsP_6_ (red, orange and green sticks) to KBTBD4-B (green), HDAC1 (pink) and CoREST (orange); residues involved in the interactions are highlighted as sticks. Potential salt bridges and hydrogen bonds are shown as dashed lines. **g**, The normalized TR-FRET signal between fluorescein–LHC and anti-His CoraFluor-1-labelled antibody with His–KBTBD4 in the presence of varying concentrations of InsP_6_ and UM171. *n* = 2 biological replicates. For **b** and **g**, data are representative of two independent experiments. FACS gating schemes are shown in Supplementary Fig. 1c.

Consistent with previous studies, HDAC1 adopts a single α/β fold with a central eight-stranded parallel β-sheet sandwiched by α-helices on its two faces and possesses the characteristic catalytic zinc ion deep in its active site^40^. Superposition of the MTA1-bound and KBTBD4-bound HDAC1 structures shows that the deacetylase does not undergo major conformational changes after E3-UM171 binding, although an α-helix and a loop region concealing the C-terminal edge of HDAC1’s central β-sheet are slightly spread apart by KBTBD4-A to promote KBTBD4–UM171–LHC complex formation^41^ (Extended Data Fig. 7d,e). At this interface, the β-hairpin of the KBTBD4-A 4b–4c loop wedges into a hydrophobic cleft demarcated by the outer strand (β6) of HDAC1’s central β-sheet and its two surrounding secondary structure elements. Phe408 and Phe409 at the tip of the KBTBD4-A β-hairpin contact five hydrophobic residues in HDAC1 (Tyr201, Leu211, Pro227, Tyr358 and Ile362) (Fig. 4a). These interactions are reinforced by an intermolecular salt bridge between KBTBD4-A Asp407 and HDAC1 Arg229. Consistent with an important role in stabilizing the KBTBD4–UM171–LHC complex, mutation of KBTBD4 Phe408 and Phe409 to alanine blocked CoREST degradation by UM171 (Fig. 4b). By contrast, KBTBD4(D407A) had a lesser impact on CoREST degradation. Together, engagement by KBTBD4-A is critical for HDAC1 recognition and CoREST degradation.

## HDAC1–UM171–KBTBD4-B interface

In cooperation with UM171, the KELCH-repeat domain of KBTBD4-B forms an extensive interface with the substrate-binding site of HDAC1, encompassing the lateral and relatively flat surface of blade 2 and 3 of its propeller domain (Figs. 3a and 4c). Multiple solvent-exposed hydrophobic and polar residues in KBTBD4-B participate in engaging more than half of the active-site loops on one side of the deacetylase. In particular, the tip of the KBTBD4-B 2b–2c hairpin loop occupies the region of HDAC1 that recognizes its histone H4 substrate^41^ (Extended Data Fig. 7f). Despite their proximity, none of the KBTBD4-B residues are positioned close enough to access the HDAC1 active-site pocket, allowing UM171 to insert into and complement this interface (Extended Data Fig. 7g,h).

UM171 stabilizes the KBTBD4-B–HDAC1 interactions by bridging both proteins. On the HDAC1 side, UM171 inserts its *N*-methyl-tetrazole into the active-site pocket of the enzyme, contacting Phe150 and Phe205 (Fig. 4d,e and Extended Data Fig. 7i). The *N*-methyl group reaches as deep as Cε of the histone H4 Lys16 side chain (Extended Data Fig. 7j). The UM171 tricyclic pyrimidoindole core lies at the periphery of the pocket, with the cyclohexylamine extending outwards to form a salt bridge with Glu98 of HDAC1. Superposition analysis of UM171-bound HDAC1 and SAHA-bound HDAC2 shows that the corresponding small molecules would competitively occupy the active site, explaining their observed mutual exclusivity^42^ (Figs. 2d,e,h and 4d). However, whereas most HDAC1 active-site inhibitors (for example, SAHA) contact the catalytic zinc, UM171 does not. These findings support our observations that UM171 cannot directly bind to HDAC1/2–CoREST alone, instead requiring KBTBD4 for engagement (Extended Data Fig. 3e).

On the KBTBD4-B side, the pyrimidoindole core and the benzyl group of UM171 are embedded into a surface groove between the b–c loops of blade 2 and 3 (Extended Data Fig. 7k). The benzyl group of UM171 packs against Pro311, Leu335 and Lys354 while its pyrimidoindole ring is flanked by Ile310 and Leu356 and H-bonds with Asp333 (Fig. 4e, and Extended Data Fig. 7k). Consistent with these observations, mutation of Ile310, Pro311, Arg312, Asp333, or Leu356 inhibits CoREST–GFP degradation by UM171 (Fig. 4b). Ile310 and Leu356 of KBTBD4-B also make direct hydrophobic interactions with Phe150 and Phe205 of HDAC1, respectively (Fig. 4e). These four hydrophobic residues, together with Leu271 of HDAC1, surround UM171 and nucleate a hydrophobic core at the protein–protein interface. Notably, the surface groove of the E3 is closed on the KBTBD4-A propeller and incompatible with UM171 binding (Extended Data Fig. 7l). The b–c loops of blade 2 and 3 in KBTBD4-B are therefore most likely spread open by the small molecule with the support of HDAC1. These observations probably explain why UM171 does not show any detectable affinity towards the free KBTBD4 protein.

## InsP_6_ is a second molecular glue

The HDAC1–MTA1 complex is stabilized by inositol phosphates, which bind at the interface between HDAC1 and the corepressor^21,31^. In the KBTBD4–UM171–LHC complex, a clear density of InsP_6_ is present at the expected binding site between HDAC1 and CoREST (Figs. 3a and 4f and Extended Data Fig. 7m). Notably, the InsP_6_ molecule also directly contacts KBTBD4-B. Although we cannot definitively assign the carbon atoms of InsP_6_, all six phosphate groups interact with the protein subunits.

On the LHC side, five phosphate groups in InsP_6_ coordinate several positively charged and tyrosine residues in HDAC1 (Lys31, Arg270, Arg306 and Tyr336) as well as the CoREST SANT1 domain (Lys212, Tyr233, Tyr234 and Lys237). On the KBTBD4-B side, His291 and Arg 313 each form a salt bridge with one of the six phosphates, and mutation of either residue to alanine blocks CoREST degradation by UM171 (Fig. 4b,f). Moreover, InsP_6_ contacts Trp315, which belongs to a cluster of three tryptophan residues on the lateral surface of the KBTBD4-B propeller (Trp315, Trp324 and Trp326). Mutation of these tryptophan residues to alanine revealed that only Trp315 is essential for CoREST degradation by UM171 (Fig. 4b).

The binding mode of InsP_6_ in the KBTBD4–LHC complex suggests that the cofactor synergizes with UM171 to form the E3–neosubstrate complex. Consistent with this notion, UM171 is insufficient to promote KBTBD4–LHC complex formation in the absence of InsP_6_ and vice versa (Fig. 2f). A TR-FRET-based cross titration of UM171 and InsP_6_ measuring KBTBD4–LHC binding revealed that complex formation was only observed in the presence of both small molecules (Fig. 4g). Together, our results reveal the notable dependence of a quaternary complex on two small molecule glues in shaping an extensive, induced protein–protein interface.

## Base editor scanning of *HDAC1* and *KBTBD4*

**Extended Data Fig. 8 |:**
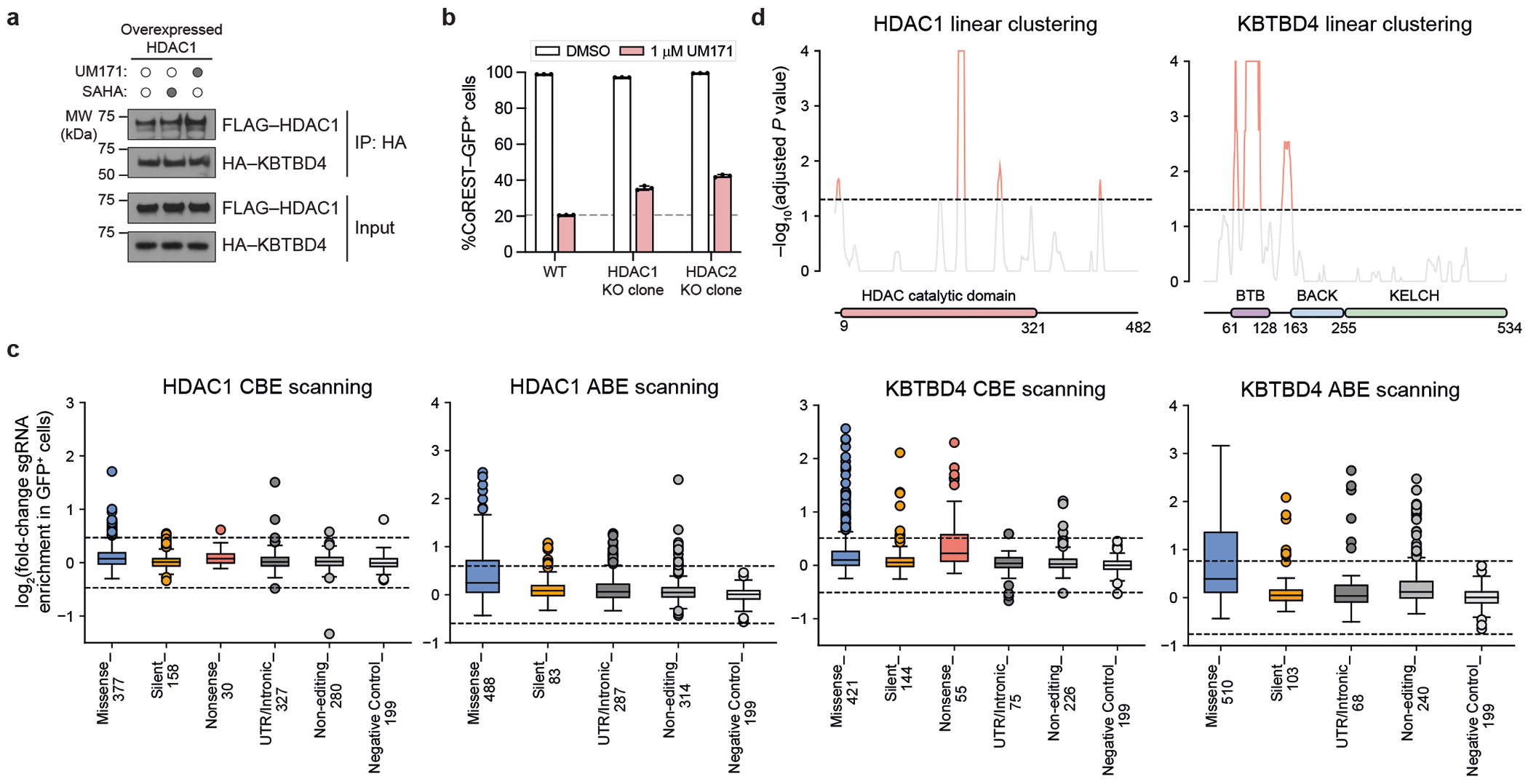
Base editor scanning of *HDAC1* and *KBTBD4*. **a**, Immunoblot of HA IP from 293T cells transfected with FLAG–HDAC1 and HA–KBTBD4 and treated with UM171 (1 μM), SAHA (10 μM), or DMSO for 1 h and MLN4924 (1 μM) for 3 h. **b**, Flow cytometry quantification in K562 *HDAC1*-null and *HDAC2*-null CoREST–GFP cells treated with DMSO (white) or 1 μM UM171 (pink) for 24 h. Bars represent mean ± s.d. of *n* = 3 biological replicates. **c**, Box plots of log_2_(fold-change sgRNA enrichment) for *HDAC1* and *KBTBD4* cytidine base editor (CBE) and adenosine base editor (ABE) scanning. sgRNAs classified by predicted editing outcome and the number of sgRNAs are indicated. Box plots show the median and interquartile range, with whiskers extending to 1.5× the interquartile range. Outliers are shown individually. Data are mean of *n* = 3 biological replicates. **d**, Line plots showing −log_10_(adjusted *P* values, *P*) for the observed per-residue sgRNA enrichment scores for (left) HDAC1 and (right) KBTBD4 coding sequences. The dotted line corresponds to *P* = 0.05 and residues with *P* ≤ 0.05 are highlighted in red. Data in **a** and **b** are representative of two independent experiments. FACS-gating schemes and uncropped blots are in Supplementary Fig. 1b, 7, respectively. KO, knock out; UTR, untranslated region; HA, hemagglutinin; IP, immunoprecipitation; MW, molecular weight.

**Fig. 5 |:**
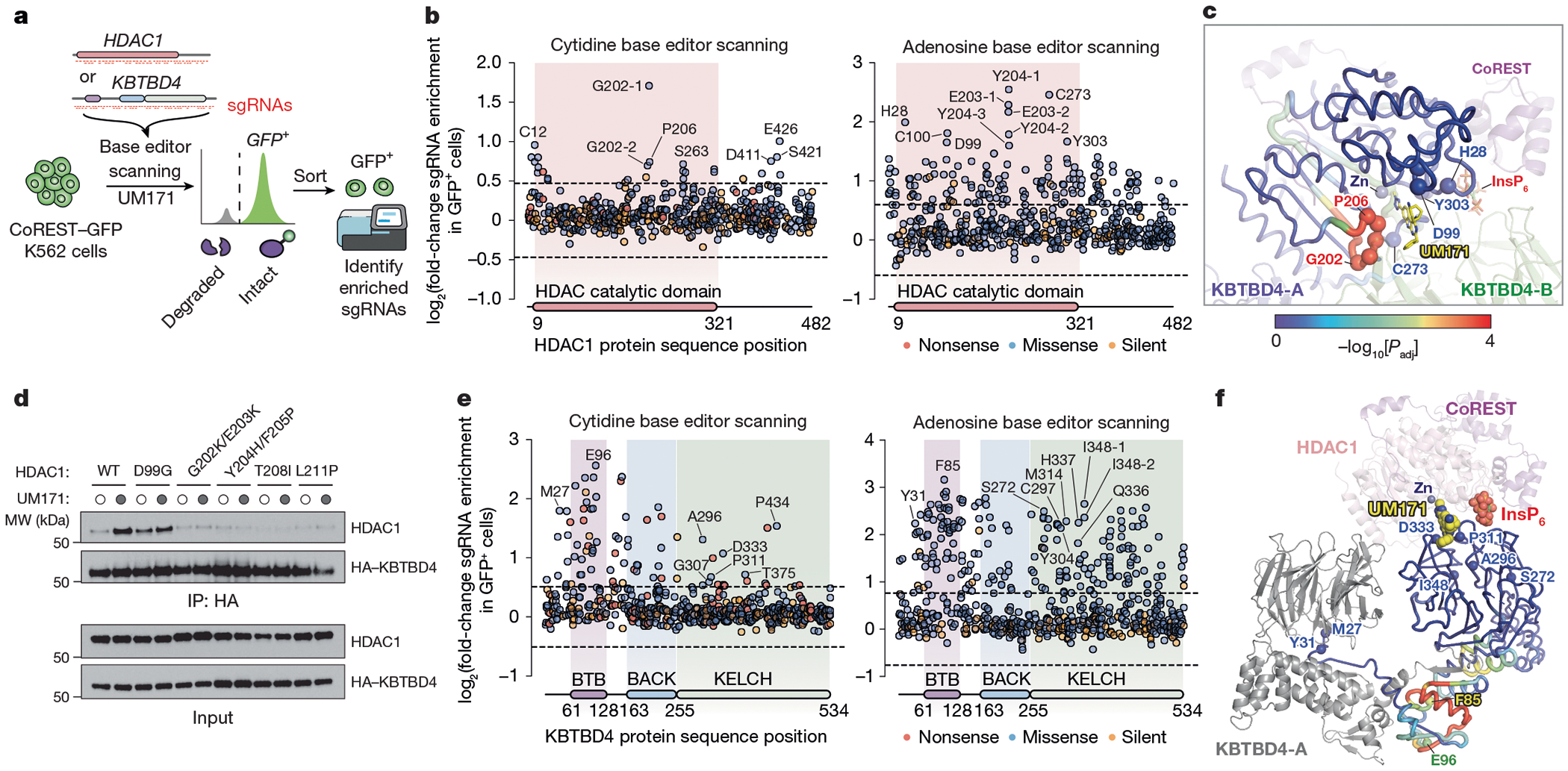
Base editing functionally maps the KBTBD4–UM171–HDAC1/2 interface. **a**, Schematic of base editor scanning of *HDAC1* (428 out of 482 residues; 88.6%) and *KBTBD4* (460 out of 534 residues; 86.1%) in K562 CoREST–GFP cells. The diagram was adapted from ref. 52. **b**, The log_2_[fold change in sgRNA enrichment] in GFP^+^ cells versus unsorted cells treated with 1 μM UM171 (*n* = 3) for 24 h for base editor scanning of *HDAC1* using a cytidine base editor (left) and adenosine base editor (right). The dotted lines represent ±4 s.d. from the mean of non-targeting controls (*n* = 199). Selected sgRNAs are labelled. **c**, The structure of HDAC1–CoREST–KBTBD4 showing HDAC1 residues coloured on the basis of linear clustering score from base editor scanning (Extended Data Fig. 8d). The Cα positions of selected top-enriched sgRNAs, marked in **b**, shown as spheres. *P*_adj_, adjusted *P*. **d**, Immunoblots of HA–KBTBD4 IP from clonal 293T cell lines containing the indicated HDAC1 base edits, transfected with HA–KBTBD4, and treated with DMSO or UM171 (1 μM) for 1 h and MLN4924 (1 μM) for 3 h. The base editing genotypes are shown in Extended Data Fig. 9a. Data are representative of two independent experiments. **e**, The log_2_[fold change in sgRNA enrichment] in GFP^+^ cells versus unsorted cells treated with 1 μM UM171 (*n* = 3) for 24 h for base editor scanning of *KBTBD4* using a cytidine base editor (left) and adenosine base editor (right). The dotted lines represent ±4 s.d. from the mean of non-targeting controls (*n* = 199). Selected sgRNA hit positions are labelled. **f**, The structure of HDAC1–CoREST–KBTBD4, showing KBTBD4-B residues coloured on the basis of the linear clustering score from base editor scanning (the same colour scale as in **c**; Extended Data Fig. 8d). The Cα positions of selected top-enriched sgRNAs, marked in **e**, are shown as spheres. FACS-gating schemes and uncropped blots are shown in Supplementary Figs. 1d and 4, respectively. HA, hemagglutinin; IP, immunoprecipitation; MW, molecular weight.

To test the interactions identified by cryo-EM, we sought to systematically mutate HDAC1 and KBTBD4 in cells and measure the subsequent impact on CoREST degradation by UM171. Overexpressed HDAC1 could not recapitulate ternary complex formation with UM171 and KBTBD4 (Extended Data Fig. 8a)–probably due to non-physiological expression that interferes with endogenous complex stoichiometry. Consequently, we used base editor scanning^43^ to systematically mutate endogenous HDAC1, using the expanded PAM variant SpG Cas9 cytidine and adenosine base editors (CBE and ABE, respectively) to increase amino acid mutational coverage^44^ (Fig. 5a and Supplementary Data 7–10). Owing to the redundancy of HDAC1 and HDAC2^26^, we generated CoREST–GFP knock-in cell lines containing *HDAC2* knockout to circumvent compensation during the base editor scanning (Extended Data Figs. 2e,f and 8b). Base editors and the pooled sgRNA library targeting *HDAC1* were introduced into K562 CoREST–GFP *HDAC2*-null cells, which were then treated with UM171 (24 h, 1 μM). Cells remaining GFP positive were sorted using fluorescence-activated cell sorting (FACS) and enriched sgRNAs were identified to reveal HDAC1 positions required for CoREST–GFP degradation (that is, positive sgRNA enrichment scores) (Fig. 5b and Extended Data Fig. 8c). Owing to the non-uniform coverage of sgRNAs, we used LOESS regression on a sliding window across the length of the protein to estimate per-residue enrichment scores from the measured sgRNA scores and then compared them to a null distribution generated by shuffling sgRNA scores. This enables us to determine whether a given stretch of residues may be more enriched than expected by chance^45^ (Methods and Extended Data Fig. 8d). Generally, this method assigns greater significance to short intervals along the linear coding sequence that contain multiple enriched sgRNA hits.

**Extended Data Fig. 9 |:**
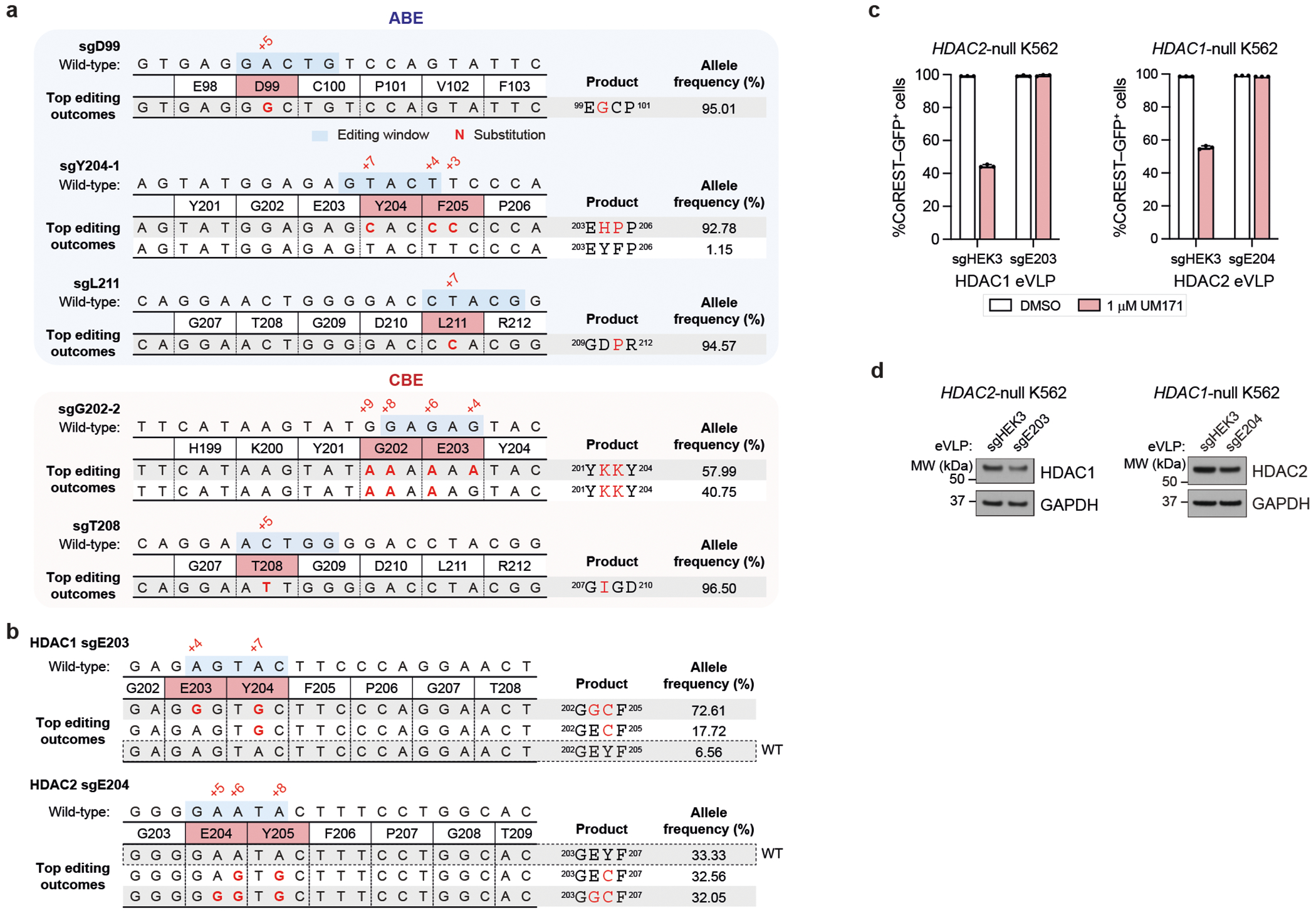
Genotyping and validation of *HDAC1/2* base edits by individual sgRNA transductions. **a**, Allele frequency analysis for 293T clonal cell lines after base editing of *HDAC1* with the indicated sgRNAs and SpG ABE8e or SpG CBE. Only alleles with ≥1% allele frequency in at least one sample are shown. Protein product sequences are shown with nonsynonymous mutations in red. Genotyping was performed once. **b**, Top: allele frequency analysis after base editing of *HDAC1* in K562 *HDAC2*-null cells with sgE203 and ABE8e. Bottom: allele frequency analysis after base editing of *HDAC2* in K562 *HDAC1*-null cells with sgE204 and ABE8e. Only alleles with ≥1% allele frequency in at least one sample are shown. Protein product sequences are shown with nonsynonymous mutations in red. Genotyping was performed once. **c**, Flow cytometry quantification showing GFP signal in indicated K562 CoREST–GFP cell lines transduced with the indicated sgRNAs and treated with DMSO or UM171 for 24 h. Data are mean ± s.d. of *n* = 3 biological replicates. **d**, Immunoblots showing protein levels of HDAC1 (left) or HDAC2 (right) and GAPDH in *HDAC2*-null and *HDAC1*-null K562 CoREST–GFP cells, respectively, after transduction with indicated eVLPs. Data in **c** and **d** are representative of two independent experiments. FACS-gating schemes and uncropped blots are in Supplementary Figs. 1b, 8, respectively. MW, molecular weight.

Mapping these per-residue significance values for HDAC1 onto our cryo-EM structure revealed a strong mutational hotspot surrounding the UM171-binding site at the HDAC1–KBTBD4-B interface (Fig. 5c). Many of the corresponding base edits were predicted to alter residues that directly contact UM171 as well as KBTBD4-B (for example, sgH28, sgD99, sgG202, sgE203, sgY204, sgP206, sgS263, sgC273, sgY303), suggesting that they disrupt multiple aspects of complex formation (Fig. 5b). We generated and genotyped clonal 293T cell lines in which HDAC1 was edited by sgD99, sgG202-2 or sgY204-1 to test the UM171-binding site as well as by sgT208 or sgL211 to validate the KBTBD4-A contact site (Extended Data Fig. 9a). We confirmed that these base edits introduced into HDAC1 were sufficient to fully block KBTBD4–HDAC1 co-IP promoted by UM171 (Fig. 5d)—except for D99G, which had only a partial effect. Lastly, we generated *HDAC2*-null HDAC1(E203G/Y204C) and *HDAC1*-null HDAC2(E204G/Y205C) CoREST–GFP cell lines by base editing with HDAC1 sgE203-1 and HDAC2 sgE204, respectively. UM171 treatment had no effect on CoREST–GFP levels in either of these cell lines, supporting that HDAC1 and HDAC2 are functionally redundant in the degrader mechanism (Extended Data Fig. 9b–d).

**Extended Data Fig. 10 |:**
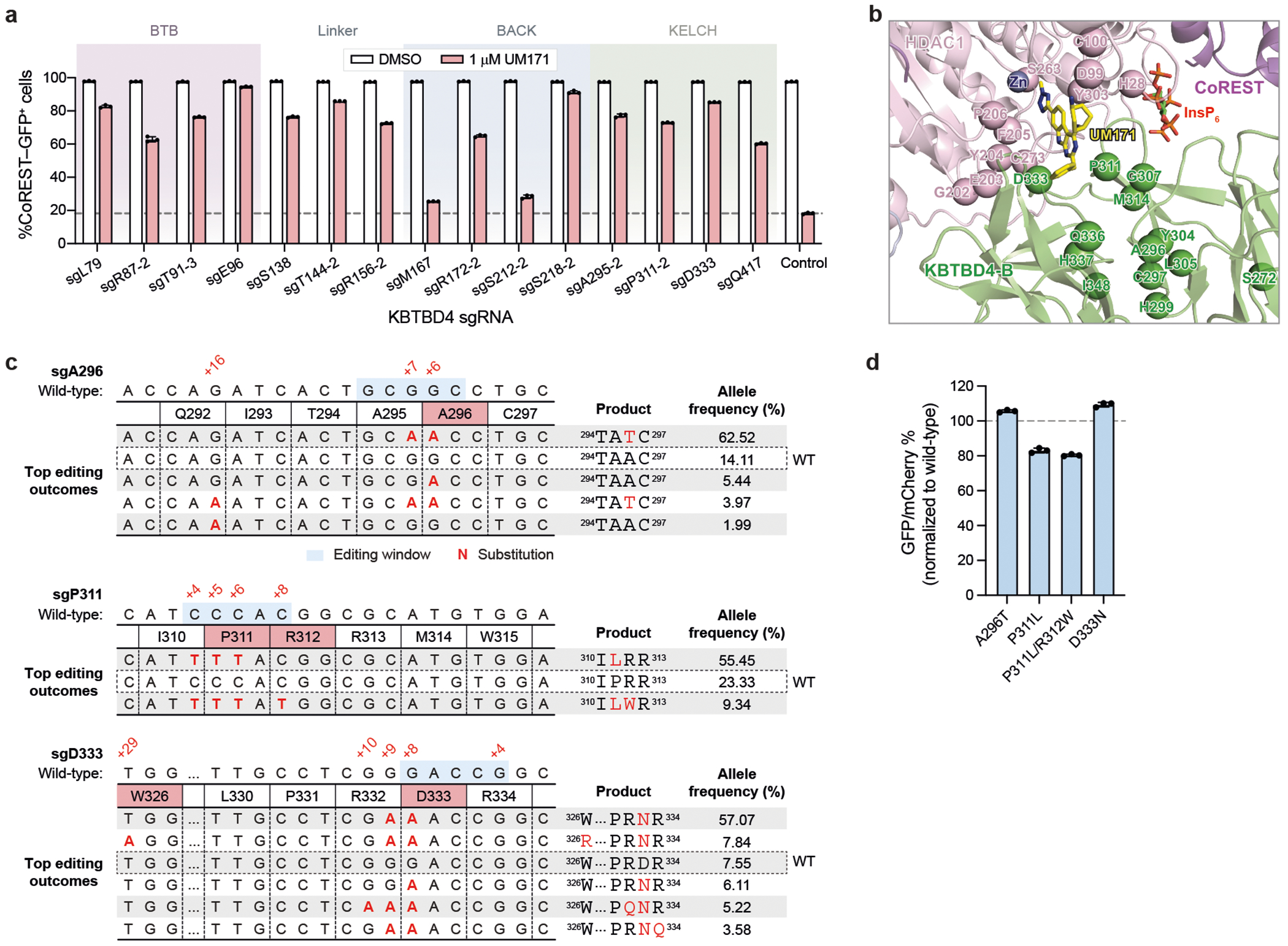
Validation and genotyping of KBTBD4 base edits. **a**, Flow cytometry quantification showing GFP signal in CoREST–GFP K562 cells transduced with the indicated KBTBD4 sgRNAs and treated with DMSO or UM171 for 24 h. Data are mean ± s.d. of *n* = 3 biological replicates and are representative of two independent experiments. **b**, Close-up view of the HDAC1-UM171-KBTBD4 interface showing Cα-positions of selected top-enriched sgRNAs, marked in Fig. 5b,e, as spheres. **c**, Base editing outcomes for selected KBTBD4 sgRNAs in K562 cells. The wild-type allele is boxed, and only alleles with ≥1% allele frequency in at least one sample are shown. Protein product sequences are shown with nonsynonymous mutations in red. Genotyping was performed once. **d**, Flow cytometry quantification of MOLM-13 cells expressing the indicated KBTBD4–GFP reporter. KBTBD4 stability calculated as GFP/mCherry and measurements are normalized to wild-type KBTBD4 analysed in parallel. Data are mean ± s.d. of *n* = 3 technical replicates and are representative of two independent experiments. FACS-gating schemes are in Supplementary Fig. 1b (a) and Supplementary Fig. 1a (d).

Using an analogous base editing strategy, we next identified functional residues on KBTBD4 (Fig. 5e; Supplementary Data 11–14). We validated that a subset of CBE base edits block CoREST–GFP degradation by individual sgRNA transduction (Extended Data Fig. 10a). As anticipated, many more sgRNAs targeting KBTBD4 scored as hits, as any significant loss-of-function mutation in the E3 can block degradation, in contrast to mutations in the neosubstrate (that is, HDAC1). Most top-enriched sgRNAs (CBE, 18 out of 80; ABE, 47 out of 197) targeted the BTB domain, and probably disrupt KBTBD4 homodimerization and/or interaction with CUL3 and, therefore, ligase activity. Consistent with this notion, linear clustering analysis of the KBTBD4 base editor scanning data showed that the strongest mutational hotspot resided in the BTB domain and along the dimerization interface (Fig. 5f and Extended Data Fig. 8d).

Many top-enriched sgRNAs are also predicted to alter various blades of the KELCH domain. Regions containing these individual top-enriched sgRNAs did not score highly in the linear clustering, probably due to the 3D structure of the β-propeller domain. In particular, several top-enriched base edits target regions surrounding the UM171-binding site between blade 2 and blade 3 (Fig. 5f and Extended Data Fig. 10b). We further investigated the base edits produced by sgA296, sgP311 and sgD333, first confirming the predicted base editing outcomes and protein stability of the corresponding KBTBD4 variants (Extended Data Fig. 10c,d). Overexpression of these KBTBD4 variants (that is, A296T, P311L, D333N) in *KBTBD4*-null CoREST–GFP cells showed that CoREST–GFP remained stable after UM171 treatment (Fig. 4b), confirming that these variants probably disrupt UM171 binding. Taken together, base editor scanning of the neosubstrate and E3 support, in the native context, the complex interfaces defined by our KBTBD4–UM171–LHC cryo-EM structure, showcasing the synergy of these approaches.

## Discussion

Here we elucidate the mechanism of action of UM171, establishing its target and function as a molecular glue. The cryo-EM structure of the KBTBD4–UM171–InsP_6_–LHC complex provides an example of a glue-licensed CRL3 E3 engaged with its neosubstrate. Among the superfamily of CRL E3s, CRL3 possesses the most substrate receptors, the majority of which share the same BTB-KELCH domain composition as KBTBD4^46–50^. Our results therefore substantially expand the repertoire of human E3 ligases that are potentially rewireable by glues. Importantly, in contrast to most CRL4s, CRL3s function as homodimers with two substrate-binding domains, enabling cooperative binding of two degrons encoded within a single substrate polypeptide. Rather than co-opting each substrate-binding domain individually, a single molecule of UM171 leverages both protomers of KBTBD4 to engage HDAC1 cooperatively and asymmetrically, underscoring the unique potential of dimeric E3s to be reprogrammed by small molecules.

Notably, UM171 binds to a pocket that is present only after KBTBD4-B–HDAC1 contact, suggesting that the surface of E3 scaffolds can be structurally plastic and might contain more binding sites for glue engagement than their apo structures reveal. A comparison between the free and LHC-bound forms of KBTBD4 further reveals an open-to-closed conformational change within the E3 homodimer, underscoring that the actions of glues can benefit from both local and global structural plasticity in E3s. Moreover, we demonstrate the requirement of InsP_6_ as a second glue at the KBTBD4–HDAC1–CoREST interface, inaugurating a dual-glue paradigm.

We establish HDAC1/2 as the target of UM171, highlighting modalities to target this enzyme class. Notably, UM171 does not only inhibit HDAC1/2 activity, but also leads to the potent degradation of selective subunits of HDAC1/2 complexes (that is, CoREST and MIER1), which are otherwise not readily amenable to therapeutic targeting. Finally, the mechanism of action of UM171 provides another example of how active-site ligands can serendipitously act as glues, further highlighting that enzyme active sites may be privileged as partners in pharmacologically induced protein–protein interactions^51^. In summary, this work reveals the considerable molecular sophistication of glue degraders in reprogramming extensive protein–protein contacts and vast opportunities for their prospective discovery.

## Online content

Any methods, additional references, Nature Portfolio reporting summaries, source data, extended data, supplementary information, acknowledgements, peer review information; details of author contributions and competing interests; and statements of data and code availability are available at https://doi.org/10.1038/s41586-024-08532-4.

## Methods

### Cell culture

MOLM-13 (ATCC) and SET-2 (DSMZ) cells were a gift from M. D. Shair. HEK293T cells (Thermo Fisher Scientific) were a gift from B. E. Bernstein. Gesicle Producer 293T cells were a gift from D. R. Liu (Takara, 632617). MV4;11 and K562 cells were obtained from ATCC. HEK293F cells were obtained from Thermo Fisher Scientific. All mammalian cell lines were cultured in a humidified 5% CO_2_ incubator at 37 °C and routinely tested for mycoplasma (Sigma-Aldrich). RPMI1640 and DMEM media were supplemented with 100 U ml^−1^ penicillin and 100 μg ml^−1^ streptomycin (Gibco) and FBS (Peak Serum). MOLM-13, MV4;11 and K562 cells were cultured in RPMI1640 (Gibco) supplemented with 10% FBS. SET-2 cells were cultured in RPMI1640 (Gibco) supplemented with 20% FBS. HEK293T and Gesicle Producer 293T cells were cultured in DMEM (Gibco) supplemented with 10% FBS. HEK293F cells were cultured in Freestyle 293 Expression Medium (Thermo Fisher Scientific) shaking at 125 rpm. *Spodoptera frugiperda* (Sf9) insect cells (Expression Systems, 94-001F) were cultured in ESF921 medium (Expression Systems) in a non-humidified and non-CO_2_ incubator at 27 °C shaking at 140 rpm. High Five and ExpiSf9 cells were purchased from Thermo Fisher Scientific (B85502 and A35243, respectively), with Grace insect medium (Thermo Fisher Scientific, 11595030) supplemented with 10% FBS (Cytiva) and 1% penicillin–streptomycin (Gibco), cultured at 26 °C. All of the cell lines were authenticated by short tandem repeat profiling (Genetica) and routinely tested for mycoplasma (Sigma-Aldrich).

### Lentiviral production

For lentivirus production, transfer plasmids were co-transfected with GAG/POL and VSVG plasmids into 293T cells using Lipofectamine 3000 (Thermo Fisher Scientific) according to the manufacturer’s protocol. Medium was exchanged after 6 h and the viral supernatant was collected 52 h after transfection and sterile-filtered (0.45 μm). MOLM-13 and K562 cells were transduced by spinfection at 1,800*g* for 1.5 h at 37 °C with 5 μg ml^−1^ and 8 μg ml^−1^ polybrene (Santa Cruz Biotechnology), respectively. Where necessary, 48 h after transduction, cells were selected with 1 μg ml^−1^ and 2 μg ml^−1^ puromycin (Thermo Fisher Scientific), respectively, for 3–5 days. For inducible expression experiments, K562 cells were selected with or 600 μg ml^−1^ geneticin (G418 sulfate) (Thermo Fisher Scientific) for 7–10 days.

### Plasmid construction

sgRNAs were ordered as synthetic oligonucleotides (Azenta/Genewiz), annealed and ligated into the appropriate vector: lentiCRISPR.v2 (Cas9 knockout), a gift from F. Zhang (Addgene, 52961); pRDA_478 (Addgene, 179096), which expresses BE3.9 (SpG), or pRDA_479 (Addgene, 179099), which expresses ABE8e (SpG) for base editing (gifts from J. Doench and D. Root). For individual sgRNA validation of the KBTBD4 CBE screen, sgRNAs were cloned into a pRDA_256 (Addgene, 158581) vector, a gift from J. Doench and D. Root, containing SpG Cas9 NG PAM. Other plasmids were cloned by Gibson Assembly using NEBuilder HiFi (New England Biolabs). Cloning strains used were NEB Stable (lentiviral) and NEB 5-alpha (other plasmids) (New England Biolabs). For base editor cloning, bacterial cultures were grown at 30 °C. Final constructs were validated by Sanger sequencing (Azenta/Genewiz).

All KBTBD4 expression plasmids encoded isoform 1 (human, residues 1–518) but longer isoform 2 (residues 1–534) numbering was used. CoREST expression plasmids encoded isoform 1 (human) in either full-length (considered residues 4–485) or various truncations. Open reading frames (ORFs) of human *KBTBD4* and *RCOR1* (mammalian expression) were obtained from Horizon Discovery. The full-length *MIER1* isoform 1 (human, residues 1–512) ORF was obtained from Gene-Copoeia and full-length RCOR2 isoform 1 (human, residues 1–523) was a gift from M. L. Suvà. The *LSD1* ORF was a gift from R. Shiekhattar. Full-length HDAC1 ORF was a gift from E. Verdin (Addgene, 13820). The coding sequence of HDAC2 (amino acids 2–488) was synthesized by IDT. The coding sequence of full-length *NUDCD3* (human, residues 1–361) was synthesized by Twist Biosciences.

For fluorescent and stability reporter constructs, CoREST, MIER1, RCOR2 and KBTBD4 were cloned into Cilantro 2, a gift from B. Ebert (Addgene, 74450). For transfection constructs, CoREST–FLAG and HA–KBTBD4 constructs were cloned into pcDNA3. For KBTBD4 overexpression constructs, *KBTBD4* coding sequences were cloned into pSMAL mCherry, which was generated from pSMAL through introduction of an mCherry ORF into pSMAL (a gift from J. E. Dick), or pFUGW-IRES-puro, which was generated from pFUGW (Addgene, 14883) by replacing the UbC promoter-eGFP cassette with an EFS-NS-IRES-puromycin cassette. For inducible expression constructs, *KBTBD4* coding sequence (CDS) was cloned into pInducer20, a gift from S. Elledge (Addgene, 44012). For bacmid expression, *KBTBD4* and *NUDCD3* were cloned into pFastbac, a gift from T. Cech. The KBTBD4 construct for structure determination was made by cloning human *KBTBD4* cDNA isoform 1 into a pFastBac vector with a tandem 10×His-tag and MBP tag at the N terminus followed by a TEV protease cutting site. For eVLP constructs, sgRNA sequences were cloned into pU6-sgRNA (a gift from D. R. Liu) by PCR amplification, and co-transfected with pCMV-MMLVgag-3×NES-ABE8e (Addgene, 181751), pBS-CMV-gagpol (Addgene, 35614) and pCMV-VSV-G (Addgene no. 8454), gifts from D. R. Liu, P. Salmon, and B. Weinberg, respectively.

### CRISPR–Cas9-mediated genome editing

#### Knock-in of CoREST–GFP in K562 cells.

mEGFP followed by a ‘GGGSGGGS’ linker was knocked into the C terminus of CoREST in K562 cells. sgRNA (sgRNA: TTCAAAGCCACCAGTTTCTC) targeting the C terminus of CoREST was cloned into a Cas9 plasmid, PX459^53^, and electroporated according to the manufacturer’s protocol (Neon Transfection System, Thermo Fisher Scientific) with a repair vector containing the mEGFP CDS and linker flanked by 750 bp of genomic homology sequences to either side of the CoREST C terminus. In brief, 2 × 10^5^ cells were washed twice with PBS and resuspended in buffer R. PX459 (0.5 μg) and the repair vector (0.5 μg) were added to the cell suspension, and electroporated at 1350 V with a 10 ms pulse width for 4 pulses using the Neon Transfection System 10 μl kit. After electroporation, cells were immediately transferred to prewarmed medium. To generate single-cell clones, cells were gated to sort for the top 0.2% GFP^+^ cells and single-cell sorted using the MoFlo Astrios EQ Cell Sorter (Beckman Coulter), expanded and validated by western blotting and Sanger sequencing.

#### Knock-in of HDAC2–dTAG in *HDAC1*-null CoREST–GFP K562 cells.

Homology-directed repair was used to insert a linker-FKBP12^F36V^-2xHA-P2A-Puro^R^ cassette into the C terminus of HDAC2 in *HDAC1*-null CoREST–GFP K562 cells (generation described below). sgRNA (sgRNA: GGTGAGACTGTCAAATTCAG) (Synthego) targeting the C terminus of HDAC2 was electroporated according to the manufacturer’s protocol (Neon Transfection System, Thermo Fisher Scientific) with a repair vector containing the linker-FKBP12^F36V^−2×HA-P2A-Puro^R^ CDS flanked by 700–800 bp of genomic homology sequences to either side of the HDAC2 C terminus. In brief, 2 × 10^6^ cells were washed twice with PBS and resuspended in buffer R. The sgRNA and the repair vector (0.5 μg) were added to the cell suspension, and electroporated at 1,350 V with a 10 ms pulse width for three pulses using the Neon Transfection System 100 μl kit. After electroporation, cells were immediately transferred to prewarmed medium. After 9 days of recovery, cells were selected with 2 μg ml^−1^ puromycin (Thermo Fisher Scientific) for 10 days before single-cell sorting on the MoFlo Astrios EQ Cell Sorter (Beckman Coulter). Single-cell clones were validated by Sanger sequencing and western blotting.

#### Generation of knockout K562 cells

Lentiviral vectors carrying sgRNA (*LSD1*, *HDAC1*, *HDAC2*) were generated by cloning appropriate sequences (*LSD1*: TAGGGCAAGCTACCTTGTTA; *HDAC1*: GCACCGGGCAACGTTACGAA; *HDAC2*: TACAACAGATCGT GTAATGA) into the pLentiCRISPR.v2 lentiviral vector. The control vector contained sgRNA targeting luciferase (sgControl). Lentivirus was produced and K562 CoREST–GFP cells were transduced and puromycin-selected as described above.

*HDAC1*-null, *HDAC2*-null and *KBTBD4*-null CoREST–GFP K562 clones were generated using the Alt-R CRISPR–Cas9 System (IDT) to deliver ribonucleoprotein complexes containing KO guides (*HDAC1*: GCACCGGGCAACGTTACGAA; *HDAC2*: TACAACAGATCGTGTAATGA; *KBTBD4*: GATATCTGTGAGTAAGCGGT) using the Neon Transfection System (Thermo Fisher Scientific) according to the manufacturer’s protocol. Transfected cells recovered for 72 h before sorting for single-cell clones on the MoFlo Astrios EQ Cell Sorter (Beckman Coulter). Single-cell clones were validated by genotyping and immunoblotting. sgRNA and primer sequences for validation are provided in Supplementary Tables 1 and 2, respectively.

### Proteomics sample preparation

MV4;11 and SET-2 (50 million cells per replicate) were treated with 1 μM UM171 or DMSO for 6 h. Cells were washed twice with ice-cold PBS and snap-frozen in liquid nitrogen for storage at −80 °C until use (*n* = 3, biological replicates). Frozen cell pellets were lysed in DPBS (Thermo Fisher Scientific) supplemented with benzonase (Santacruz Biotechnology) and protease inhibitor cocktail (Roche) using a chilled bath sonicator at 4 °C (Q700, QSonica). The lysates were clarified by centrifugation at 300*g* for 3 min. Proteins were quantified by BCA assay (Thermo Fisher Scientific) and normalized to 200 μg per 150 μl. Then, 200 μg of protein was reduced with 5 mM Tris(2-carboxyethyl) phosphine hydrochloride (TCEP) (Sigma-Aldrich) for 2 min and alkylated with 20 mM chloroacetamide (CAA) for 30 min at room temperature. Next, 1,000 μg of magnetic SP3 beads (1:1 hydrophobic:hydrophilic) (Cytiva) was added to each sample along with 100% liquid chromatography (LC)–MS-grade ethanol (Sigma-Aldrich) to reach the final concentration of 50% ethanol. The samples were then incubated for 30 min with KingFisher Flex system (Thermo Fisher Scientific) at room temperature. The beads were washed three times with 80% high-performance LC (HPLC)-grade ethanol (Sigma-Aldrich) and resuspended with 150 μl of trypsin/Lys-C (4 μg, Thermo Fisher Scientific) in 200 mM EPPS (pH 8.4)/5 mM CaCl_2_ (Sigma-Aldrich), and proteins were digested overnight for 16 h at 37 °C. Digested peptides were dried by a Speedvac, reconstituted with 5% acetonitrile (Sigma-Aldrich)/0.1% formic acid (Thermo Fisher Scientific) and desalted using Empore C18 Extraction Disks (3 M). Peptides were eluted with 80% acetonitrile/0.1% formic acid, dried by a Speedvac. Peptides reconstituted with 5% acetonitrile/0.1% formic acid were quantified using Quantitative Colorimetric Peptide Assay (Thermo Fisher Scientific) and 10 μg of peptides for each sample were labelled with 50 μg of TMTpro16-plex reagents (Thermo Fisher Scientific) per channel. TMT labelling was performed for 75 min with rotation at room temperature, and reaction was quenched by adding 5% hydroxylamine (Acros Organics) for 15 min, followed by addition of 10% formic acid. The samples were then pooled and dried using a Speedvac.

### High-pH reversed-phase peptide fractionation

Peptides were reconstituted with 300 μl of 5% acetonitrile/0.1% formic acid. Fractionation was performed using the Pierce High pH Reversed-Phase Peptide Fractionation Kit (Thermo Fisher Scientific) according to the manufacturer’s instruction. In brief, peptide samples were fractionated with 21 increments (7.5–55% with every 2.5% increase, and 75%) of acetonitrile with 10 mM NH_4_HCO_3_. Three eluents from every seventh fraction were pooled to get total seven fractions and dried using a Speedvac.

### MS data acquisition

Fractionated samples were reconstituted with 2% acetonitrile/0.1% formic acid and analysed on the EASY-nLC 1200 system (Thermo Fisher Scientific) coupled to the Orbitrap Eclipse Tribrid Mass Spectrometer (Thermo Fisher Scientific) with the FAIMSpro system equipped with real-time search function. Peptides were loaded onto a trap column (Pepmap 100 C18, 3 μm particle size, 100 Å pore size, 75 μm inner diameter × 150 mm length) and separated over a 140 min gradient of 5–35% acetonitrile in 0.1% formic acid and a flow rate of 300 nl min^−1^ with an analytical column (EASY-Spray C18 HPLC, 2 μm particle size, 75 μm inner diameter × 500 mm length). Peptides were acquired by data-dependent acquisition (DDA) and quantified using synchronous precursor selection MS3 (DDA-SPS-MS3); In brief, peptides were ionized at 2,300 V, separated by FAIMSpro (1.5 s per cycle) and scanned for MS1 analysis (resolution of 120,000; scan range of 400–1,400 *m*/*z*; maximum ion injection time (IIT) 50 ms; automatic gain control (AGC) setting of 10,000). MS2 analysis was collected from collision-induced dissociation (collision energy of 36%), and MS3 spectra were analysed in the orbitrap (resolution, 50,000; mass range, 100–500 Da).

### MS data analysis

Data processing was performed in ProteomeDiscoverer (PD) v.2.5 (Thermo Fisher Scientific) using the SequestHT algorithm. All raw files were submitted to search against the UniProtKB human universal database (UniProt: UP000005640, downloaded May 2020) combined with the common Repository of Adventitious Proteins (cRAP, classes 1, 2, 3 and 5) and the following parameters^54^; precursor tolerance of 10 ppm, fragment ion tolerance of 0.6 Da, minimum peptide length of 6 and trypsin full digestion with zero miscleavages. Cysteine carbamidomethylation (+57.021 Da) and methionine oxidation (+15.995 Da) were set as variable modifications while lysine- and N-terminus-TMTpro modification (+304.207 Da) were set as static modifications. Peptide-spectrum matches were filtered to a 1% false-discovery rate (FDR) using the Percolator algorithm (v.3.05.0) and further for protein assignment. Reporter ion quantifier node was set with the co-isolation threshold of 50, signal-to-noise threshold of 10 and SPS mass matches threshold of 50. Peptide abundance was normalized to total peptides. The protein ratio was calculated using the PD2.5 pairwise ratio-based algorithm and an empirical Bayes-moderated *t*-test was used to compare treatment groups using the limma R package (v.3.54.2)^55^. The R environment used was v.4.2.2. Data are provided in Supplementary Data 1 and 2. Volcano plots were created using the R package ggplot2 (v.3.5.1). Protein–protein interaction networks were constructed using STRINGdb (v.12)^56^, with a confidence threshold of >0.7, and the resulting networks were imported and visualized using Cytoscape (v.3.9.0).

### Immunoblotting

Cells were lysed on ice in RIPA buffer (Boston BioProducts) with 1× Halt Protease Inhibitor Cocktail (Thermo Fisher Scientific) and 5 mM EDTA (Thermo Fisher Scientific). The lysates were clarified by centrifugation and the total protein concentration was measured using the BCA Protein Assay (Thermo Fisher Scientific). The samples were electrophoresed and transferred to a 0.45 μm nitrocellulose membrane (Bio-Rad). The membranes were blocked with Tris-buffered saline Tween (TBST) with 5% blotting-grade blocker (Bio-Rad) and incubated with primary antibodies at the following dilutions: KBTBD4 (Novus Biologicals, NBP1-88587, 1:1,000), HDAC1 (Cell Signaling Technology, 34589, D5C6U, 1:1,000), HDAC2 (Cell Signaling Technology, 57156, D6S5P, 1:1,000), FLAG (Sigma-Aldrich, F1804, M2, 1:2,000), HA tag (Cell Signaling Technology, 3724, C29F4, 1:1,000), GAPDH (Santa Cruz Biotechnology, sc-47724, 0411, 1:10,000), H3K9ac (Abcam, AB32129, 1:2,000), H3 (Abcam, AB1791, 1:2,000). The membranes were washed three times with TBST and incubated with secondary antibodies at the following dilutions: anti-rabbit IgG HRP conjugate (Promega, W4011, 1:20,000), anti-mouse IgG HRP conjugate (Promega, W4021, 1:40,000) and goat anti-rabbit IgG HRP conjugate (Cell Signaling Technology, 7074, 1:2,000). After three washes with TBST, immunoblots were visualized using SuperSignal West Pico PLUS or SuperSignal West Femto chemiluminescent substrates (Thermo Fisher Scientific).

### Ubiquitin and serial proteome

#### Sample preparation.

SET-2 cells (10 million cells per replicate) were pretreated with 100 nM bortezomib or DMSO for 3 h and subsequently treated with 1 μM UM171 for 1.5 h or 6 h or DMSO for 6 h. Cells were washed twice with ice-cold PBS, and snap-frozen in liquid nitrogen for storage at −80 °C until use (*n* = 3, biological replicates). The samples underwent denaturing lysis in SDS to prepare for S-Trap digestion and lysed in 500 μl SDS lysis buffer (5% SDS, 50 mM TEAB pH 8.5, 2 mM MgCl_2_, 2 μg ml^−1^ aprotinin, 10 μg ml^−1^ leupeptin, 1 mM PMSF, 50 μM PR-619 (Lifesensors, SI9619: PR-619) and 1 mM chloroacetamide. The samples were disrupted by gentle vortexing and incubated at room temperature for about 15 min. The samples were treated with 3 μl 250 U μl^−1^ benzonase (Thomas Scientific, E1014-25KU) to shear DNA, mixed again and incubated at room temperature for another ~15 min. The lysates were cleared by centrifugation for 10 min at 20,000*g* and the supernatant was prepared for S-Trap digestion. The protein concentration was estimated using the BCA protein assay. Disulfide bonds were reduced in 5 mM DTT for 1 h at 25 °C and 1,000 rpm shaking, and cysteine residues were alkylated in 10 mM IAA in the dark for 45 min at 25 °C under 1,000 rpm shaking. Then, 12% phosphoric acid was added at a 1:10 ratio of lysate volume to acidify, and proteins were precipitated with 6× sample volume of ice-cold S-Trap buffer (90% methanol, 100 mM TEAB). The precipitate was transferred in successive loads of 3 ml to a S-Trap Midi (Protifi) and loaded with 1 min centrifugation at 4,000*g*, mixing the remaining precipitate thoroughly between transfers. The precipitated proteins were washed four times with 3 ml S-Trap buffer at 4,000*g* for 1 min. To digest the deposited protein material, 350 μl digestion buffer (50 mM TEAB) containing both trypsin and LysC, each at 1:50 enzyme:substrate weight:weight ratio, was passed through each S-Trap column with 1 min centrifugation at 4,000*g*. The digestion buffer was then added back atop the S-Trap and the cartridges were left capped overnight at 25 °C. Peptide digests were eluted from the S-Trap, first with 500 μl 50 mM TEAB and next with 500 μl 0.1% formic acid, each for 30 s at 1,000*g*. The final elution of 500 μl 50% acetonitrile/0.1% formic acid was centrifuged for 1 min at 4,000*g* to clear the cartridge. Eluates were frozen and dried in a vacuum centrifuge. Peptides were reconstituted in 30% acetonitrile/0.1% formic acid, and the concentration was estimated using the BCA assay.

#### Enrichment of K-ε-GG peptides.

Enrichment of K-ε-GG peptides was performed using the UbiFast method as previously described^57,58^. For each sample, 500 μg peptides was reconstituted in 250 μl HS bind buffer (Cell Signaling Technology) with 0.01% CHAPS. Reconstituted peptide was added to 5 μl PBS-washed HS anti-K-ε-GG antibody bead slurry (Cell Signaling Technology, 59322) in a 96-well KingFisher plate (Thermo Fisher Scientific). The plate was covered with foil and incubated for 1 h at 4 °C with end-over-end rotation. The plate containing peptides and anti-K-ε-GG antibody beads was then processed on the KingFisher Flex as previously described^57^. In brief, bead-bound enriched peptides were washed with 50% acetonitrile/50% HS wash buffer followed by awash in PBS. K-ε-GG peptides were labelled while on-bead with freshly prepared 400 μg TMTpro reagents (Thermo Fisher Scientific) in 100 mM HEPES for 20 min and labelling was quenched with 2% hydroxylamine. The beads were then washed with HS wash buffer before being deposited into 100 μl PBS. All sample wells were combined, the supernatant was removed and enriched K-ε-GG peptides were eluted from the beads with 2 × 10 min 0.15% TFA. The eluate was desalted using C18 StageTips, frozen and dried in a vacuum centrifuge.

#### TMT labelling of UbiFast flow-through for serial proteome.

Non-TMT-labelled K-ε-GG-enrichment flowthroughs were processed for proteome analysis as previously described^59^. In brief, peptides were acidified to 1% formic acid and desalted with 50 mg tC18 SepPak cartridges. The eluates were frozen and dried in a vacuum centrifuge. Peptides were reconstituted in 30% acetonitrile/0.1% formic acid, and the concentration was estimated using the BCA assay; 100 μg of each sample was reconstituted in 60 μl 50 mM HEPES and labelled with 200 μg TMT-Pro18 reagents at a final concentration of 20% acetonitrile for 1 h at 25 °C and 1,000 rpm. Labelling reactions were diluted to 5 mg ml^−1^ with 50 mM HEPES. Complete labelling and balancing of input material were confirmed. TMT labelling was quenched with 3 μl 5% hydroxylamine for 15 min and each TMTPro18 plex was combined, frozen and dried. Dried, labelled and combined peptides were reconstituted with 1 ml 1% formic acid and desalted with a 100 mg tC18 SepPak. The eluate was snap-frozen and dried in a vacuum centrifuge.

Offline bRP fractionation was performed to separate peptides over a 96 min gradient with a flow rate of 1 ml min^−1^. Solvent A was 5 mM ammonium formate/2% acetonitrile and solvent B was 5 mM ammonium formate/90% acetonitrile. In total, 96 fractions were concatenated into 24 fractions for proteome analysis. Then, 5 μg of peptides from each of the 24 fractions was transferred into HPLC vials, frozen and dried in a vacuum centrifuge for analysis. Proteome fractions were reconstituted in 3% acetonitrile/0.1% formic acid and 1 μg from each of the 24 fractions was injected for LC–MS/MS analysis.

#### LC–MS/MS for ubiquitin proteomics and serial proteome.

K-ε-GG peptides were reconstituted in 9 μl 3% acetonitrile/0.1% formic acid and 4 μl was injected twice onto a Orbitrap Exploris 480 mass spectrometer coupled to the Vanquish Neo UHPLC system (Thermo Fisher Scientific), equipped with FAIMS (Thermo Fisher Scientific) essentially as previously described^58^. The sample was injected onto a capillary column (Picofrit with 10 μm tip opening/75 μm diameter, New Objective, PF360-75-10-N-5) packed in-house with approximately 25 cm C18 silica material (1.5 μm ReproSil-Pur C18, Dr. Maisch) and heated to 50 °C. Peptides were separated at a flow rate of 200 nl min^−1^ with a linear 154 min gradient from 1.8% solvent B (acetonitrile, 0.1% formic acid), 2 min 5.4% B, 122 min 31.5% B, 130 min 54% B, 133 min 72% B, 144 min 45% B, 149 min 45% B. MS1 spectra were measured with a resolution of 60,000, an AGC target of 100% and a mass range from 350 to 1,800 *m*/*z*. Up to 10 MS2 spectra per duty cycle were triggered at a resolution of 45,000, an AGC target of 50%, an isolation window of 0.7 *m*/*z* and a normalized collision energy of 32. The FAIMS device was operated in standard resolution mode using the compensation voltages of −40, −60 and −80 for the first injection followed by a second injection with compensation voltages of −45, −50 and −70.

Proteome fractions were reconstituted in 3% acetonitrile/0.1% formic acid, and 1 μg from each of the 24 fractions was injected for LC–MS/MS analysis onto an Orbitrap Exploris 480 mass spectrometer coupled to a Vanquish Neo UHPLC system (Thermo Fisher Scientific) essentially as previously described^58^. The sample was injected onto a capillary column (Picofrit with 10 μm tip opening/75 μm diameter, New Objective, PF360-75-10-N-5) packed in-house with approximately 30 cm C18 silica material (1.5 μm ReproSil-Pur C18, Dr. Maisch) and heated to 50 °C. Peptides were eluted into the Orbitrap Exploris 480 at a flow rate of 200 nl min^−1^. The bRP fractions were run on a 110 min method, including a linear 84 min gradient from 94.6% solvent A (0.1% formic acid) to 27% solvent B (99.9% acetonitrile, 0.1% formic acid), followed by a linear 9 min gradient from 27% solvent B to 54% solvent B. MS was conducted using a data-dependent acquisition mode, where MS1 spectra were measured with a resolution of 60,000, a normalized AGC target of 300% and a mass range from 350 to 1,800 *m*/*z*. MS2 spectra were acquired for the top 20 most abundant ions per cycle at a resolution of 45,000, an AGC target of 30%, an isolation window of 0.7 *m*/*z* and a normalized collision energy of 34. The dynamic exclusion time was set to 20 s, and the peptide match and isotope exclusion functions were enabled.

#### Data analysis for ubiquitin proteomics and serial proteome.

MS data were processed using Spectrum Mill Rev BI.07.11.216 (https://proteomics.broadinstitute.org). Extraction of raw files retained spectra within a precursor mass range of 600 to 6,000 Da and a minimum MS1 signal-to-noise ratio of 25. MS1 spectra within a retention time range of ±45 s, or within a precursor *m*/*z* tolerance of ±1.4 *m*/*z*, were merged. MS/MS searching was performed against a human UniProt database. Digestion parameters were set to ‘trypsin allow P’ with an allowance of 4 missed cleavages. The K-ε-GG MS/MS search included fixed modifications, carbamidomethylation on cysteine and TMTPro on the N terminus and internal lysine, and variable modifications, acetylation of the protein N terminus, oxidation of methionine and K-ε-GG on tryptic peptide—‘Ubiquitin Residual GG from Tryp Cut on K’. The proteome MS/MS search included fixed modifications, carbamidomethylation on cysteine and TMTPro on the N terminus and internal lysine, and variable modifications, acetylation of the protein N terminus, oxidation of methionine, N-term deamidation, and N-term Q-pyroglutamate formation. Restrictions for matching included a minimum matched peak intensity of 40% for K-ε-GlyGly and 30% for proteome, and a precursor and product mass tolerance of ±20 ppm. Peptide-spectrum matches were validated using a maximum FDR threshold of 1.2% for precursor charge range to 2 to 6. A target protein score of 0 was applied during protein polishing autovalidation for the proteome to further filter peptide-spectrum matches. TMTpro reporter ion intensities were corrected for isotopic impurities using the afRICA correction method in the Spectrum Mill protein/peptide summary module, which uses determinant calculations according to Cramer’s rule. Protein quantification and statistical analysis were performed using the Proteomics Toolset for Integrative Data Analysis (Protigy, v.1.0.7, Broad Institute, https://github.com/broadinstitute/protigy). Each K-ε-GG peptide or protein was associated with a log_2_-transformed expression ratio for every sample condition over the median of all sample conditions. Median normalization was conducted separately on the K-ε-GG peptide data and the global proteome data. K-ε-GG peptide data were then normalized to the global proteome data using the panoply_ptm_normalization module of PANOPLY (PANOPLY, Broad Institute, https://github.com/broadinstitute/PANOPLY/wiki). Specifically, it takes all K-ε-GG peptide log-ratios in all samples and regresses them against the log-ratios of cognate proteins. Then, the resulting residuals are the normalized K-ε-GG peptide values. After normalization, an empirical Bayes-moderated *t*-test was used to compare treatment groups, using the limma R package^55^. *P* values associated with every modified peptide or protein were adjusted using the Benjamini–Hochberg FDR approach. Data are provided in Supplementary Data 4–6.

### Fluorescence degradation reporter assay

CoREST (full-length and truncated), MIER1 and RCOR2 inserts were PCR-amplified with Esp3I sites and ligated into a Cilantro 2 eGFP-IRES-mCherry reporter vector by golden-gate assembly. Point mutations were introduced into coding regions using standard PCR-based site-directed mutagenesis techniques. Deletion constructs were made by PCR amplification of the appropriate regions and cloned into the Cilantro 2 vector using Gibson cloning (New England Biolabs). Lentiviral particles carrying the respective constructs in the Cilantro 2 vector were produced and used to transduce MOLM-13 cells as described above. Then, 48 h after transduction, cells were selected with 1 μg ml^−1^ puromycin for 3–5 days. The selected cells were then treated with various concentrations of UM171 or 0.1% DMSO for 6 or 24 h. GFP and mCherry fluorescence were measured on a NovoCyte 3000RYB flow cytometer (Agilent) after drug or DMSO treatment. The geometric mean of the ratio of GFP to mCherry fluorescence was calculated for each sample using the NovoExpress software (v.1.5.0, Agilent). The ratios for the individual drug-treated samples were normalized to the ratios of the DMSO-treated samples in Microsoft Excel (v.16.80) and plotted using GraphPad Prism (v.9.4.0). All degradation assays were done in triplicate and FACS-gating schemes are shown in Supplementary Fig. 1a.

### Co-IP analysis

#### In K562 cells.

FLAG–KBTBD4 was cloned into pFUGW-IRES-puro and stably expressed in CoREST–GFP K562 cells by lentiviral transduction followed by puromycin selection, as described above. Cells were pretreated with either 10 μM SAHA (1 h) or DMSO, then treated with 1 μM MLN4924 for 3 h then 5 μM UM171 or DMSO for 1 h. Cells were washed twice with cold PBS and flash-frozen. Co-IP was performed as described below.

#### In HEK293T cells.

HEK293T cells were transfected with 3 μg pcDNA3.1 HA–KBTBD4 plasmid and 3 μg pcDNA3 CoREST–FLAG (full-length or truncated) using PEI MAX transfection reagent (Polysciences) according to the manufacturer’s protocol. Then, 48 h after transfection, cells were treated with 1 μM MLN4924 for 3 h then 1 μM UM171 or DMSO for 1 h. Cells were washed twice with cold PBS and flash-frozen. Co-IP was performed as described below.

Cells were thawed, lysed on ice in lysis buffer (25 mM Tris-HCl pH 7.5, 150 mM NaCl, 1% NP-40 alternative) supplemented with cOmplete, EDTA-free protease inhibitor cocktail (Sigma-Aldrich) and the lysates were cleared. The protein concentration was quantified as described above and diluted to 1 mg ml^−1^ in lysis buffer with 1 μM UM171 or DMSO. The supernatants were immunoprecipitated overnight at 4 °C with 25 μl Pierce anti-HA magnetic beads (Thermo Fisher Scientific). The beads were washed six times with lysis buffer, eluted in SDS–PAGE loading buffer and carried forward to immunoblotting as described above.

### Protein expression and purifications

Recombinant human KBTBD4 for biochemical and biophysical analyses was purified from Sf9 insect cells. cDNAs for human KBTBD4 and NUDCD3 proteins were cloned into the pFastBac donor vector and the recombinant baculoviruses were constructed using the Bac-to-Bac protocol and reagents (Thermo Fisher Scientific). KBTBD4 constructs were tagged on the N terminus with 6×His cleavable by TEV protease. These plasmids were used to prepare separate baculoviruses according to standard protocols (Bac-to-Bac Baculovirus Expression System, Thermo Fisher Scientific). Detection of gp64 was used to determine the baculovirus titre (Expression Systems). For expression, Sf9 cells were grown to a density of 1–2 × 10^6^ cells per ml and co-infected with NUDCD3 baculovirus at a multiplicity of infection (MOI) of 2 and KBTBD4 baculovirus at a MOI of 3.5. The cells were incubated for 72 h (27 °C, 120*g*), collected and then frozen with liquid nitrogen for future purification. Cells were resuspended in lysis buffer (50 mM Tris-HCl, pH 8.0 cold, 500 mM NaCl, 1 mM TCEP, 10% glycerol, 15 mM imidazole) supplemented with 1% NP-40, 1 mM PMSF and cOmplete, EDTA-free protease inhibitor cocktail (Sigma-Aldrich) and sonicated. The lysates were clarified by centrifugation at 100,000*g* for 30 min and incubated with His60 Ni Superflow affinity resin (Takara). Resin was washed with lysis buffer containing a stepwise gradient of 15–50 mM imidazole, followed by elution using lysis buffer with 250 mM imidazole. The eluate was exchanged into storage buffer (50 mM Tris-HCl, pH 8.0 cold, 150 mM NaCl, 1 mM TCEP, 10% glycerol) using an Econo-Pac 10DG desalting column (Bio-Rad) and further purified by size-exclusion chromatography using the Superdex 200 10/300 GL column (GE Healthcare). The purity of the recombinant protein was verified by SDS–PAGE and fractions with 90–95% purity were pooled and stored at −80 °C.

Recombinant human KBTBD4 used in cryo-EM structure determination was purified from *Trichoplusia ni* High Five insect cells. cDNAs for human KBTBD4 and NUDCD3 proteins were cloned into the pFastBac donor vector and the recombinant baculoviruses were constructed using the Bac-to-Bac protocol and reagents (Thermo Fisher Scientific). KBTBD4 constructs were tagged on the N terminus with 10×His and MBP tag cleavable by TEV protease. These plasmids were used to prepare separate baculoviruses according to standard protocols (Bac-to-Bac Baculovirus Expression System, Thermo Fisher Scientific). For expression, the monolayer High Five cells were grown to about 80% confluency and co-infected with NUDCD3 baculovirus. The cells were incubated for 72 h (26 °C), collected and then frozen with liquid nitrogen for future purification. Cells were resuspended in lysis buffer (50 mM Tris-HCl, pH 8.0 cold, 150 mM NaCl, 1 mM TCEP) supplemented with 1 mM PMSF, 10 μM leupeptin, 0.5 μM aproptinin and 1 μM pepstatin A and sonicated. The lysates were clarified by centrifugation at 100,000*g* for 30 min and incubated with amylose affinity resin (New England BioLabs). Resin was washed with lysis buffer, followed by elution using lysis buffer with 10 mM maltose. The eluate was cut with tobacco etch virus protease overnight, followed by the prepacked anion-exchange column (GE Healthcare) to get rid of the protease and further purified by size-exclusion chromatography using the Superdex 200 10/300 GL column (GE Healthcare). The purity of the recombinant protein was verified by SDS–PAGE and fractions with 90–95% purity were pooled and stored at −80 °C.

Recombinant HDAC1–CoREST comprised full-length HDAC1 (UniProt: Q13547) and CoREST (amino acids 86–485). HDAC2–CoREST complex comprising HDAC2 (amino acids 2–488) (UniProt: Q92769) and CoREST (amino acids 86–485) was purified from ExpiSf9 cells (Thermo Fisher Scientific). cDNAs for human HDAC1, HDAC2 and CoREST proteins were cloned into the pFastBac donor vector and the recombinant baculoviruses were constructed using the Bac-to-Bac protocol and reagents (Thermo Fisher Scientific). The HDAC1 construct was tagged on the C terminus with a FLAG tag, the HDAC2 (amino acids 2–488) construct was tagged on the N terminus with a SUMO tag, which can be cleaved in insect cells and with 6×His on the C terminus. CoREST (86–485) was tagged with a 10× His tag followed by an MBP tag on the N terminus. To improve the solubility of CoREST, six amino acids were mutated to the corresponding residues found in MIER2 (W172K F188C F191E V197A V201N F209K). These plasmids were used to prepare separate baculoviruses according to standard protocols (Bac-to-Bac Baculovirus Expression System, Thermo Fisher Scientific). The suspension ExpiSf9 cells were grown to about 5 × 10^6^ cells per ml before protein expression. For the HDAC1/2–CoREST complex or HDAC1/2 alone expression, the ExpiSf9 cells were either co-infected with HDAC1 or HDAC2 and CoREST baculovirus, or infected with HDAC1/2 baculovirus alone. The cells were incubated for 72 h (26 °C), collected and then frozen with liquid nitrogen for future purification. Cells were resuspended in lysis buffer (50 mM Tris-HCl, pH 8.0 cold, 300 mM NaCl, 5 mM MgCl, 15% glycerol, 1 mM TCEP, 20 mM imidazole) supplemented with 1 mM PMSF, 10 μM leupeptin, 0.5 μM aproptinin and 1 μM pepstatin A and sonicated. Lysate was clarified by centrifugation at 100,000*g* for 30 min and incubated with nickel affinity resin (Thermo Fisher Scientific) or FLAG resin (anti-FLAG M2 affinity gel, Sigma-Aldrich). Resin was washed with lysis buffer, followed by elution using lysis buffer with 200 mM imidazole or 200 μg ml^−1^ FLAG peptide. Eluate was applied to the prepacked anion exchange column (GE Healthcare) to get rid of the contaminants and further purified by size-exclusion chromatography using a Superdex 200 10/300 GL column (GE Healthcare). The purity of the recombinant protein was verified by SDS–PAGE and fractions with 90–95% purity were pooled and stored at −80 °C.

Recombinant LSD1–CoREST complex comprised LSD1 amino acids 151–852 and CoREST amino acids 308–485. LSD1 amino acids 151–852 were cloned into a pET15b vector (gift from P. A. Cole) containing an N-terminal 6×His-tag using NEBuilder HiFi DNA Assembly Master Mix (NEB, E2621L). The LSD1 constructs were expressed in BL21-CodonPlus (DE3)-RIPL competent *Escherichia coli* and after plating a single colony was cultivated in 2× YT with 100 mg l^−1^ ampicillin at 37 °C and expression was induced at an optical density of 600 nm (OD_600_) of 1.0 by adding 0.3 mM isopropyl β-d-thiogalactoside (IPTG) and grown for 5 h at 25 °C. CoREST(308–485) was expressed from a pGEX vector (gift from A. Mattevi). The plasmid was transformed into BL21-CodonPlus (DE3)-RIPL *E. coli* cells and after plating a single colony was cultivated in LB medium with 100 mg l^−1^ ampicillin at 37 °C and expression was induced at OD_600_ of 0.8 by adding 0.25 mM IPTG and grown overnight at 17 °C. The cells were pelleted by centrifugation at 4,000*g* for 30 min and stored at −80 °C before purification. All of the purification steps were performed at 4 °C. Pellets of CoREST and LSD1 were resuspended in lysis buffer (50 mM NaH_2_PO_4_ pH 8.0, 300 mM NaCl, 5% glycerol, 7.5 mM imidazole supplemented with PMSF, DNase and EDTA-free Roche protease inhibitor cocktail) at a weight ratio of 1:1.5, respectively. Cells were disrupted by sonication, clarified by centrifugation and passed through nickel-affinity resin as before. The eluent was then loaded onto GST resin equilibrated in GST affinity buffer (50 mM NaH_2_PO_4_ pH 8.0, 300 mM NaCl, 5% glycerol, 1 mM DTT, 1 mM EDTA) and the GST-tag was cleaved on the resin after incubation with GST-PreScission protease (APEXBIO) overnight at 4 °C. The protein was eluted by washing the column with GST affinity buffer, concentrated and subsequently gel-filtered on a Superdex 200 10/300 GL column equilibrated in storage buffer as before. The purity of the complex was verified by SDS–PAGE and fractions with 90–95% purity were pooled and stored at −80 °C.

Recombinant LSD1–CoREST–HDAC complex comprised full-length LSD1 (UniProt: O60341) or LSD1(Δ77–86), full-length HDAC1 (UniProt: Q13547) and N-terminally truncated CoREST (amino acids 86–485) (UniProt: Q9UKL0) or N-terminal Cys CoREST^17^. The pcDNA3 vector was used to create plasmids encoding the different proteins. The CoREST constructs contained an N-terminal 10×His–3×FLAG tag followed by a TEV protease cleavage site. The constructs for ternary complex were co-transfected into suspension-grow HEK293F cells (Thermo Fisher Scientific) with polyethylenimine (PEI) (Sigma-Aldrich) and collected after 48 h. Cells were resuspended in lysis buffer (50 mM HEPES, pH 7.5, 100 mM KCl, 5% glycerol, 0.3% Triton X-100, 1× Roche EDTA-free cOmplete protease inhibitor cocktail) and sonicated. The lysates were clarified by centrifugation at 12,000 rpm for 30 min, and the supernatant was incubated with anti-FLAG M2 affinity gel (Sigma-Aldrich). The affinity gel was washed twice with lysis buffer and twice with SEC buffer (50 mM HEPES, pH 7.5, 50 mM KCl, 0.5 mM TCEP) followed by the incubation with TEV protease overnight at 4 °C. The complex was further purified by size-exclusion chromatography using the Superose 6 10/300 column (GE Healthcare). The purity of the complex was verified by SDS–PAGE and fractions with 90–95% purity were pooled and supplemented with 5% glycerol and stored at −80 °C.

### Fluorescein labelling of LHC

The fluorescein labelling of the LSD1–CoREST–HDAC1 complex was purified as described above. A Cys point mutagenesis was conducted next to the TEV protease cleavage site of N-terminally truncated CoREST for the ligation reaction with NHS-fluorescein^60^. A 2 mM NHS-fluorescein was incubated with 500 mM mercaptoethanesulfonate (MESNA) in the reaction buffer (100 mM HEPES, pH 7.5, 50 mM KCl, 1 mM TCEP) for 4 h at room temperature in the dark for transesterification. The LSD1–CoREST–HDAC1 complex purified by FLAG M2 affinity gel was washed with reaction buffer and incubated with TEV protease for 5 h at 4 °C. The complex was then mixed with 500 μl of the fluorescein/MESNA solution to make a final concentration of 0.5 mM fluorescein and 125 mM MESNA. The mixture was incubated for 48 h at 4 °C in the dark. The complex was desalted by a Zeba spin desalting column (7 kDa MWCO) and further purified by size-exclusion chromatography using a Superose 6 10/300 column (GE Healthcare). Fluorescein-labelling efficiency was analysed by SDS–PAGE and fluorescence gel imaging (Amersham Typhoon FLA 9500, Cytiva). The purity of the complex was verified by SDS–PAGE and fractions with 90–95% purity were pooled and supplemented with 5% glycerol and stored at −80 °C.

### FP measurements

#### Titration of KBTBD4.

Recombinant WT KBTBD4 was diluted to 15 μM in a one-to-one mixture of ligand buffer (50 mM Tris-HCl, pH 8.0 cold, 150 mM NaCl, 1 mM TCEP, 10% glycerol) and LHC buffer (20 mM HEPES pH 7.5, 1 mM TCEP, 2 mg ml^−1^ BSA, 0.1% Tween-20, ±100 μM InsP_6_) containing 10 nM **JL1** with or without 20 nM recombinant LHC, HDAC1–CoREST, HDAC2–CoREST, HDAC1, HDAC2 or LSD1–CoREST. This was aliquoted in triplicate into a black 384-well plate (Corning), followed by twofold serial dilution in assay buffer containing 10 nM **JL1** with or without 20 nM recombinant LHC, HDAC1-CoREST, HDAC2-CoREST, HDAC1, HDAC2 or LSD1–CoREST (final volume, 25 μl). The plate was incubated at room temperature for 1 h and read (1,700 ms integration) using the SpectraMax i3x system with a rhodamine FP cartridge and SoftMax Pro software (Molecular Devices). Wells containing only assay buffer were used for background subtraction. The *G*-factor was adjusted to set the polarization of assay buffer with 10 nM **JL1** and 200 nM LHC only to a reference value of 27 mP. Curves were fitted to the sigmoidal, 4PL model in GraphPad Prism 9.

#### Titration of SAHA or UM171.

Recombinant WT KBTBD4 (5 μM) and recombinant LHC (20 nM) were diluted to in a one-to-one mixture of ligand buffer (50 mM Tris-HCl, pH 8.0 cold, 150 mM NaCl, 1 mM TCEP, 10% glycerol) and LHC buffer (20 mM HEPES pH 7.5, 1 mM TCEP, 2 mg ml^−1^ BSA, 0.1% Tween-20, 100 μM InsP_6_) containing 10 nM **JL1** and 10 μM SAHA or UM171. This was aliquoted in triplicate into a black 384-well plate (Corning), followed by twofold serial dilution in assay buffer containing 10 nM **JL1**, recombinant WT KBTBD4 (5 μM) and recombinant LHC (20 nM) (final volume, 25 μl). The plate was incubated at room temperature for 1 h and read (1,700 ms integration) using the SpectraMax i3x system with a rhodamine FP cartridge and SoftMax Pro software (Molecular Devices). Wells containing only assay buffer were used for background subtraction. The *G*-factor was adjusted to set the polarization of assay buffer with 10 nM **JL1**, 5 μM KBTBD4 and 200 nM LHC only to a reference value of 27 mP. Curves were fit to the sigmoidal, 4PL model in GraphPad Prism 9.

### Microscale thermophoresis measurements

MST assays were performed with the Monolith NT.115 (NanoTemper) system using the Nano BLUE mode. The exciting laser power was set at 50% and MST power was set to medium. *K*_D_ values were calculated using MO.analysis (v.2.3) software with the quadratic equation binding *K*_D_ model shown below:

$$AB=\frac{\left(A_{\text{T}}+B_{\text{T}}+K_{\text{D}}\right)-\sqrt{{\left(A_{\text{T}}+B_{\text{T}}+K_{\text{D}}\right)}^{2}-4\left(A_{\text{T}}B_{\text{T}}\right)}}{2}$$

#### Titration of KBTBD4.

Fluorescein-labelled LHC (200 nM) was titrated with WT KBTBD4 in the absence or presence of DMSO or UM171 (50 μM) in the MST-binding assays at 23 °C. WT KBTBD4 (up to 11.7 μM) was prepared with a twofold serial dilution for titrating with fluorescein–LHC. Then, 50 μM UM171 or an equivalent amount of DMSO were added. LHC (or LSD1–CoREST) at a final concentration of 200 nM was then added, mixed well and incubated for 10 min for equilibration before transferring to MST premium capillaries. Prism 9 was used to fit the data to a four-parameter dose–response curve.

### Histone H3K9ac synthesis

The depsipeptide as Fmoc-Thr(O*t*Bu)-glycolic acid was synthesized based on a reported two-step protocol^61^. Then, H3K9ac(1–34) with a sequence as ARTKQTARKS-TGGKAPRKQL-ATKAARKSAP-A-**TOG**-G was synthesized by standard solid-phase peptide synthesis and purified by reversed-phase HPLC. The Fmoc-protected amino acids were purchased from Novabiochem except for Fmoc-Lys(Ac)-OH (EMD Millipore 852042). F40 sortase was expressed and purified as reported previously, and bacterial expression and purification of *Xenopus laevis* globular H3 (gH3; amino acids 34–135 C110A) were performed also according to a previous protocol^61^. Next, the F40-sortase-catalysed histone H3 ligation reaction was carried out between the H3K9ac (amino acids 1–34; note that the C-terminal residue is extruded) peptide and the gH3. The reaction mixture was purified by ion-exchange chromatography to obtain pure semisynthetic histone H3K9ac (C110A) characterized by MALDI-TOF MS as reported previously^62^.

### Octamer refolding and nucleosome reconstitution

146 bp Widom 601 DNA was prepared according to previously reported methods used for the nucleosome reassembly^63^. Bacterial expression and purification of *X. laevis* core histones H2A, H2B and H4 were then carried out, followed by assembly of the histone octamer and refolding as previously reported^64^. The octamer was purified by size-exclusion chromatography using the Superdex 200 10/300 GL column (GE Healthcare) and was used for nucleosome assembly with 146 bp 601 Widom DNA as reported previously^65^. The final mixture was subjected to HPLC purification (Waters, 1525 binary pump, 2489 UV-Vis detector) with a TEKgel DEAE ion-exchange column to purify the final nucleosome product. The purified nucleosome containing H3K9ac was analysed by native TBE-gel with EtBr staining, as well as SDS–PAGE gel and then western blot analysis using anti-H3K9ac antibodies^65^.

### Analysis of LHC complex deacetylation of acetylated nucleosome

The general deacetylation assay was set up as reported previously^66^. The LHC complex was diluted into the pH 7.5 reaction buffer containing 50 mM HEPES, 100 mM KCl, 0.2 mg ml^−1^ BSA and 100 μM InsP_6_ to a final concentration of 90 nM. After the addition of KBTBD4 to a final concentration of 300 nM and/or UM171 (in final 10% DMSO) to a final concentration of 10 μM, the solution was preincubated for 15 min at ambient temperature. After chilling on ice for 3 min, the deacetylation reaction was initiated with the addition of H3K9ac nucleosome to a final concentration of 100 nM, and all of the reaction solutions were incubated for 120 min at 37 °C. Different aliquots were taken at timepoints of0, 30 min, 60 min, 90 min and 120 min. Each aliquot was quenched with an SDS-loading buffer containing 20 mM EDTA, and was heated at 95 °C for 3 min. After running SDS–PAGE and iBlot transfer to nitrocellulose membranes, western blot analysis was performed with anti-H3K9ac primary antibody (Abcam, AB32129, 1:2,000), followed by the goat anti-rabbit secondary antibody (Cell Signaling Technology, 7074S, 1:2,000). Western blot analysis with anti-H3 (Abcam, AB1791, 1:2,000) was used as the loading control. Imaging analysis with chemiluminescence on GeneSys was quantified using ImageJ software^62^. All intensity values were fit to a single-phase exponential decay curve with constrain Y0 = 1, plateau=0 (GraphPad Prism Ten). Each plotted point represents two replicates for the kinetic parameter *V*/[*E*] calculation.

### HDAC1/2 activity assays

Recombinant HDAC1 (BPS Bioscience 50051) or HDAC2 (BPS Bioscience 50002) were diluted to 6 nM (1.2×) into buffer containing 50 mM HEPES, pH 7.5, 100 mM KCl, 0.5 mg ml^−1^ BSA, 0.001% Tween-20 and 25 μl added to wells of a white, 384-well microtitre plate (Corning 3572). Test compounds were added in serial dilution (1:2 titration, 15-point, *c*_max_ = 10 μM) using a D300 digital dispenser (Hewlett-Packard), and allowed to equilibrate for 1 h at room temperature. Then, 5 μl of 6× MAZ1600 HDAC substrate^28^ was added (final HDAC1/2 concentration 5 nM; final MAZ1600 concentration 18 μM) and deacetylase activity was allowed to proceed for 45 min at room temperature. Next, 5 μl of 7× developer solution was added (150 nM trypsin + 40 μM LBH589 final concentrations) and the plate was incubated for 30 min at room temperature. 7-Amino-4-methyl coumarin fluorescence was measured on the Tecan Spark plate reader: 350/20 nm excitation, 460/10 nm emission. The assay floor (background) was defined with the 10 μM LBH589 dose, and the assay ceiling (top) was defined through a no-inhibitor control. Data were background-corrected, normalized and Prism 9 was used to fit the data to a four-parameter dose–response curve.

### TR-FRET measurements

Unless otherwise noted, experiments were performed in white, 384-well microtitre plates (Corning, 3572) at a 30 μl assay volume, or white, 384-well low-volume microtitre plates (PerkinElmer, 6008280). TR-FRET measurements were acquired on a Tecan SPARK plate reader with SPARKCONTROL software v.2.1 (Tecan) with the following settings: 340/50 nm excitation, 490/10 nm (Tb) and 520/10 nm (FITC, AF488) emission, 100 μs delay, 400 μs integration. The 490/10 nm and 520/10 nm emission channels were acquired with a 50% mirror and a dichroic 510 mirror, respectively, using independently optimized detector gain settings unless specified otherwise. The TR-FRET ratio was taken as the 520/490 nm intensity ratio on a per-well basis.

### Ternary complex measurements by TR-FRET

#### Titration of UM171.

Recombinant WT 6×His–KBTBD4 (40 nM), fluorescein-labelled LSD1–CoREST–HDAC complex (40 nM) and CoraFluor-1-labelled anti-6×His IgG (20 nM)^33^ were diluted into a one-to-one mixture of ligand buffer (50 mM Tris-HCl, pH 8.0, 150 mM NaCl, 1 mM TCEP, 10% glycerol) and LHC buffer (20 mM HEPES, pH 7.5, 1 mM TCEP, 2 mg ml^−1^ BSA, 0.1% Tween-20, 100 μM InsP_6_), with or without 100 μM SAHA, and 10 μl was added to wells of a white, 384-well low-volume microtitre plate (PerkinElmer, 6008280). UM171 was added in serial dilution (1:3 titration, 10-point, *c*_max_ = 10 μM) using a D300 digital dispenser (Hewlett-Packard) and allowed to equilibrate for 1 h at room temperature before TR-FRET measurements were taken. Data were background-corrected from wells containing no UM171. Prism 9 was used to fit the data to a four-parameter dose–response curve.

#### Titration of UM171 and InsP_6_.

Recombinant WT 6×His–KBTBD4 (40 nM), fluorescein-labelled LSD1-CoREST-HDAC complex (40 nM) and CoraFluor-1-labelled anti-6×His IgG (20 nM)^33^ were diluted into were diluted into a one-to-one mixture of ligand buffer (50 mM Tris-HCl, pH 8.0, 150 mM NaCl, 1 mM TCEP, 10% glycerol) and LHC buffer (20 mM HEPES, pH 7.5, 1 mM TCEP, 2 mg ml^−1^ BSA, 0.1% Tween-20) and 10 μl was added to wells of a white, 384-well low-volume microtitre plate (PerkinElmer, 6008280). UM171 was added in serial dilution (1:10 titration, 5-point, *c*_max_ = 10 μM) and InsP_6_ was added in serial dilution (1:10 titration, 6-point, *c*_max_ = 100 μM) using a D300 digital dispenser (Hewlett-Packard) and allowed to equilibrate for 1 h at room temperature before TR-FRET measurements were taken. Data were background-corrected from wells containing no UM171 and no InsP_6_. Prism 9 was used to fit the data to a four-parameter dose–response curve.

#### Titration of fluorescein-labelled LSD1–CoREST–HDAC complex.

Recombinant WT 6×His–KBTBD4 (10 nM, 2×) and CoraFluor-1-labelled anti-6×His IgG (5 nM, 2×)^33^ were diluted into LHC buffer, with or without 10 μM UM171, and 5 μl added to wells of a white, 384-well low-volume microtitre plate (PerkinElmer, 6008280). Serial dilutions of fluorescein-labelled LSD1–CoREST–HDAC complex (1:2 titration, 10-point, *c*_max_ = 1,000 nM, 2×) were prepared in ligand buffer and 5 μl was added to wells of the same plate (final volume, 10 μl; final 6×His–KBTBD4 concentration, 5 nM; final CoraFluor-1-labelled anti-6×His IgG concentration, 2.5 nM; fluorescein-labelled LSD1–CoREST–HDAC complex *c*_max_, 500 nM). The plate was allowed to equilibrate for 1 h at room temperature before TR-FRET measurements were taken. Data were background-corrected from wells containing no 6×His–KBTBD4. Prism 9 was used to fit the data to a four-parameter dose–response curve.

### In vitro ubiquitination assay

The ubiquitination assays were set up similarly as previously reported^67^. Reactions were performed at 37 °C in a total volume of 20 μl. The reaction mixtures contained 5 mM ATP, 100 μM WT ubiquitin, 100 nM E1 protein, 2 μM E2 protein, 0.5 μM neddylated RBX1–CUL3, 0.5 μM WT KBTBD4 (unless otherwise indicated), 10 μM UM171/DMSO with 25 mM Tris-HCl (pH 7.5), 20 mM NaCl, 10 μM InsP_6_ and 2.5 mM MgCl_2_ as reaction buffer. Substrate fluorescein–LHC at 0.5 μM was preincubated with everything except E1 in the reaction mixture at 37 °C for 5 min before adding E1 to initiate the reaction. The reactions were quenched at the indicated timepoints by adding SDS loading buffer containing reducing agent β-mercaptoethanol. The reaction samples were resolved on SDS–PAGE gels and analysed by Colloidal Blue staining and western blotting.

### Base editor scan

The sgRNA libraries were designed as described previously^68^ to include all sgRNAs (NG protospacer-adjacent motif) targeting exonic and flanking ±30 bp into the intronic regions of canonical isoforms of KBTBD4 (ENST00000430070.7) and HDAC1 (ENST00000373548.8), excluding those with TTTT sequences as well as negative (nontargeting, intergenic) and positive (essential splice site) controls. The library was synthesized as an oligonucleotide pool (Twist Biosciences) and cloned into pRDA_478 and pRDA_479 following published workflows. Lentivirus was produced and titred by measuring cell counts after transduction and puromycin selection. Cells were transduced with library lentivirus at an MOI < 0.3 and selected with puromycin for 5 days. Cells were then expanded and split into three replicate subcultures and treated with DMSO or 1 μM UM171. After 24 h, cells were sorted on a MoFlo Astrios EQ Cell Sorter (Beckman Coulter), collecting the top 10% GFP^+^ and unsorted (GPF^±^) cells. Genomic DNA was isolated using the QIAamp DNA Blood Mini kit, and sgRNA sequences were amplified using barcoded primers, purified by gel extraction and sequenced on the Illumina MiSeq platform as previously described^52,69^. At all steps, sufficient coverage of the library was maintained in accordance with published recommendations.

Data analysis was performed using Python (v.3.9.12) with Biopython (v.1.78), Pandas (v.1.5.1), SciPy package (v.1.10.0) and NumPy (v.1.23.4). sgRNA enrichment was calculated as previously described^52,69^. In brief, sequencing reads matching each sgRNA were quantified as reads per million, increased by a pseudocount of 1, log_2_-transformed, normalized to the plasmid library and replicate-averaged. Sorted GFP^+^ abundances were normalized to unsorted abundances. The mean value for non-targeting controls was subtracted to calculate the final enrichment value for each sgRNA; this value is referred to as the normalized log_2_[fold change in sgRNA enrichment]. sgRNAs with zero counts in the plasmid libraries were excluded from further analysis.

sgRNAs with scores of >4 s.d. above or below the mean of intergenic negative controls were considered to be enriched or depleted, respectively. sgRNAs targeting KBTBD4 and HDAC1 were classified based on expected editing outcome, assuming any C or A within the editing window (protospacer +4 to +8) of cytidine and adenosine base editors, respectively, is converted to T or G. sgRNAs were placed in one of six mutually exclusive classes: in order of assignment priority: (1) nonsense; (2) missense; (3) silent; (4) UTR-intronic; (5) non-editing (no Cs and/or As); (6) negative controls (does not target gene). Library sgRNA annotations and base editor scanning data are provided in Supplementary Data 7–14. Scatter and line plots were generated using matplotlib (v.3.7.1).

### Linear clustering analysis

Per-residue sgRNA enrichment scores were estimated as previously described^45^. In brief, LOESS regression was performed on using the lowess function of the statsmodels package (v.0.13.5) in Python (v.3.9.12) with a 20 amino acid sliding window (‘frac = (20 AA/*L*)’, where *L* is the total length of the protein), and ‘it = 0’ to fit observed log_2_[fold change in sgRNA enrichment], hereafter the sgRNA enrichment score, as a function of amino acid position. Only sgRNAs that are predicted to result in missense mutations were used. For amino acid positions that were not targeted by sgRNAs, enrichment scores were interpolated by performing quadratic spline interpolation on the LOESS output scores using the interp1d function of the SciPy package (v.1.10.0).

To assess statistical significance of the resulting clusters, we simulated a null model of random sgRNA enrichment scores. Amino acid positions of sgRNAs were kept fixed while sgRNA enrichment scores were randomly shuffled, and per-residue enrichment scores were recalculated by performing LOESS regression and interpolation on the randomized sgRNA enrichment scores for each of 10,000 permutations. Empirical *P* values were calculated for each amino acid by comparing its observed resistance score to the null distribution of random resistance scores. Empirical *P* values were adjusted using the Benjamini–Hochberg procedure to control the FDR to ≤0.05. Finally, linear clusters were called by identifying all contiguous intervals of amino acids with adjusted *P* ≤ 0.05. For plotting, adjusted *P* values were increased by a pseudocount of 10^−4^, log_10_-transformed and multiplied by −1.

### Genotyping

Genomic DNA was purified using the QIAamp DNA Blood Mini (Qiagen) or QuickExtract DNA Extraction Solution (Biosearch Technologies) according to the manufacturer’s protocol. We subjected 100 ng of DNA to a first round of PCR (25–28 cycles, Q5 hot-start high-fidelity DNA polymerase (New England Biolabs)) to amplify the locus of interest and attach common overhangs. Then, 1 μl of each PCR product was amplified in a second round of PCR (8 cycles) to attach barcoded adapters. Final amplicons were purified by gel extraction (Zymo) and sequenced on an Illumina MiSeq. Data were processed using CRIS-PResso2^70^ using the following parameters: --quantification_window_size 20 --quantification_window_center -10 --plot_window_size 20 --exclude_bp_from_left 0 --exclude_bp_from_right 0 --min_average_read_quality 30 --n_processes 12 --base_editor_output.

### Generation of HDAC1 mutant clones

sgRNAs enriched in the base editing screens were ordered as synthetic oligonucleotides (Azenta/Genewiz), annealed, and ligated into either pRDA_478 or pRDA_479. The plasmids were transfected into HEK293T cells using Lipofectamine 3000 (Thermo Fisher Scientific) according to the manufacturer’s protocol. Then, 48 h after transduction, cells were selected with 2 μg ml^−1^ puromycin (Thermo Fisher Scientific) for 3 days, then sorted for single-cell clones on the BD FACSAria Cell Sorter (BD Biosciences). Single-cell clones were validated by genotyping and the stability of mutants was assessed by immunoblotting. sgRNA sequences and annotations, as well as primer sequences used for genotyping are provided in Supplementary Tables 3 and 4, respectively.

### Single guide validation in K562

sgRNAs enriched in the KBTBD4 CBE screen were ordered as synthetic oligonucleotides (Azenta/Genewiz), annealed and ligated into SpG Cas9 NG PAM of the pRDA_256 plasmid. Lentivirus was produced as described above and transduced into CoREST–GFP K562 cells. After puromycin selection, cells were collected and validated by genotyping. sgRNA sequences and annotations, as well as primer sequences used for genotyping are provided in Supplementary Tables 3 and 4, respectively.

### Degradation assay of KBTBD4 mutants

K562 *KBTBD4*-null CoREST–GFP cells were generated as described above. KBTBD4 overexpression constructs were cloned into pSMAL mCherry and point mutations were introduced into coding regions using standard PCR-based site-directed mutagenesis techniques. Lentiviral particles carrying the overexpression constructs were produced and used to transduce K562 *KBTBD4*-null CoREST–GFP cells as described above. Then, 48 h after transduction, cells were treated with 1 μM UM171, or 0.1% DMSO for 24 h. The GFP^+^ percentage was measured for mCherry^+^ cells in each condition and FACS gating schemes are shown in Supplementary Fig. 1c.

### Production of eVLPs

Engineered virus-like particles (eVLPs) were produced as previously described^71^. In brief, Gesicle Producer 293T cells were seeded into T-75 flasks (Corning) at a density of 5 × 10^6^ cells per flask. After 20–24 h, a mixture of plasmids expressing VSV-G (400 ng), MMLVgag–pro–pol (3,375 ng), MMLVgag–3×NES–ABE8e (1,125 ng) and an sgRNA (4,400 ng) were co-transfected into each T-75 flask using jetPRIME transfection reagent (Polyplus) according to the manufacturer’s protocols. Then, 40–48 h after transfection, the producer cell supernatant was collected and centrifuged for 10 min at 4 °C and 2,000*g* to remove the cell debris. The clarified eVLP-containing supernatant was filtered through a 0.45 μm PVDF filter (Sigma-Aldrich). The filtered supernatant was concentrated by ultracentrifugation using a cushion of 20% (w/v) sucrose (Sigma-Aldrich) in PBS. Ultracentrifugation was performed at 26,000 rpm for 2 h at 4 °C using an SW28 rotor in an Optima XE-90 Ultracentrifuge (Beckman Coulter). After ultracentrifugation, eVLP pellets were resuspended in cold PBS (pH 7.4). eVLPs were frozen and stored at −80 °C. eVLPs were thawed on ice immediately before use and repeated freeze–thaw was avoided.

### eVLP transduction in cell culture

K562 cells were plated for transduction in 96-well plates (Cellstar Greiner Bio-one) at a density of 50,000 cells per well with 5 μg ml^−1^ polybrene (Santa Cruz Biotechnology) medium. BE-eVLPs were added directly to the culture medium in each well. Then, 50 μl of fresh medium was added after 6 h, and another 100 μl of medium was added at 48 h after transduction. Then, 72 h after transduction, cellular genomic DNA was isolated and genotyped as described below. Transduced cells were allowed to recover for 7–10 days before degradation assays were performed.

### Cryo-EM sample preparation and data collection

To assemble the complex of KBTBD4–UM171–LHC for the cryo-EM study, the individually isolated KBTBD4 protein and co-expressed LHC complex were mixed in stoichiometric amounts with 1 μM UM171 added and subsequently applied to the Superose6 increase gel-filtration column (Cytiva) in a buffer containing 40 mM HEPES, pH 7.5, 50 mM KCl, 100 μM InsP_6_ and 0.5 mM TCEP (Tris(2-carboxyethyl) phosphine). The isolated complex was then cross-linked with 37.5 mM glutaraldehyde at room temperature for 6 min and quenched with 1 M Tris-HCl pH 8.0. The cross-linked sample was snap-frozen for future use.

To prepare grids for cryo-EM data collection, a QuantiFoil Au R0.6/1 grid (Electron Microscopy Sciences) was glow discharged for 30 s at 20 mA with a glow discharge cleaning system (PELCO easiGlow). 3.0 μl of the purified KBTBD4-UM171-LHC complex at 0.7 mg ml^−1^ was applied to a freshly glow-discharged grid. After incubating in the chamber at 10 °C and 100% relative humidity, the grids were blotted for 3 s with a blotting force of zero, then immediately plunge-frozen in liquid ethane using the Vitrobot Mark IV system (Thermo Fisher Scientific). Data collection was performed on the FEI Titan Glacios transmission electron microscope (Thermo Fisher Scientific) operated at 200 kV at the Arnold and Mabel Beckman Cryo-EM Center of the University of Washington. The automation scheme was implemented using the SerialEM^72^ software (v.4.1.8) using beam-image shift^73^ at a nominal magnification of ×105,000, resulting a physical pixel size of 0.885 Å. The images were acquired on a K3 camera direct detector. The dose rate was set to 10 e^−^ Å^−2^ s^−1^, and the total dose of 50 electrons per Å^2^ for each image was fractionated into 99 electron-event representation frames. Data were collected in four sessions with a defocus range of 0.8–1.8 μm. In total, 6,839 videos were collected.

To prepare grids for the cryo-EM study of apo KBTBD4, a QuantiFoil Au R1.2/1.3 grid (Electron Microscopy Sciences) was glow discharged for 30 s at 20 mA with a glow discharge cleaning system (PELCO easiGlow). Then, 3.0 μl of purified KBTBD4, with a final concentration of 0.1% *n*-decyl-β-d-maltoside and a protein concentration of 4 mg ml^−1^ was applied to a freshly glow-discharged grid. After incubating in the chamber at 10 °C and 100% relative humidity for 60 s, grids were blotted for 3 s with a blotting force of zero, then immediately plunge-frozen in liquid ethane using a Vitrobot Mark IV system (Thermo Fisher Scientific). Data collection was performed on the FEI Titan Glacios transmission electron microscope (Thermo Fisher Scientific) operated at 200 kV at the Arnold and Mabel Beckman Cryo-EM Center of the University of Washington. Automation scheme was implemented using the SerialEM^72^ software using beam-image shift^73^ at a nominal magnification of ×105,000, resulting a physical pixel size of 0.885 Å. The images were acquired on a K3 camera direct detector. The dose rate was set to 10 e^−^ Å^−2^ s^−1^, and the total dose of 50 e^−^ Å^−2^ for each image were fractionated into 99 electron-event representation frames. Data were collected in four sessions with a defocus range of 0.8–1.8 μm. In total, 11,263 videos were collected.

### Image processing and 3D reconstruction

For the KBTBD4–UM171–LHC structure, a total of 10,816 videos were collected and imported into CryoSPARC^74^ followed by patch motion correction and patch contrast transfer function (CTF) estimation. In total, 10,637 micrographs were retained after filtering the micrographs with CTF parameters and manual inspection. The blob picker job in CryoSPARC was able to pick 7,133,729 particles, which were further extracted and subjected to 2D classification. After five rounds of cleaning by 2D classification, 928,437 particles were selected and subjected to ab initio reconstruction. Subsequently, all of the particles were used for heterogenous refinement. After one extra round of cleaning up by heterogenous refinement, 186,315 particles from good reconstruction were selected to get re-extracted without Fourier cropping. The homogenous refinement and non-uniform refinement^75^ helped to reach an overall resolution of 3.93 Å. To optimize the map for the KELCH-repeat domain, two different soft masks focused on the BTB-BACK-KELCH domain in chain A and HDAC1–CoREST-KELCH in chain B was applied to local refinement, respectively, which led to a further improved resolution of 3.77 Å and 3.86 Å. The two maps provided clearer density for the KBTBD4 protomer A and CoREST. Further details about the data processing are provided in Extended Data Fig. 4.

For the apo KBTBD4 structure, in total, 11,263 videos were collected and imported into CryoSPARC^74^ followed by patch motion correction and patch CTF estimation. In total, 10,057 micrographs were retained after filtering the micrographs with CTF parameters and manual inspection. The blob picker job in CryoSPARC was able to pick 1,039,200 particles, which were further extracted and subjected to 2D classification. A total of 147,826 particles was used for primary ab initio reconstruction, from which the templates were generated, and template picker was conducted to pick 8,280,266 particles. After two rounds of cleaning by 2D classification, 340,735 particles were selected and subjected to Topaz picking. Subsequently, after two rounds of cleaning by 2D classification, 766,539 particles were used for ab initio reconstruction and heterogenous refinement. After one extra round of cleaning up by heterogenous refinement, 572,349 particles from good reconstruction were selected to get re-extracted without Fourier cropping. The homogenous refinement and non-uniform refinement^75^ helped to reach an overall resolution of 3.83 Å. Further details about the data processing are provided in Extended Data Fig. 5.

### Model building and refinement

The initial structural models of the KBTBD4 dimer and the HDAC1–CoREST–ELM–SANT1 complex was predicted with AlphaFold-Multimer in Google ColabFold2^76^. The structural models of KBTBD4 BTB-BACK domain, KELCH-repeat domain, and HDAC1–CoREST were separately fit into the cryo-EM map using UCSF ChimeraX-1.7 (rc2023.12.12)^77^. The resulting model was subsequently rebuilt in Coot (v.0.9.8.91)^78^ based on the protein sequences and the EM density and was further improved by real-space refinement in PHENIX (v.1.20.1-4487-000)^79,80^. The structure figures were made using PyMOL (v.2.5.4)^81^.

### Reporting summary

Further information on research design is available in the Nature Portfolio Reporting Summary linked to this article.

## Data availability

The coordinates and the cryo-EM maps of KBTBD4–UM171–LHC–InsP_6_ and the apo form of KBTBD4 were deposited at the PDB under accession numbers 8VOJ and 9DTG, and in the Electron Microscopy Data Bank (EMDB) under accession numbers EMD-43386 and EMD-47155, respectively. MS-based proteomics are provided in Supplementary Data 1 and 2 and 4–6, and original mass spectra have been deposited in the public proteomics repository MassIVE under dataset identifiers MSV000096487 and MSV000096456. Base editor scanning data, genotyping analysis results, oligonucleotide sequences as well as additional data generated by this study are provided in the Supplementary Information and Source data. The following publicly available datasets were used: PDB 4BKX, 4LXZ and 5ICN. Source data are provided with this paper.

## Code availability

Code generated for data analysis is available at GitHub (https://github.com/liaulab/HDAC1_KBTBD4_base_editing_scanning_2024).

## Extended Data

**Extended Data Table 1 |:** Cryo-EM data collection, refinement and validation statistics

	KBTBD4-UM171-LHC InsP_6_(mask1)	KBTBD4-UM171-LHC InsP_6_(mask2)	Apo KBTBD4
**Data collection and processing**			
Microscope and detector	Glacios/Gatan K3	Glacios/Gatan K3	Glacios/Gatan K3
Magnification	105,000	105,000	105,000
Voltage (kV)	200	200	200
# of movies	10,816	10,816	11,263
Electron exposure (e^−^/Å^2^)	50	50	50
Defocus range (μm)	−0.8 to −1.8	−0.8 to −1.8	−0.8 to −1.8
Pixel size (Å)	0.885	0.885	0.885
Symmetry imposed	C1	C1	C1
# of particles from blob/template picking (after 1st clean-up)	8,624,634	8,624,634	11,214,475
# of initial extracted particle images	7,133,729	7,133,729	7,088,218
# of final particle images	218,279	218,279	550,442
Map resolution (Å)	3.77	3.86	3.83
FSC threshold	0.143	0.143	0.143
CC_mask_/CC_volume_	0.76/0.72	0.69/0.66	0.75/0.70
B factor (Å^2^)	117.2	94.8	150.3
**Refinement and validation**			
Initial model used	LHC-K4wt-UM171-InsP_6_ (AlphaFold)	LHC-K4wt-UM171-InsP_6_ (AlphaFold)	LHC-K4wt-UM171-InsP_6_ (AlphaFold)
Model composition			
Nonhydrogen atoms	12109	12109	8069
Protein residues	1508	1508	1023
Ligand	3	3	3
R.m.s. deviations			
Bond lengths (Å)	0.003	0.003	0.003
Bond angles (°)	0.604	0.680	0.651
B-factors (min/max/mean) [ligand]	52.34/111.5/82.37	27.11/149.08/125.31	
MolProbity score	2.47	2.1	1.93
Clashscore	10.98	8.25	7.26
Poor rotamers (%)	3.65	1.9	0.79
Ramachandran plot			
Favored (%)	92.1	93.11	90.56
Allowed (%)	7.83	6.89	9.24
Outliers (%)	0.00	0	0.2

KBTBD4-UM171-LHC-IP6 (mask1) and KBTBD4-UM171-LHC-IP6 (mask2) refer to the complex structures of KBTBD4 bound to UM171, LSD1-HDAC1-CoREST, and Inositol hexakisphosphate locally refined with a mask excluding the KBTBD4-A protomer and the BTB domain of KBTBD4-B protomer or excluding the KBTBD4-B protomer and HDAC1-CoREST, respectively. Apo KBTBD4 refers to the structure of the free KBTBD4 dimer.

## Supplementary Material

Supplementary Information

Supplementary Data Guide

Supplementary Data 5

Supplementary Data 7

Supplementary Data 8

Supplementary Data 6

Supplementary Data 9

Supplementary Data 11

Supplementary Data 10

Supplementary Data 12

Supplementary Data 13

Supplementary Data 14

Supplementary Data 1

Supplementary Data 2

Supplementary Data 3

Supplementary Data 4
