# Supplementary material for: UM171 glues asymmetric CRL3–HDAC1/2 assembly to degrade CoREST corepressors
Source: Nature. Author manuscript; Available in PMC 2025 Mar 24. (PMC11882444; doi:10.1038/s41586-024-08532-4)

nature portfolio

https://doi.org/10.1038/s41586-024-08532-4

Supplementary information

UM171 glues asymmetric CRL3–HDAC1/2 assembly to degrade CoREST corepressors

In the format provided by the authors and unedited

Supplementary Information for

UM171 Glues Asymmetric CRL3-HDAC1/2 Assembly to Degrade CoREST Corepressors

Megan J.R. Yeo, Olivia Zhang, Xiaowen Xie, Eunju Nam, N. Connor Payne, Pallavi M. Gosavi, Hui Si Kwok, Irtiza Iram, Ceejay Lee, Jiaming Li, Nicholas J. Chen, Khanh Nguyen, Hanjie Jiang, Zhipeng A. Wang, Kwangwoon Lee, Haibin Mao, Stefan A. Harry, Idris A. Barakat, Mariko Takahashi, Amanda L. Waterbury, Marco Barone, Andrea Mattevi, Steven A. Carr, Namrata D. Udeshi, Liron Bar-Peled, Philip A. Cole, Ralph Mazitschek, Brian B. Liau*, Ning Zheng*

***Correspondence:** liau@chemistry.harvard.edu, nzheng@uw.edu

Supplementary Figures

**Supplementary Figure 1**	Representative gating schemes for flow cytometry	**2**
**Supplementary Figure 2**	Uncropped western blot images in Fig. 1	**4**
**Supplementary Figure 3**	Uncropped western blot images in Fig. 2	**5**
**Supplementary Figure 4**	Uncropped western blot images in Fig. 5	**6**
**Supplementary Figure 5**	Uncropped western blot images in ED Fig. 2	**7**
**Supplementary Figure 6**	Uncropped western blot and gel images in ED Fig. 3	**8**
**Supplementary Figure 7**	Uncropped western blot images in ED Fig. 8	**9**
**Supplementary Figure 8**	Uncropped western blot images in ED Fig. 9	**10**

Supplementary Tables

**Supplementary Table 1**	sgRNA for KO experiments	**11**
**Supplementary Table 2**	Primer sequences for KO experiments	**12**
**Supplementary Table 3**	sgRNA for base editing validation	**13**
**Supplementary Table 4**	Primer sequences for base editing validation	**14**

Supplementary Methods

Synthetic Procedures and NMR spectra **16**

Supplementary Figure 1 | Representative gating schemes for flow cytometry

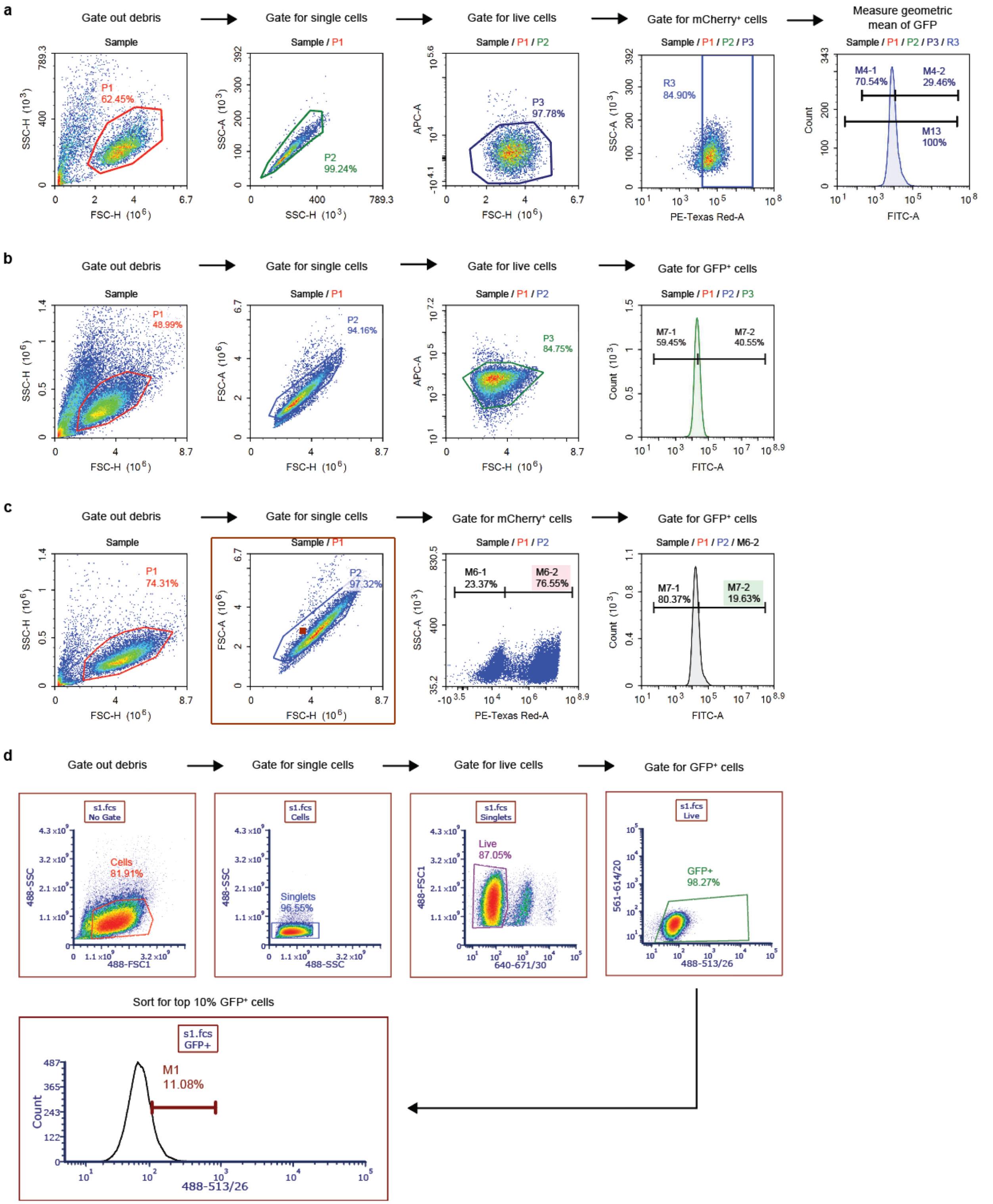

a, Representative gating scheme for flow cytometric analysis of fluorescence-based corepressor degradation assays in MOLM-13 cells. Helix NP NIR was used as a viability dye. GFP fluorescence and mCherry fluorescence were monitored on the FITC and PE-Texas Red channels,

respectively. **b**, Representative gating scheme for flow cytometric analysis of CoREST-GFP degradation by KBTBD4. Helix NP NIR was used as a viability dye. GFP fluorescence was monitored on the FITC channels. **c**, Representative gating scheme for flow cytometric analysis of CoREST-GFP degradation by KBTBD4 overexpression in KBTBD4-null K562 cells. GFP fluorescence and mCherry fluorescence were monitored on the FITC and PE-Texas Red channels, respectively. **d**, Representative gating scheme for FACS-based sorting of base editor screens in CoREST-GFP K562 cells. Helix NP NIR was used as a viability dye. GFP fluorescence was monitored on the FITC channels.

Supplementary Figure 2 | Uncropped western blot images in Figure 1

Fig 1c

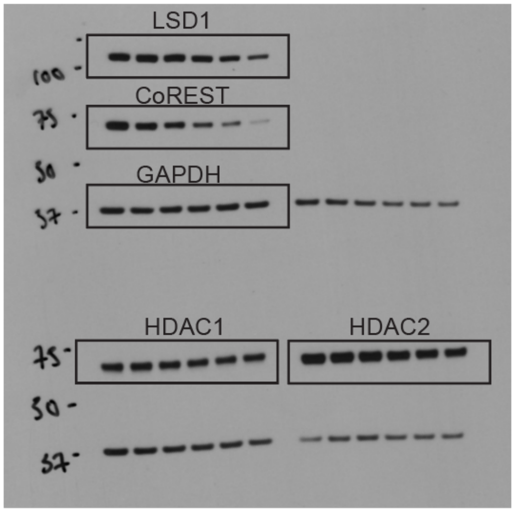

Fig 1g

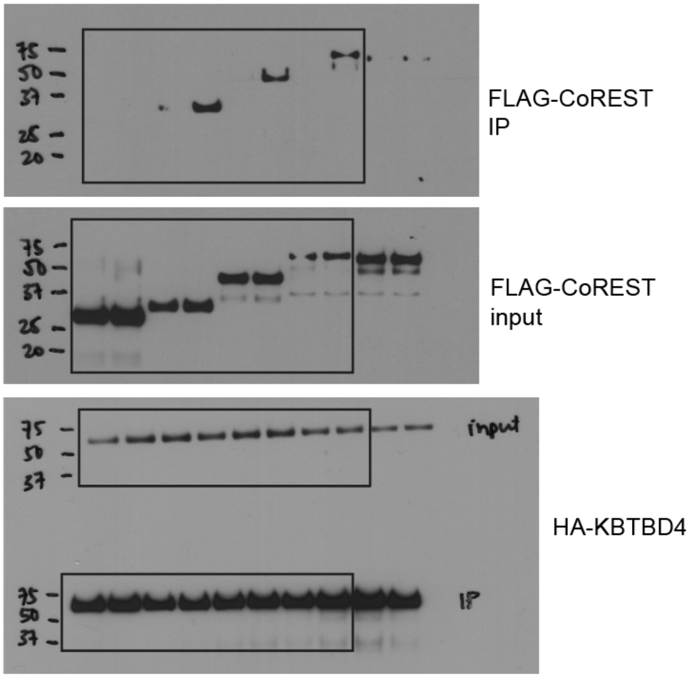

Supplementary Figure 3 | Uncropped western blot images in Figure 2

Fig 2d - same blot, different exposures

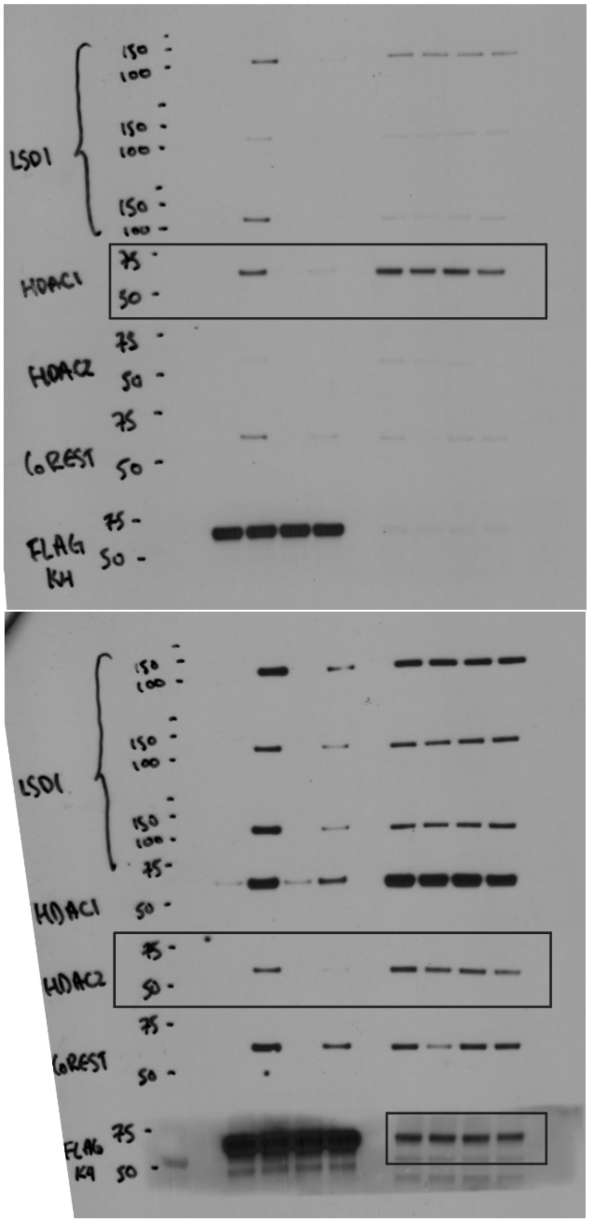

Fig 2d cont'd - same blot, different exposures

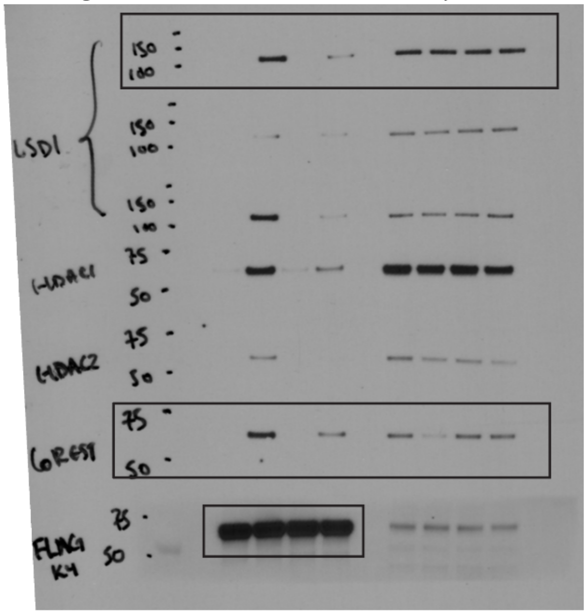

Fig 2i

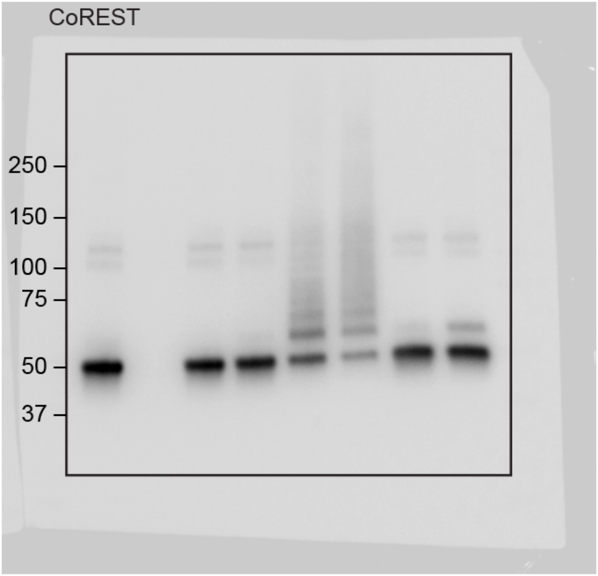

Supplementary Figure 4 | Uncropped western blot images in Figure 5

Fig 5d

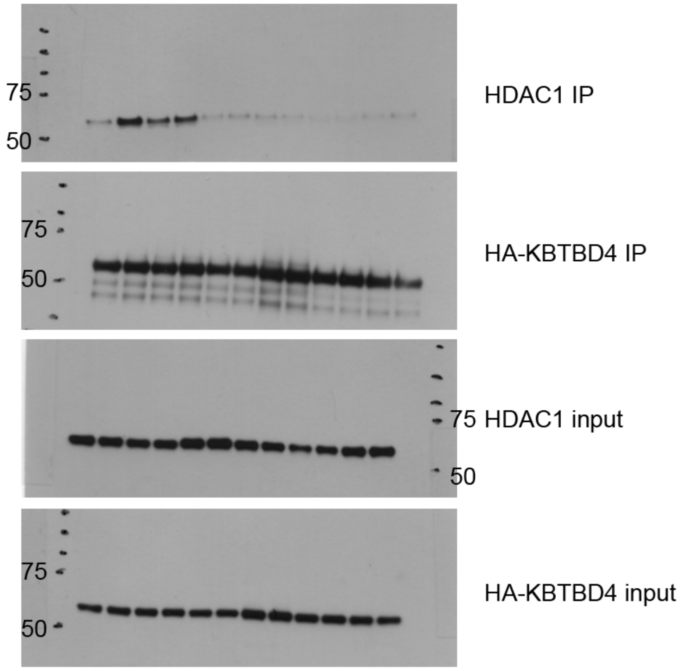

Supplementary Figure 5 | Uncropped western blot images in Extended Data Figure 2

ED Fig 2a

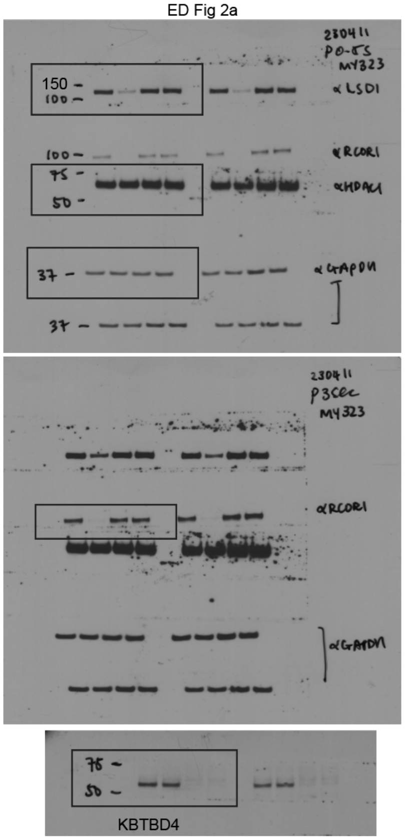

ED Fig 2c - same blot, different exposures

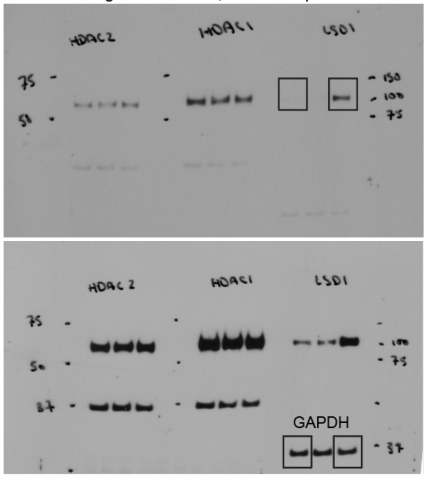

ED Fig 2d

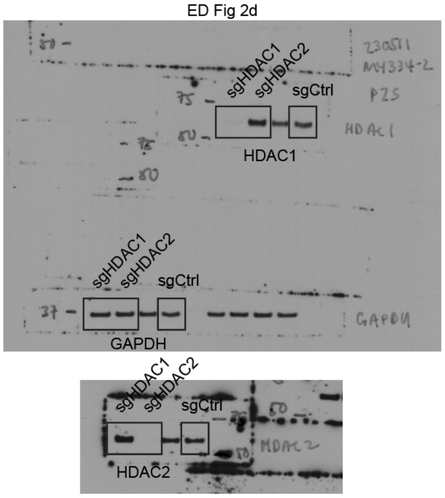

ED Fig 2e

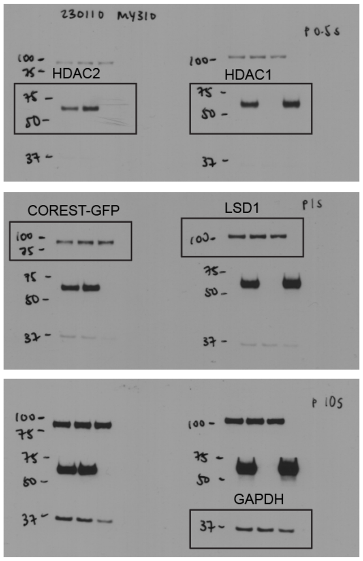

ED Fig 2g

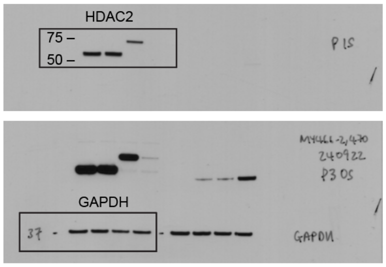

Supplementary Figure 6 | Uncropped western blot and gel images in Extended Data Figure 3

ED Fig 3c

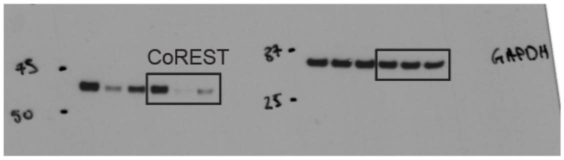

ED Fig 3f

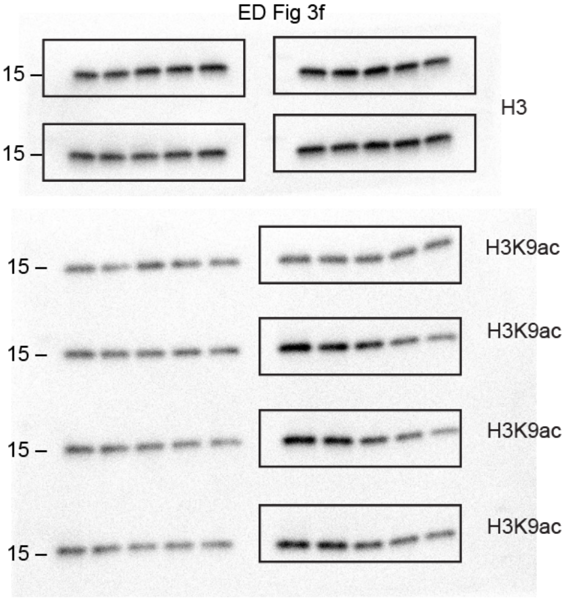

ED Fig 3i

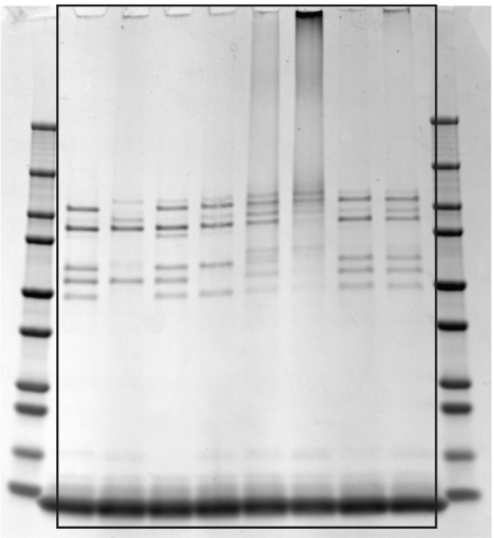

ED Fig 3j

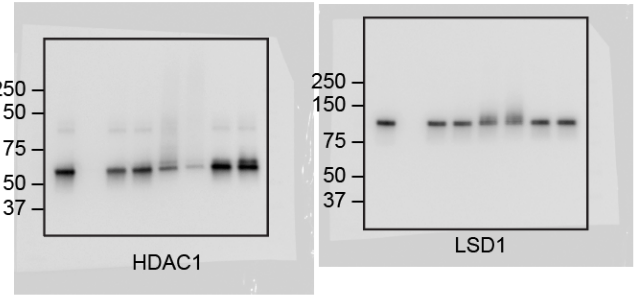

Supplementary Figure 7 | Uncropped western blot images in Extended Data Figure 8

ED Fig 8a

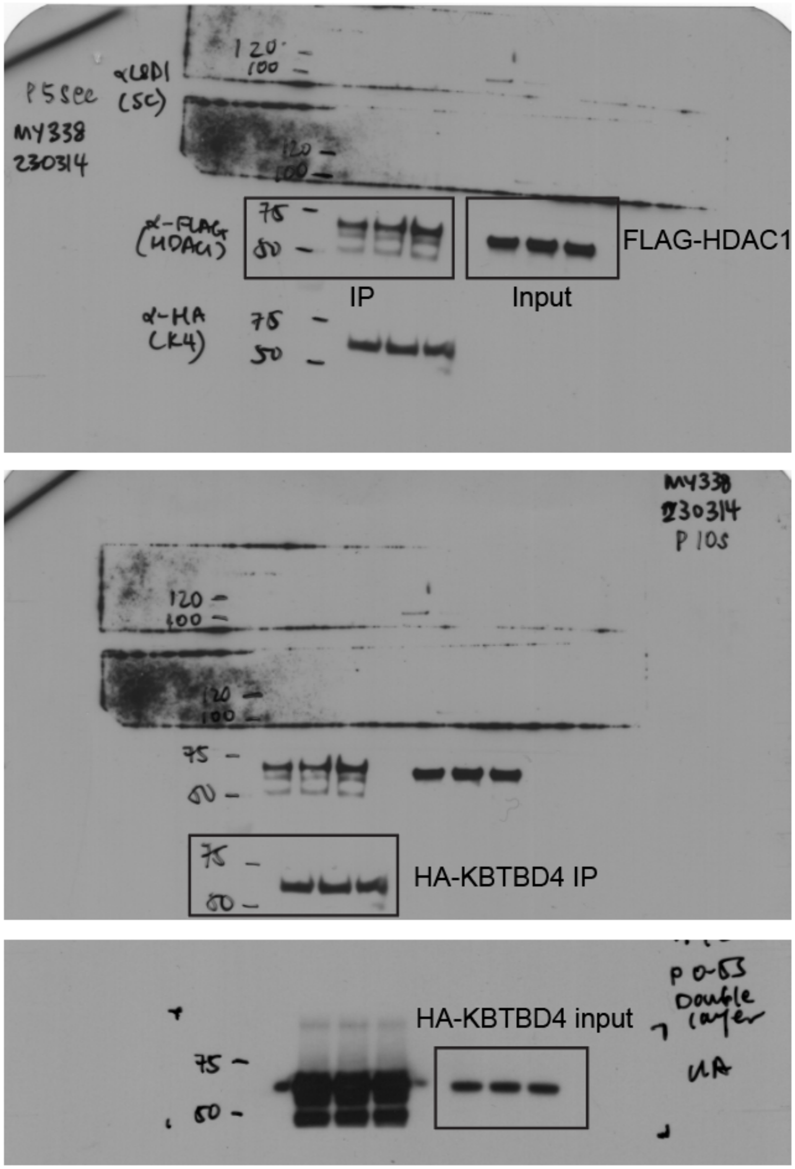

Supplementary Figure 8 | Uncropped western blot images in Extended Data Figure 9

ED Fig 9d

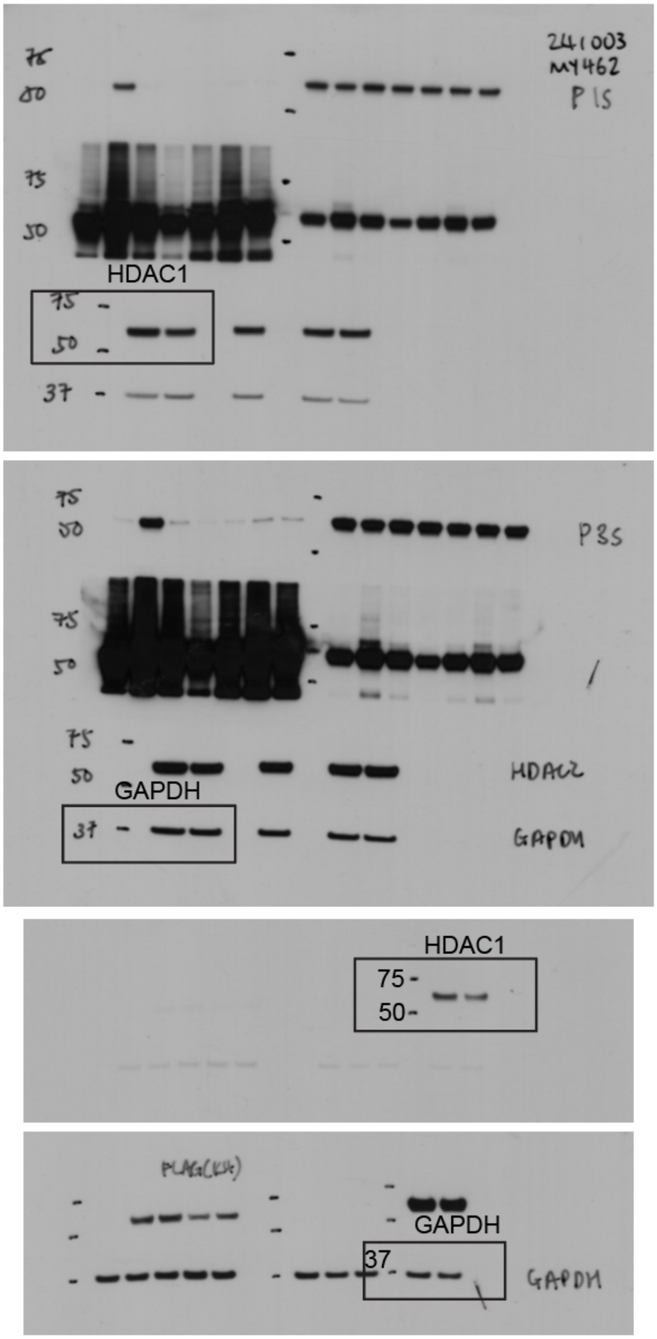

Supplementary Table 1 | sgRNA for KO experiments

All oligonucleotides were obtained from Genewiz.

Name	Protospacer	Target Residue
HDAC1_KO_sgRNA	GCACCGGGCAACGTTACGAA	I305
HDAC2_KO_sgRNA	TACAACAGATCGTGTAATGA	M195
KBTBD4_KO_sgRNA	GATATCTGTGAGTAAGCGGT	L190
LSD1_KO_sgRNA	TAGGGCAAGCTACCTTGTTA	N660
sgControl	CCCGGCGCCATTCTATCCGC	Luciferase (Control)

Supplementary Table 2 | Primer sequences for KO experiments

All primers were obtained from Sigma-Aldrich.

Name	Primer sequence	Target
HDAC1_sg2_SF3	ACACTCTTTCCCTACACGACGCTCTTCCGATCTNNNNCCAGGTAGCACAAGGATGGG	HDAC1 I305
HDAC1_sg2_SR3	TGGAGTTCAGACGTGTGCTCTTCCGATCTATATGACCAACGGGGAAGGG	
HDAC2_sg2_SF1	ACACTCTTTCCCTACACGACGCTCTTCCGATCTNNNNACTGATCTCCTAGGTTCATCTCA	HDAC2 M195
HDAC2_sg1_SR1	TGGAGTTCAGACGTGTGCTCTTCCGATCTACCAAAACTTATGGATTTGTTTTCAGG	
KBTBD4_sg4_SF1	ACACTCTTTCCCTACACGACGCTCTTCCGATCTNNNNGTAAACTAACACCAGGAAGCTTGA	KBTBD4 L190
KBTBD4_sg4_SR1	TGGAGTTCAGACGTGTGCTCTTCCGATCTCCAAGCACTGTGCCAAGA	
LSD1_sgN660_SF1	ACACTCTTTCCCTACACGACGCTCTTCCGATCTNNNNTGCCACCTCTCCCTGAGTGGA	LSD1 N660
LSD1_sgN660_SR1	TGGAGTTCAGACGTGTGCTCTTCCGATCTTGCAAGGCCACCTCCTCACC	

Primer binding sequence

Overhang for amplification with P5 site primer

Overhang for amplification with P7 site primer

Supplementary Table 3 | sgRNA for base editing validation

All oligonucleotides were obtained from Genewiz.

Vector	Gene	sgRNA	Protospacer	Predicted amino acid edits
pRDA_478	HDAC1	sgG202-2	TACTCTCCATACTTATGAAA	Glu203Lys;Gly202Lys
	HDAC1	sgT208	AGGAACTGGGGACCTACGGG	Thr208Ile
pRDA_479	HDAC1	sgD99	AGGACTGTCCAGTATTCGAT	Asp99Gly
	HDAC1	sgY204-1	GGAAGTACTCTCCATACTTA	Phe205Leu;Tyr204His
	HDAC1	sgL211	ACCCGTAGGTCCCCAGTTCC	Leu211Pro
SpG Cas9 BE3.9max	KBTBD4	sgL79	TGGTCCTCTCAGCTCAGAGC	Val78Val;Leu79Phe
	KBTBD4	sgR87-2	TGGATCGGAAGAAGCAGCTC	Arg87Gln
	KBTBD4	sgT91-3	TTCACTTCCAACCTGAAGGA	Thr91Ile;Ser92Phe
	KBTBD4	sgE96	GGCCTCCTTCAGGTTGGAAG	Glu96Lys;Lys95Lys
	KBTBD4	sgS138	GGTGTCAGACATGTATCAGC	Ser138Leu
	KBTBD4	sgT144-2	TGACATCTCTCTTTGAGGAA	Thr144Ile;Ser145Phe
	KBTBD4	sgR156-2	ACTGTGCGGGCCAAAAACCG	Arg156His
	KBTBD4	sgM167	CAGCCACATCACCTGAAGGC	Trp168Ter;Met167Ile
	KBTBD4	sgR172-2	AGATCGGCACAGTGATCCTG	Arg172Trp;His173Tyr
	KBTBD4	sgS212-2	CATCTCGGGTAAGTTTAGGG	Ile211Ile;Ser212Leu
	KBTBD4	sgS218-2	GTGTTCTCAGAACCCAACAG	Ser218Phe;Gln219Ter
	KBTBD4	sgA296	GCAGGCCGCAGTGATCTGGT	Ala296Thr;Ala295Ala
	KBTBD4	sgP311	CATCCCACGGCGCATGTGGA	Ile310Ile;Pro311Leu; Arg312Trp
	KBTBD4	sgD333	AGCCGGTCCCGAGGCAAAGG	Arg334Gln; Asp333Asn
	KBTBD4	sgQ417	CTCATCCAGTGCTTTGACAC	Ile416Ile;Gln417Ter
pU6-sgRNA (for eVLPs)	HDAC1	sgE203	GAGAGTACTTCCCAGGAACT	Glu203Gly;Tyr204Cys
	HDAC2	sgE204	GGGGAATACTTTCCTGGCAC	Glu204Gly;Tyr205Cys
	HEK3	sgHEK3	GGCCCAGACTGAGCACGTGA	-

Supplementary Table 4 | Primer sequences for base editing validation

All primers were obtained from Sigma-Aldrich.

Name	Primer sequence	Target
HDAC1_sgE98-SF1	ACACTCTTTCCCTACACGACGCTCTTCCGATCTNNNNTGTGGAAACTAACCTTTTGCT	HDAC1 sgD99
HDAC1_sgE98-SR1	TGGAGTTCAGACGTGTGCTCTTCCGATCTTTCCTTACCCACAGAACCAC	
HDAC1_sg202_SF1	ACACTCTTTCCCTACACGACGCTCTTCCGATCTNNNNCCAGAGGGTGCTGTACATTGA	HDAC1 sgG202-2, sgY204, sgT208, sgL211
HDAC1_sg202_SR1	TGGAGTTCAGACGTGTGCTCTTCCGATCTAGAGAAGTAGAGCTGCCAGC	
HDAC1_sgE203_SF1	ACACTCTTTCCCTACACGACGCTCTTCCGATCTNNNNGGGTCATGACTGTGTCCTTT	HDAC1 sgE203
HDAC1_sgE203_SR1	TGGAGTTCAGACGTGTGCTCTTCCGATCTGAGAGAAGTAGAGCTGCCAG	
HDAC2_sgE204_SF1	ACACTCTTTCCCTACACGACGCTCTTCCGATCTNNNNACTGATCTCCTAGGTTCATCTCA	HDAC2 sgE204
HDAC2_sgE204_SR1	TGGAGTTCAGACGTGTGCTCTTCCGATCTACCAAAACTTATGGATTTGTTTTCAGG	
KBTBD4_sg296_GSF1	ACACTCTTTCCCTACACGACGCTCTTCCGATCTNNNNGCACTGTGCAGAAGATGACT	KBTBD4 sgA295
KBTBD4_sg296_GSR1	TGGAGTTCAGACGTGTGCTCTTCCGATCTCCAGTCAACGGTGGCATT	
KBTBD4_sg311_314_GSF1	ACACTCTTTCCCTACACGACGCTCTTCCGATCTNNNNAGTTTGTGCCACCAGATCAC	KBTBD4 sgP311
KBTBD4_sg311_314_GSR1	TGGAGTTCAGACGTGTGCTCTTCCGATCTCACCCAGTGAATATATGGCATCTT	
KBTBD4_sg334_GSF1	ACACTCTTTCCCTACACGACGCTCTTCCGATCTNNNNCACCCAGTGAATATATGGCATCTT	KBTBD4 sgD333
KBTBD4_sg334_GSR1	TGGAGTTCAGACGTGTGCTCTTCCGATCTAGTTTGTGCCACCAGATCAC	
HEK3_SF1	ACACTCTTTCCCTACACGACGCTCTTCCGATCTNNNNTGCATTTGTAGGCTTGATGCT	sgHEK3

HEK3_SR1	TGGAGTTCAGACGTGTGCTCTTCCGATCTGGAGCTGCACATACTAGCCC	

Primer binding sequence

Overhang for amplification with P5 site primer

Overhang for amplification with P7 site primer

Supplementary Methods

Synthetic procedure

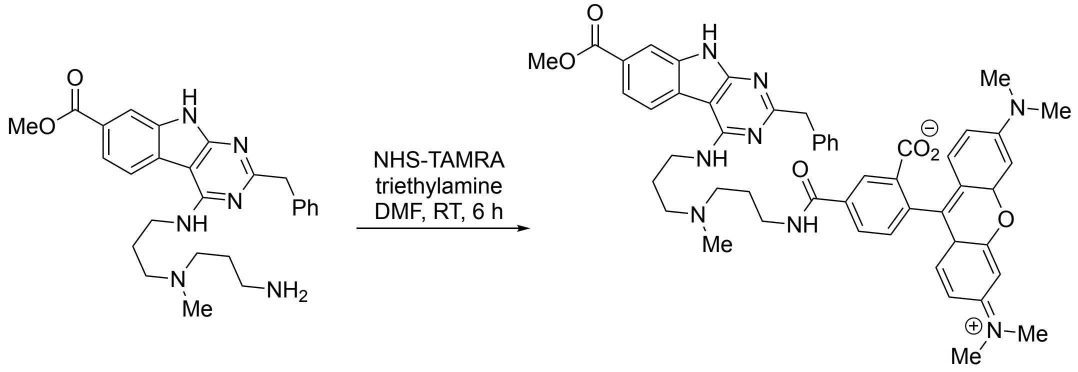

JL-1:

Methyl 4-((3-((3-aminopropyl)(methyl)amino)propyl) amino)-2-benzyl-9H-pyrimido-4,5-bindole-7-carboxylate (0.0120 g, 0.0261 mmol) was dissolved in DMF (0.5 mL) and triethylamine (10.8 uL, 0.0783 mmol), then TAMRA-NHS (0.0137 g, 0.0261 mmol) was added. The reaction mixture stirred for 6 h at RT. After evaporation to dryness, the residue underwent purification via C18 silica gel column chromatography, yielding U84-TAMRA (60%, 0.0137 g) as a purple solid. ^{1}H NMR (400 MHz, METHANOL-*D*$_4$) δ 8.59 (s, 1H), 8.49 (bs, 1H), 7.95 (s, 2H), 7.89 (d, *J* = 8.2 Hz, 1H), 7.47 (d, *J* = 8.2 Hz, 1H), 7.38 (d, *J* = 7.7 Hz, 2H), 7.29 - 7.25 (m, 4H), 7.20 - 7.12 (m, 2H), 7.05 (d, *J* = 9.3 Hz, 2H), 6.70 (d, *J* = 7.5 Hz, 2H), 6.43 (s, 2H), 4.09 (s, 2H), 3.79 (s, 3H), 3.52 – 3.47 (m, 4H), 3.21 - 2.96 (m, 18H), 2.70 (s, 3H), 2.03 (dm, *J* = 42.1 Hz, 4H). ^{13}C NMR (101 MHz, METHANOL-D4) δ 169.60, 168.75, 168.11, 161.93, 158.68, 158.43, 158.38, 158.15, 140.46, 137.21, 136.77, 136.53, 133.14, 133.04, 132.28, 131.35, 130.40, 130.05, 129.93, 129.76, 129.58, 129.50, 129.40, 127.46, 126.59, 125.05, 122.84, 121.10, 114.91, 114.59, 113.33, 97.10, 95.52, 55.25, 55.05, 52.54, 46.72, 40.74, 39.71, 38.52, 38.05, 25.91, 25.66. HRMS-ESI [M-H]: 871.3940 and observed: 871.3962.

NMR Spectra of JL-1

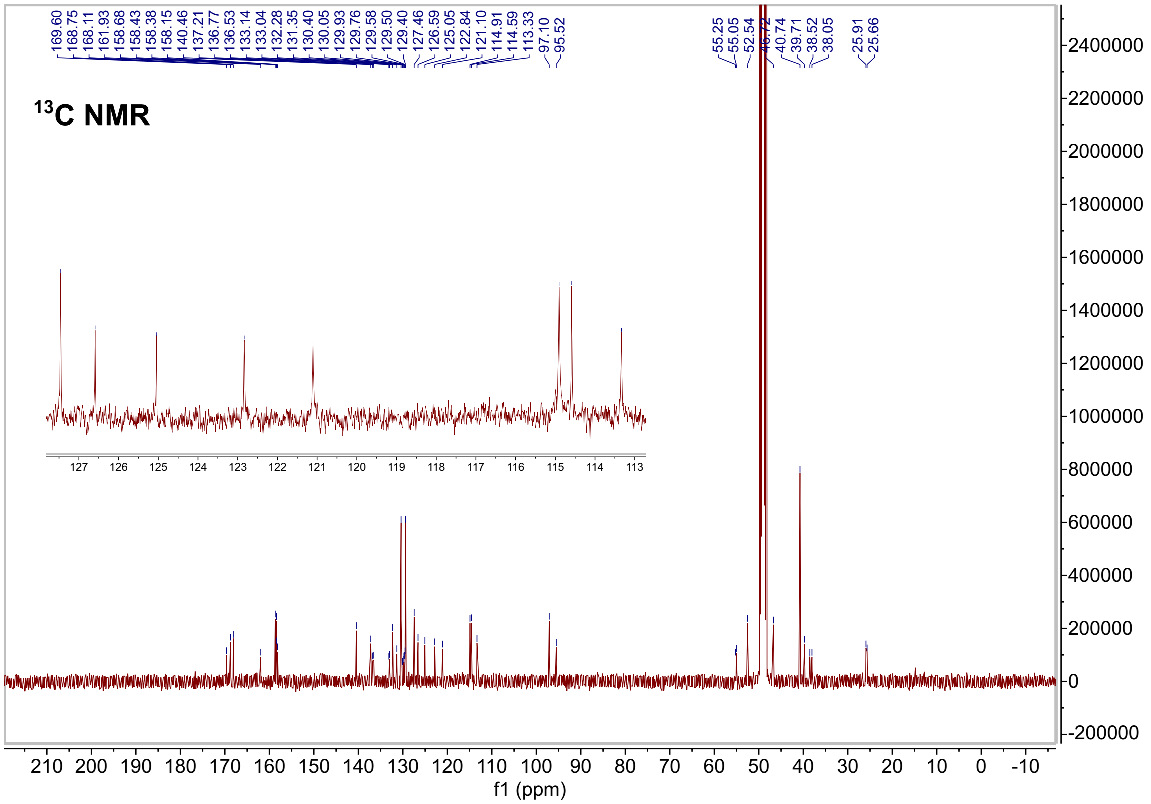

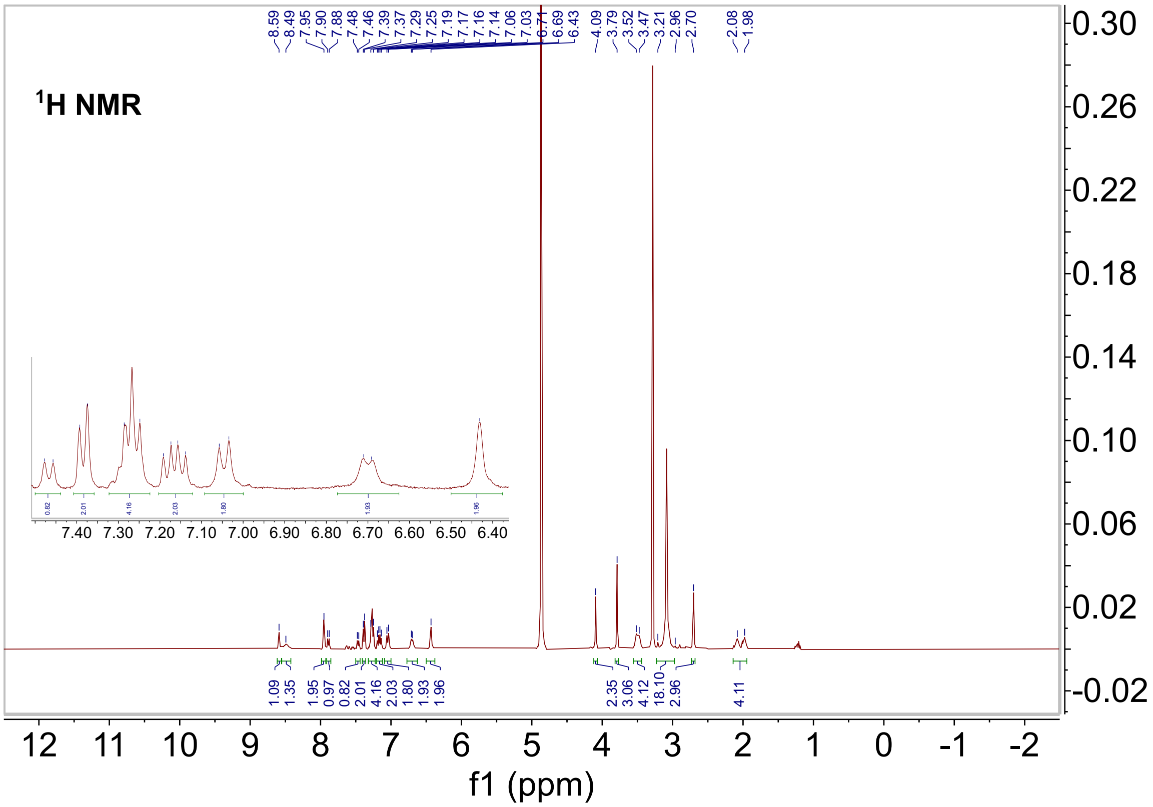

Supplement: Supplementary Information [file NIHMS2059347-supplement-Supplementary_Information.pdf]
