# Supplementary material for: UM171 glues asymmetric CRL3–HDAC1/2 assembly to degrade CoREST corepressors
Source: Nature. Author manuscript; Available in PMC 2025 Mar 24. (PMC11882444; doi:10.1038/s41586-024-08532-4)
Supplement: Supplementary Data Guide [file NIHMS2059347-supplement-Supplementary_Data_Guide.docx]

**Supplementary Data Legends**

Supplementary Data 1-14 are supplied as separate Excel files

**Supplementary Data 1 | SET-2 proteomics**

Proteins identified from SET-2 cells treated with UM171 or DMSO (shown in Figure 1b). An empirical Bayes-moderated t test was used to compare treatment groups, using the limma R package.

**Supplementary Data 2 | MV4;11 proteomics**

Proteins identified from MV4;11 cells treated with UM171 or DMSO (shown in Extended Data Figure 1a). An empirical Bayes-moderated t test was used to compare treatment groups, using the limma R package.

**Supplementary Data 3 | LSD1 co-IP/MS**

LSD1 co-IP/MS data from Waterbury *et al.*, *Mol. Cell*, **2024**, related to Figure 1b and Extended Data Figure 1a.

**Supplementary Data 4 | UM171 proteomics in SET-2**

Proteins enriched and depleted upon UM171 treatment in SET-2 cells (used in Supplementary Data 6). An empirical Bayes-moderated t test was used to compare treatment groups, using the limma R package. *P* values associated with every modified peptide or protein were adjusted using the Benjamini–Hochberg FDR approach.

**Supplementary Data 5 | UM171 ubiquitylome in SET-2**

K-ε-GG peptides enriched and depleted upon UM171 treatment in SET-2 cells (used in Supplementary Data 6). An empirical Bayes-moderated t test was used to compare treatment groups, using the limma R package. *P* values associated with every modified peptide or protein were adjusted using the Benjamini–Hochberg FDR approach.

**Supplementary Data 6 | UM171 ubiquitylome normalized against proteome**

K-ε-GG peptide data (Supplementary Data 5) normalized to the global proteome data (Supplementary Data 4), shown in Figure 1d. An empirical Bayes-moderated t test was used to compare treatment groups, using the limma R package. *P* values associated with every modified peptide or protein were adjusted using the Benjamini–Hochberg FDR approach.

**Supplementary Data 7 | HDAC1 ABE sgRNA library sequences and annotations**

sgRNA sequences and annotations used for HDAC1 ABE scanning.

**Supplementary Data 8 | HDAC1 CBE sgRNA library sequences and annotations**

sgRNA sequences and annotations used for HDAC1 CBE scanning.

**Supplementary Data 9 | HDAC1 ABE scanning data**

Source Data for HDAC1 ABE scanning (Figure 5b): raw read counts and (log_2_ + 1)-transformed sgRNA read-count normalized reads.

**Supplementary Data 10 | HDAC1 CBE scanning data**

Source Data for HDAC1 CBE scanning (Figure 5b): raw read counts and (log_2_ + 1)-transformed sgRNA read-count normalized reads.

**Supplementary Data 11 | KBTBD4 ABE sgRNA library sequences and annotations**

sgRNA sequences and annotations used for KBTBD4 ABE scanning.

**Supplementary Data 12 | KBTBD4 CBE sgRNA library sequences and annotations**

sgRNA sequences and annotations used for KBTBD4 CBE scanning.

**Supplementary Data 13 | KBTBD4 ABE scanning data**

Source Data for KBTBD4 ABE scanning (Figure 5e): raw read counts and (log_2_ + 1)-transformed sgRNA read-count normalized reads.

**Supplementary Data 14 | KBTBD4 CBE scanning data**

Source Data for KBTBD4 CBE scanning (Figure 5e): raw read counts and (log_2_ + 1)-transformed sgRNA read-count normalized reads.
